# Functional Diversity of Human Basic Helix-Loop-Helix Transcription Factor TCF4 Isoforms Generated by Alternative 5′ Exon Usage and Splicing

**DOI:** 10.1371/journal.pone.0022138

**Published:** 2011-07-15

**Authors:** Mari Sepp, Kaja Kannike, Ave Eesmaa, Mari Urb, Tõnis Timmusk

**Affiliations:** Department of Gene Technology, Tallinn University of Technology, Tallinn, Estonia; George Mason University, United States of America

## Abstract

**Background:**

Transcription factor 4 (TCF4 alias ITF2, E2-2, ME2 or SEF2) is a ubiquitous class A basic helix-loop-helix protein that binds to E-box DNA sequences (CANNTG). While involved in the development and functioning of many different cell types, recent studies point to important roles for TCF4 in the nervous system. Specifically, human *TCF4* gene is implicated in susceptibility to schizophrenia and *TCF4* haploinsufficiency is the cause of the Pitt-Hopkins mental retardation syndrome. However, the structure, expression and coding potential of the human *TCF4* gene have not been described in detail.

**Principal Findings:**

In the present study we used human tissue samples to characterize human *TCF4* gene structure and *TCF4* expression at mRNA and protein level. We report that although widely expressed, human *TCF4* mRNA expression is particularly high in the brain. We demonstrate that usage of numerous 5′ exons of the human *TCF4* gene potentially yields in TCF4 protein isoforms with 18 different N-termini. In addition, the diversity of isoforms is increased by alternative splicing of several internal exons. For functional characterization of TCF4 isoforms, we overexpressed individual isoforms in cultured human cells. Our analysis revealed that subcellular distribution of TCF4 isoforms is differentially regulated: Some isoforms contain a bipartite nuclear localization signal and are exclusively nuclear, whereas distribution of other isoforms relies on heterodimerization partners. Furthermore, the ability of different TCF4 isoforms to regulate E-box controlled reporter gene transcription is varied depending on whether one or both of the two TCF4 transcription activation domains are present in the protein. Both TCF4 activation domains are able to activate transcription independently, but act synergistically in combination.

**Conclusions:**

Altogether, in this study we have described the inter-tissue variability of TCF4 expression in human and provided evidence about the functional diversity of the alternative TCF4 protein isoforms.

## Introduction

TCF4 (Gene 6925), alias ITF2 (immunoglobulin transcription factor 2), SEF2 (leukemia virus SL3-3 enhancer factor 2), E2-2 and ME2 (mouse E2), is one of the widely expressed class A basic helix-loop-helix (bHLH) transcription factors (TFs) that are homologous to *Drosophila melanogaster* protein daughterless (Gene 34413) [1], [2]. The bHLH factor TCF4 discussed here should not be confused with the high mobility group box transcription factor 7-like 2 (TCF7L2; Gene 6934) that is a downstream effector of the β-catenin signaling pathway and is also known as TCF4 (T-cell specific factor 4).

Class A bHLH factors in mammals include TCF4, HEB (TCF12; Gene 6938) and E2A (TCF3, ITF1; Gene 6929) alternative isoforms E12 and E47 [3]. These proteins are referred to as E-proteins since they bind to Ephrussi box (E-box) sequence (CANNTG) as homodimers or as heterodimers with tissue-specific bHLH factors [3], [4]. Dimerization is mediated by the C-terminal HLH motif that together with the preceding stretch of basic amino acids is required also for DNA binding. Structurally related Id proteins (inhibitors of differentiation) hinder DNA binding of E-proteins by heterodimerization, whereas Ca^2+^-calmodulin specifically inhibits DNA binding of E-protein homodimers [5]–[7]. Three amino-terminally distinct TCF4 isoforms have been described – TCF4-A, TCF4-B and TCF4-D [2]. All these isoforms contain the bHLH domain and a transcription activation domain (AD2) [8]. TCF4-B has an additional transcription activation domain in its N-terminus (AD1) [9].

In Drosophila the only E-protein, daughterless, is involved in sex determination and neurogenesis [10], [11]. In mammals, substantial functional overlap among E-proteins has hampered deciphering their exact roles. However, it is known that TCF4 is required for postnatal survival in mice [12], [13] and has many cell lineage specific functions. For instance, TCF4 regulates development of B-, T- and plasmacytoid dendritic cells [13]–[15], development of Sertoli cells [16] and pontine nucleus neurons [17], myogenesis [18], melanogenesis [19] and epithelial-mesenchymal transition [20]. The importance of TCF4 in human nervous system development is underscored by the association of a TCF4 allele with schizophrenia [21] and identification of *TCF4* haploinsufficiency as the cause for Pitt-Hopkins syndrome (OMIM 610954), a rare disease featuring mental retardation, hyperventilation and seizures [22]–[24].

In this study we show that *TCF4* is widely, but not equally expressed and its levels are particularly high in the nervous system. We demonstrate that usage of alternative 5′ exons for transcribing the human *TCF4* gene potentially yields in numerous TCF4 protein isoforms that differ in their subcellular localization and capacity to activate transcription.

## Results

### *TCF4* gene contains many mutually exclusive 5′ exons

**Figure 1:**
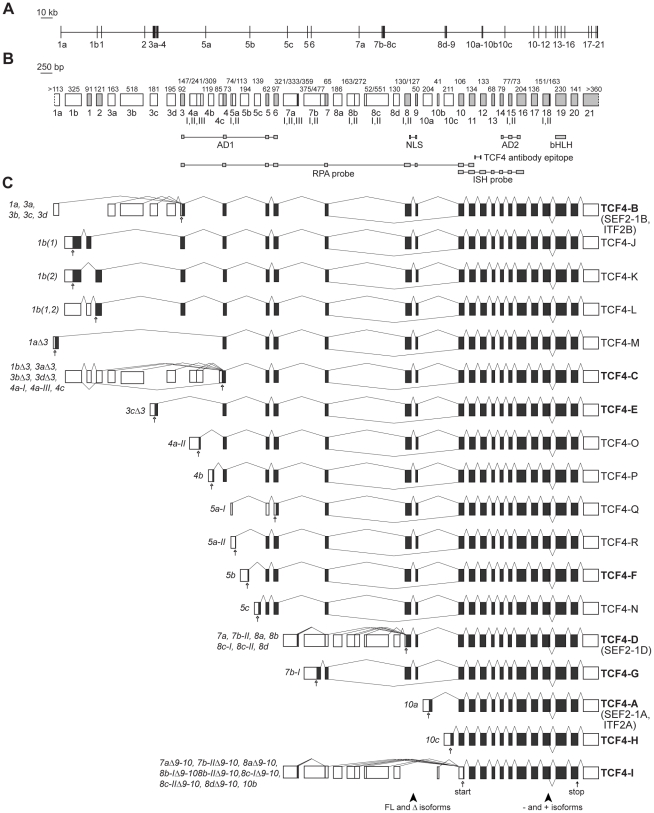
Structure and alternative splicing of the human *TCF4* gene. *TCF4* genomic organization with (**A**) introns drawn in scale or (**B**) exons drawn in scale. White boxes mark 5′ exons and light grey boxes represent internal or 3′ exons. Exon names are shown below the boxes. Roman numerals designate alternative splice donor or acceptor sites. Numbers above the exons indicate their sizes in bps. The regions encoding the respective domains of TCF4, the NLS identified in this study and the epitope of the used TCF4 antibody are indicated below the gene structure. Locations of RPA and ISH riboprobes used in this study are also shown. AD, transcription activation domain; bHLH, basic helix-loop-helix domain; NLS, nuclear localization signal; RPA, ribonuclease protection assay; ISH, *in situ* hybridization. (**C**) *TCF4* alternative transcripts grouped together according to the encoded TCF4 protein isoform. Translated and untranslated regions are indicated as dark grey and white boxes, respectively. Transcripts are designated with the name of the 5′ exon and, if needed, with the number of the splice site used in the 5′ exon. Excluded internal exons are shown with the symbol Δ and included internal exons in parentheses, if necessary. The names of the protein isoforms are shown at the right. The isoforms cloned in this study are brought in bold. The position of the first in-frame start codon for each transcript and stop codon are shown with empty and filled arrows, respectively. Arrowheads at the bottom of the panel point to the regions of alternative splicing giving rise to full-length (FL) and Δ, − and + isoforms.

To describe the structure and alternative splicing of the human *TCF4* gene we performed bioinformatic analysis of mRNA and expressed sequence tag (EST) sequences available in public databases and sequences of RT-PCR products from this study. In estimation of transcription start sites we relied on publicly available data from sequencing of oligo-cap, cap-trapping and SMART cDNA libraries [25]–[28]. *TCF4* gene is located on chromosome 18q21.2 and spans 437 kbs. It has 41 exons of which 21 are alternative 5′ exons situated at various positions throughout the gene (Figure 1). In this study *TCF4* exons are named as follows: initial 5′ exons are designated with a lowercase letter preceded by a number that shows the following internal exon in the gene; internal exons are numbered from 1 to 20; exon 21 is the only terminal 3′ exon. *TCF4* transcripts are named according to the initial exon they contain. Initial exons 1a and 1b are located upstream of internal exon 1. Exons 3a–3d precede and 4a–4c follow internal exon 3. 4c is a 5′ extension of internal exon 4. There are three 5′ exons (5a–5c) in front of exon 5; two (7a, 7b) in front of exon 7; four (8a–8d) in front of exon 8; and three (10a–10c) in front of exon 10. Generally, 5′ exons are spliced together with the next internal exon in the gene, apart from the cases when the following internal exon is a cassette exon and can be skipped (exons 1 or 2, exon 3, exons 8–9; see below). As an exception 5′ exon 1a is never used together with internal exons 1 or 2 and is always joined to internal exon 3 or 4. We identified several alternative splice donor sites in the 5′ exons: two (I–II) in exons 5a, 8b and 8c; three (I–III) in exons 4a and 7a. In case of exon 7b the intron between 7b-I and internal exon 7 can be retained giving rise to 7b-II transcripts. Different usage of splice sites sometimes affects the coding potential of a transcript (Figure 1C). In addition, the reading frame shifts with alternative splicing of internal cassette exons 1 or 2 and exon 3. Examination of open reading frames and search for possible translation start codons demonstrated that, altogether, *TCF4* transcripts potentially code for 18 N-terminally distinct protein isoforms named TCF4-A – TCF4-R (Figures 1C and S1) of which only isoforms TCF4-A, TCF4-B and TCF4-D have been described previously as SEF2-1A or ITF2-A, SEF2-1B or ITF2-B, and SEF2-1D, respectively [2], [18]. Most of the TCF4 isoforms have a stretch of unique amino acids in their N-termini and the whole aminoterminal transactivation domain AD1 (coded by exons 3–6) is present only in isoforms with the longest N-termini such as TCF4-J, -K, -L and TCF4-B (Figures 1C and S1). Other TCF4 isoforms contain parts of AD1 or are completely devoid of it. More precisely, isoforms TCF4-C, -E, -M, -O and -P contain a region of AD1 coded by exons 4–6, isoforms TCF4-F, -N and -R contain a region coded by exons 5–6, isoform TCF4-Q has only a short sequence coded by exon 6, and the rest of the isoforms (TCF4-D, -G, -A, -H and -I) lack AD1.

Apart from differences in the N-termini, the number of TCF4 isoforms is increased by in-frame alternative splicing of internal exons (Figures 1C and S1). Firstly, simultaneous skipping of internal cassette exons 8 and 9 gives rise to TCF4 Δ isoforms, as opposed to full-length isoforms that contain the amino acids coded by exons 8–9. Secondly, at exon 18 there are two alternative splice donor sites that enable to splice in or out a 12 bps sequence encoding amino acid sequence RSRS present in + isoforms and absent in − isoforms. Thirdly, exons 8 and 15 contain two alternative splice acceptor sites that lead to optional inclusion of the first three nucleotides (CAG) of an exon in mRNA and a glutamine or alanine residue, respectively, in the corresponding position in protein sequence (Figure S1). All TCF4 isoforms, regardless of their N-terminal or internal differences, contain transactivation domain AD2 (coded by exons 14–16) and the bHLH domain (coded by exon 19).

The overall structure of *TCF4* gene is conserved in the mouse genome. The identity between human and mouse sequences for internal exons 3–20 and 3′ exon 21 is 92%. Out of the 21 *TCF4* 5′ exons in human genome at least 13 are also transcribed in mouse as assessed by the presence of respective ESTs in public databases (Table S1). Sequences of most human 5′ exons align to the respective region in the mouse genome with approximately 70–99% identity (Figure S2). Exons 1b, 3c, 5b and 5c are more divergent and for exons 1a, 1 and 2 no alignment between human and mouse genes was obtained. Five out of the seven non-conserved human *TCF4* exons indicated above originate from exonization of various transposable elements. Namely, exon 1a overlaps with two LTR repeats, exon 1b overlaps with DNA transposon MER5B, exon 1 consists of SINE (Alu) and LINE repeat sequences, exon 2 consists of Alu repeat sequence and exon 5c consists of SINE (MIR) element sequence. In addition, 5′ exon 3a immediately follows a SINE (MIR) element in the genome (Figure S2).

**Figure 2:**
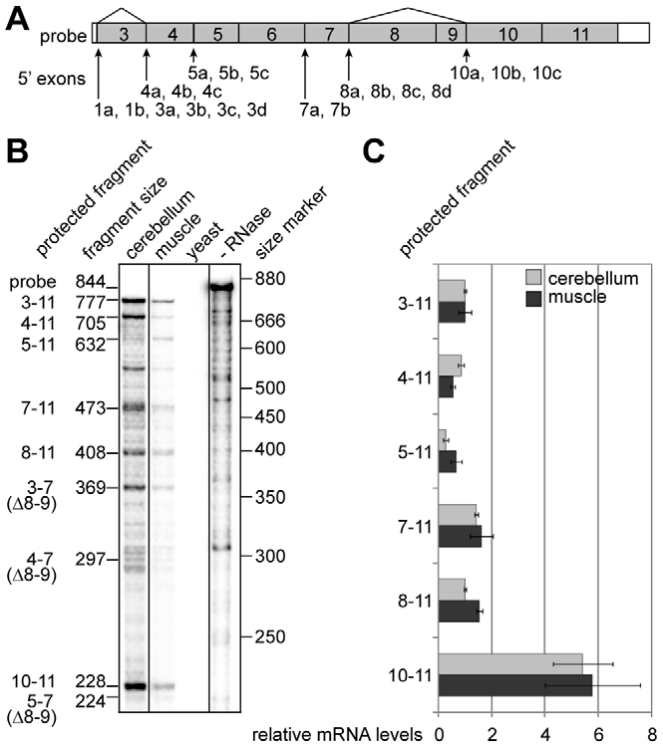
Initiation of transcription from alternative sites within the *TCF4* gene. (**A**) Schematic representation of the ribonuclease protection assay probe complementary to *TCF4* exons 3–11. Location of the *TCF4* 5′ exons relative to the probe is shown with arrows and the sites of alternative splicing with lines. (**B**) Autoradiograph of the probe fragments protected by human cerebellum or muscle RNA and fragments obtained from control reactions with yeast RNA or without RNase treatment. The expected sizes of the protected fragments in bps and the exons they span are shown at the left and the location of the size markers at the right. (**C**) Densitometric quantification of the protected fragments in B from two assays. The values are given in relation to the levels of the fragment spanning exons 3–11 for both tissues. Error bars indicate standard deviations.

In order to determine the relative abundance of mRNAs initiated at different positions within the *TCF4* gene, we carried out ribonuclease protection assays with the probe spanning internal exons 3–11 (Figure 2A). Transcripts containing different number of internal exons were detected in human cerebellum and muscle (Figure 2B). The longest protected fragment, containing exons 3–11, corresponds to transcripts initiated at 5′ exons 1a, 1b and 3a–3d. The fragment comprising exons 4–11 represents the sum of transcripts containing the above mentioned 5′ exons in case of exon 3 skipping and transcripts initiated at 4a–4c. The fragments comprising exons 5–11, 7–11 and 8–11 rise from transcripts initiated at 5a–5c, 7a–7b and 8a–8d, correspondingly. The fragments comprising exons 3–7, 4–7 and 5–7 represent transcripts that are initiated at the same sites as fragments 3–11, 4–11 and 5–11, respectively, but that lack the cassette exons 8–9 as a result of alternative splicing. All Δ8–9 transcripts additionally give rise to the fragment containing exons 10–11 and this fragment also includes transcripts initiated at exons 10a–10c. Densitometric quantification of the protected fragments showed that the levels of *TCF4* transcripts spanning different number of internal exons were comparable in both human cerebellum and muscle (Figure 2C). From these data we concluded that transcription is initiated at relatively similar levels from alternative sites within the *TCF4* gene.

### *TCF4* mRNAs are ubiquitously but not equally expressed

**Figure 3:**
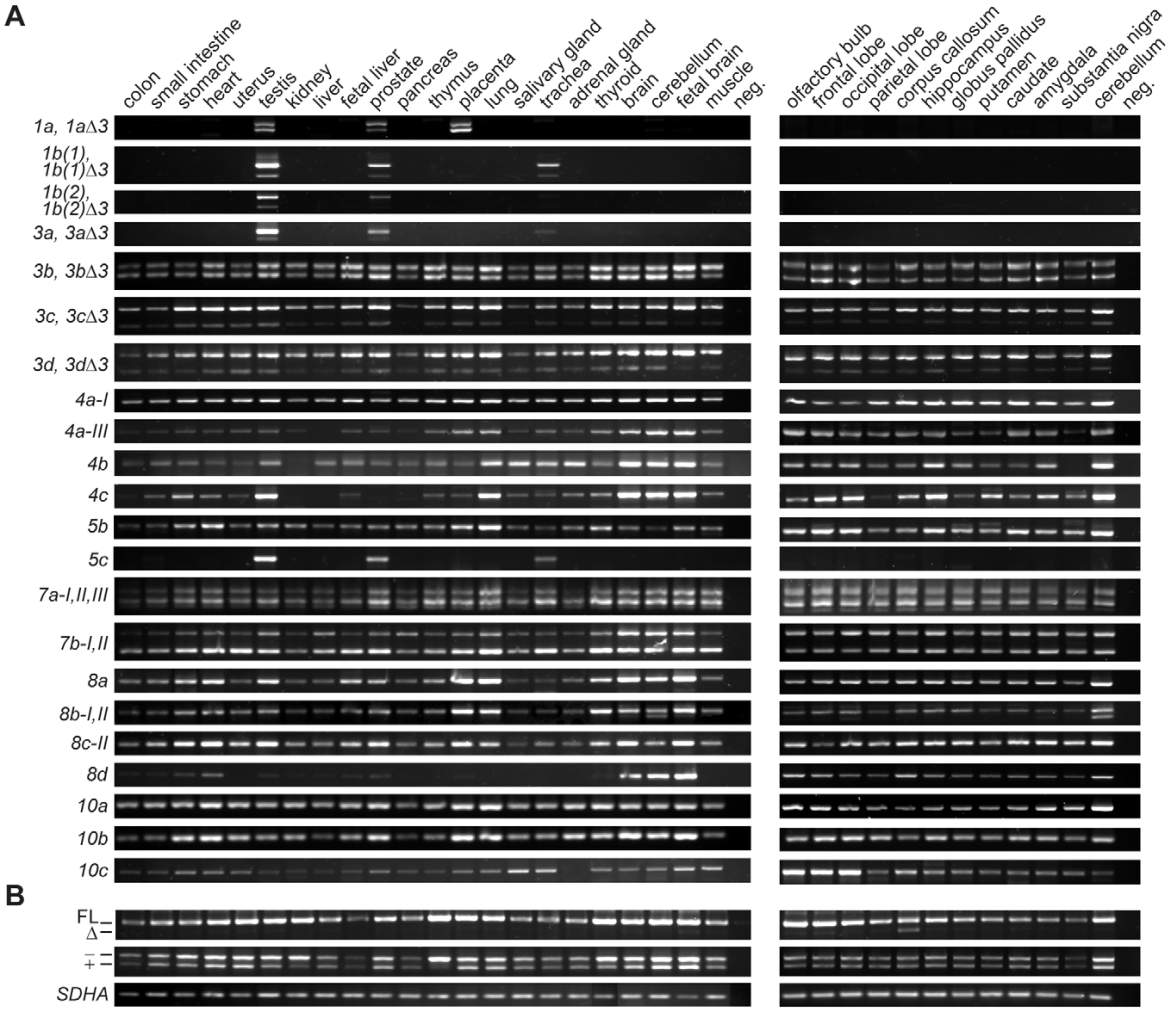
Expression of alternative *TCF4* mRNAs in human tissues and brain regions. (**A**) RT-PCR analysis of *TCF4* transcripts with different 5′ exons and (**B**) with alternative internal splicing. Transcripts are designated as in Figure 1C. The positions of bands respective to the transcripts encoding the full-length (FL) and Δ isoforms, − and + isoforms are indicated at the left on panel B. mRNAs with longer exon 18 (+) give rise to RT-PCR product that has a unique BglII restriction site enabling discrimination from RT-PCR products amplified from mRNAs not containing the 12 bps insert (−). House-keeping gene *SDHA* mRNA expression is shown at the bottom of the panel. PCR with no template was performed as a negative control (neg) with each primer pair.

We studied the usage of different 5′ exons in a variety of human tissues and brain regions by reverse transcription polymerase chain reaction (RT-PCR). Our results showed that although the majority of the alternative *TCF4* transcripts were present in most tissues analyzed, there were a few that had a more limited expression pattern (Figure 3A). For instance we detected transcripts containing exon 1a only in testis, prostate and placenta. The use of 5′ exons 1b, 3a and 5c was restricted to testis, prostate and trachea. Furthermore, there were several transcripts, most remarkably 8d transcripts, that featured considerably higher expression levels in the nervous system than in other tissues. RT-PCR analysis also provided information about the occurrence of alternative splicing. The skipping of exon 3 (Δ3) was a minor event in case of transcripts 1b, 3a, 3c, and 3d, whereas comparable levels of full-length and Δ3 mRNAs were present in case of transcripts 1a and 3b. Several general observations were made concerning the usage of alternative splice donor sites at 5′ exons. Firstly, the prevalence of splice site utilization at exon 7a decreased in the row of II, III, I. Secondly, the levels of 7b-I transcripts were higher than those of 7b-II transcripts in most of the tissues analyzed, except in the nervous system where nearly equal levels of 7b-I and 7b-II transcripts were detected. Thirdly, 8b-I transcripts were present only in the cerebellum, whereas in all other tissues and brain regions splice donor site II of exon 8b was exclusively used. Fourthly, splice site II was predominantly used at exon 8c. The levels of 5a and 8c-I transcripts were too low for reliable expression analysis; nevertheless, the respective PCR amplification products were consistently detected in the brain samples (data not shown). Our analysis did not reveal the usage of splice donor site II at exon 4a in any of the tissues studied.

Next, we examined in-frame alternative splicing of *TCF4* internal exons (Figure 3B). To monitor the splicing of cassette exons 8–9, we performed PCR with forward and reverse primers in exon 5 and 11, respectively. To evaluate the usage of two alternative splice donor sites at exon 18, we amplified the region spanning exons 10–20 and analyzed the PCR product by restriction with BglII, that has a unique recognition site in the 12 bp region present only in transcripts coding for the + isoforms. As shown in Figure 3B inclusion of exons 8–9 was prevalent and skipping of exons 8–9 a rare event in most tissues analyzed. Comparable amounts of full-length and Δ8–9 transcripts were present only in the *corpus callosum*. The levels of transcripts containing or lacking the extra 12 bps of exon 18 were roughly equal in most tissues analyzed (Figure 3B).

**Figure 4:**
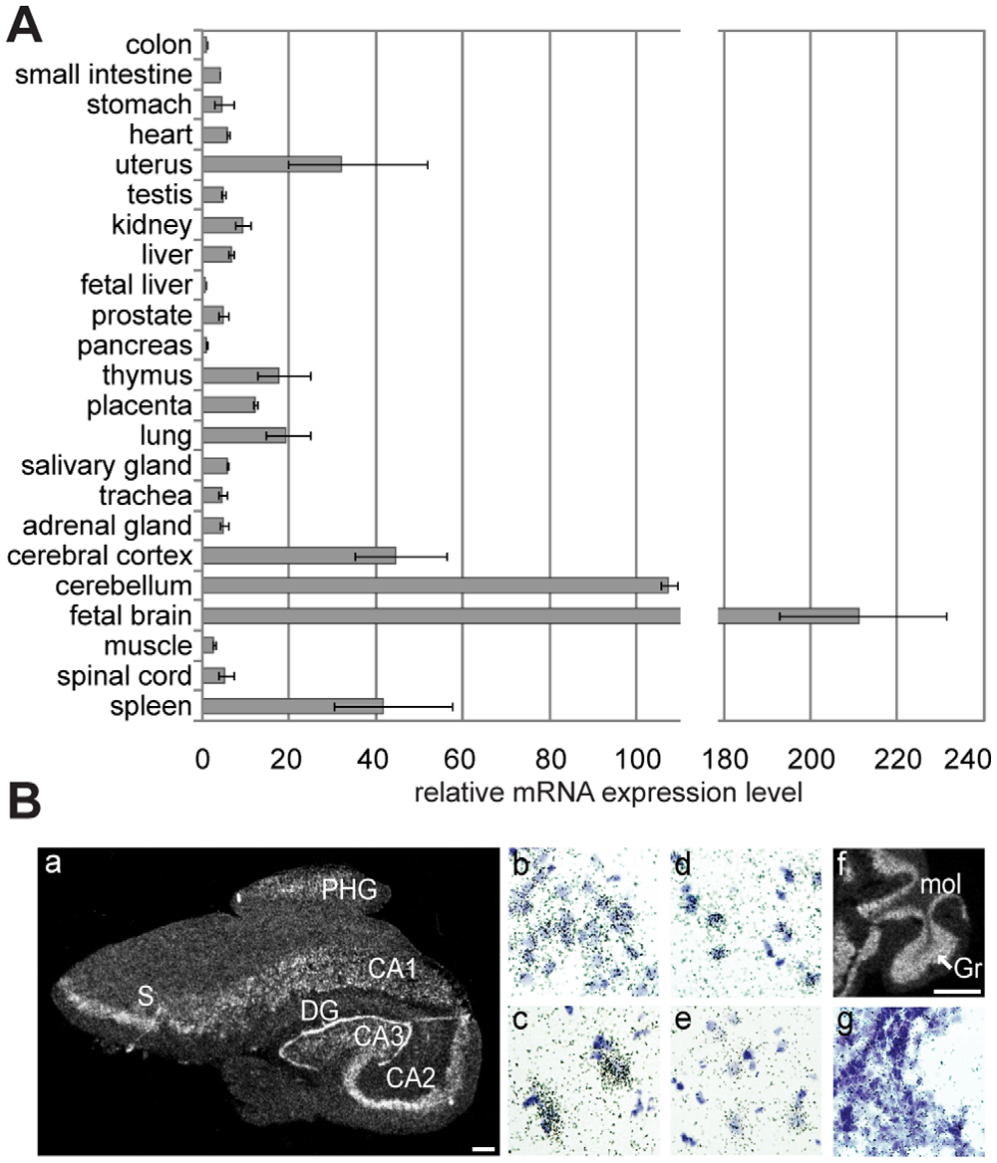
Inter-tissue variability of *TCF4* mRNA levels. (**A**) Quantitative RT-PCR analysis of *TCF4* expression in human tissues. Levels of *TCF4* transcripts were determined using three different primer pairs and the results were normalized to the expression levels of four house-keeping genes (*SDHA*, *HMBS*, *GAPDH* and *UBC*). Shown are the means relative to the TCF4 expression level measured in colon that was arbitrarily set as 1. Error bars indicate standard deviations. (**B**) *In situ* hybridization analysis of *TCF4* expression in human hippocampus and cerebellum. Autoradiographs from (**a**) a coronal section of the hippocampus and (**f**) a sagittal section of the cerebellum are shown. Scale bar 1 mm. Bright-field higher magnification images of emulsion-dipped and hematoxylin-stained sections of (**b**) the granular cell layer of the dentate gyrus, (**c**) pyramidal cell layer of the CA2 region, (**d**) subiculum and (**e**) PHG region of the cortex. CA1, CA2, CA3, respective regions of the hippocampus; DG, dentate gyrus; Gr, granular cell layer of the cerebellum; mol, molecular layer of the cerebellum; PHG, parahippocampal gyrus; S, subiculum.

To compare *TCF4* expression levels in different human tissues more precisely, we performed quantitative PCR with three pairs of primers designed to amplify all *TCF4* transcripts (products spanning exons 10–11, 17–18 or 19–20). The obtained relative values were normalized to the expression levels of four house-keeping genes as described in Materials and Methods. We found that *TCF4* mRNA levels were considerably elevated in fetal brain and adult cerebellum – approximately 200 and 100 times above the levels measured in colon, respectively (Figure 4A). Compared to the levels in colon, about 40-fold higher levels were detected in cerebral cortex and spleen and more than 10-fold higher levels were seen in uterus, lung, thymus and placenta. The lowest quantities of *TCF4* transcripts were present in fetal liver, pancreas and colon. From these results we concluded that although the expression of *TCF4* is ubiquitous, its levels vary considerably between tissues. Particularly, we turned our attention to high expression levels of *TCF4* in the nervous system and carried out *in situ* hybridization experiments on sections from human hippocampus and cerebellum to characterize *TCF4* expression at cellular level. As shown in Figure 4B *TCF4* mRNA was detected in hippocampal neurons in dentate gyrus and CA1-CA3 regions, neurons of subiculum and parahippocampal gyrus of the cortex, and cerebellar granule neurons.

### Several TCF4 protein isoforms are expressed in human tissues

**Figure 5:**
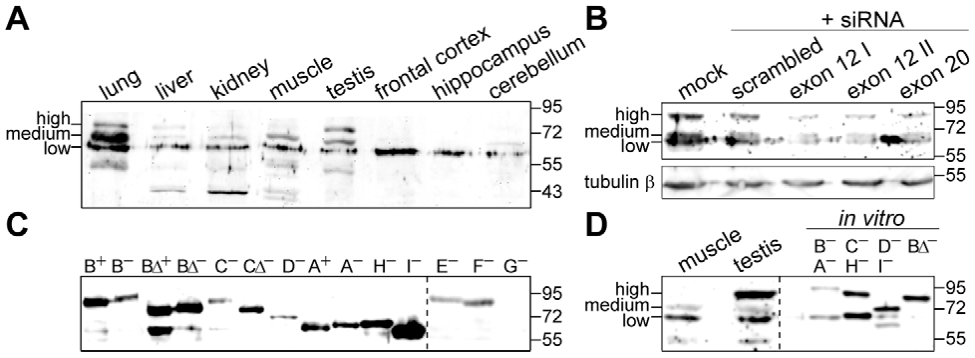
Expression of TCF4 protein isoforms. (**A**) Western blot analysis of different human tissues and brain regions with TCF4 antibodies. (**B**) The effect of TCF4 targeting siRNAs on the levels of proteins detected by TCF4 antibodies in extracts of Neuro2A cells. Three different siRNAs specific for *TCF4* exon 12 or 20 were transfected into Neuro2A cells; mock and scrambled siRNA transfections were performed in control. Tubulin β levels were determined to demonstrate equal loading. (**C**) Western blot analysis of TCF4 isoforms overexpressed in HEK293 cells using TCF4 antibodies. (**D**) Comparison of fractionation of proteins recognized by TCF4 antibodies in human muscle and testis extracts to *in vitro* translated selected human TCF4 isoforms. The localization of endogenous TCF4 with high, medium or low molecular weight is indicated at the left in A, B and D; the dashed line in C and D separates different exposures; molecular mass (in kDa) marker bands are shown at the right on all panels.

We next asked whether and which of the different TCF4 protein isoforms are translated *in vivo*. To address this question we monitored the expression of endogenous TCF4 isoforms in different human tissue extracts by western blotting with TCF4-specific antibodies that recognize an epitope present in all described TCF4 isoforms. As shown in Figure 5A multiple TCF4 isoforms were present in human lung, liver, kidney, muscle and testis. In these tissues we detected three prominent bands that were assigned as high, medium and low molecular weight (Mw) TCF4. In case of frontal cortex, hippocampus and cerebellum low molecular weight TCF4 band was predominant and the signals of medium and high Mw TCF4 were very low. The levels of high or medium Mw TCF4 were elevated relative to other forms of TCF4 in testis or lung, respectively. To validate the specificity of the TCF4 antibodies, we implemented a RNAi based approach in Neuro2A mouse neuroblastoma cells. Similarly to the human tissue extracts, high, medium and low Mw bands were detected in the lysates of Neuro2A cells by western blotting with the TCF4 antibodies. Compared to mock and scrambled siRNA transfected cells the levels of all three forms were reduced in cells transfected with three different siRNAs targeting *TCF4* exon 12 or 20 that are present in all described *TCF4* transcripts (Figure 5B), thus verifying that the TCF4 antibodies used in the current study specifically recognize TCF4 proteins.

In order to define the nature of the three bands, we cloned the coding regions of isoforms TCF4-A – TCF4-I into pCDNA mammalian expression vector. In addition, we cloned variants coding for + and − isoforms, full-length and Δ isoforms. We transfected the obtained plasmids into HEK293 cells and detected the overexpressed proteins by western blotting with TCF4-specific antibodies. The different TCF4 isoforms were expressed at variable levels (Figure 5C). The levels of isoforms TCF4-B^+^, -B^−^, -BΔ^+^, -BΔ^−^, -CΔ^−^, -A^+^, -A^−^, -H^−^ and -I^−^ were high, those of TCF4-C^−^ and -D^−^ medium, and those of TCF4-E^−^ and -F^−^ low. Expression of TCF4-G^−^ was not detected by western blotting. Although it was not possible to distinguish all isoforms from each other by size, we compared side by side the bands detected in human muscle and testis to different TCF4 isoforms translated *in vitro* (Figure 5D) and made the following conclusions about the nature of the three bands in human tissue extracts: the high Mw TCF4 fractionates similarly to TCF4-B and -C, the medium Mw TCF4 similarly to TCF4-D, and low Mw TCF4 to TCF4-A and TCF4-H. In addition, a few proteins with smaller molecular weight than any of the cloned TCF4 isoforms were detected in several tissues (Figure 5A). These could be non-specific signals, represent TCF4 proteolytic fragments or undescribed TCF4 isoforms. In sum, the above described *TCF4* gene structure and expression data demonstrate that multiple TCF4 isoforms are present in human tissues and suggest that the isoforms could potentially differ in their functional properties.

### TCF4 is imported to the nucleus due to its NLS or via a piggy-back mechanism

**Figure 6:**
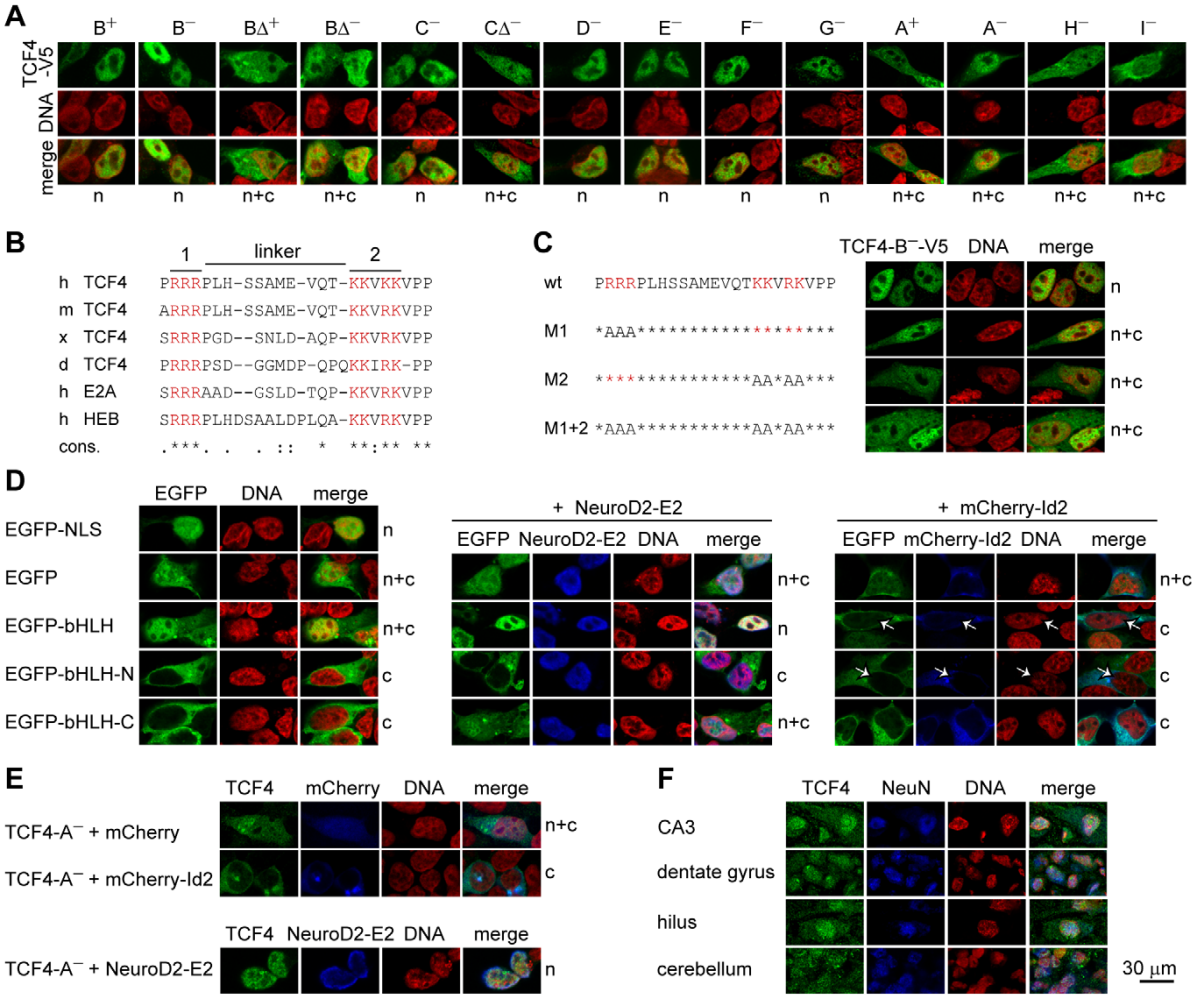
TCF4 intracellular localization. (**A**) Immunocytochemical analysis of V5-tagged TCF4 isoforms overexpressed in HEK293 cells. Isoforms analyzed are indicated at the top and localization pattern at the bottom of the panel. n, nuclear; c, cytoplasmic; n+c, nuclear and cytoplasmic. DNA was counterstained with DAPI (pseudocoloured red) to visualize nuclei. (**B**) Alignment of the identified bipartite NLS in TCF4 of *Homo sapiens* (h), *Mus musculus* (m), *Xenopus laevis* (x) and *Danio rerio* (d); in E2A and HEB of *Homo sapiens*. Two clusters of basic amino acids (in red, 1 and 2) and the linker are indicated with lines at the top. Conservation is indicated at the bottom. ‘*’, identity; ‘:’, conserved; ‘.’, semi-conserved substitution. (**C**) The effect of site-directed mutagenesis of the NLS on the localization of TCF4 in HEK293 cells. Basic amino acids in cluster 1 (M1), 2 (M2) or both (M1+2) were replaced with alanines in the context of TCF4-B^−^-V5 and the localization of the proteins was monitored by immunocytochemistry. (**D**) Localization of EGFP fusion proteins with TCF4 NLS, bHLH, N-terminal (N) or C-terminal (C) half of TCF4 bHLH domain in HEK293 cells. NeuroD2-E2 or mCherry-Id2 encoding plasmid was cotransfected when indicated. White arrows indicate cells expressing mCherry-Id2. (**E**) Effect of mCherry-Id2 or NeuroD2-E2 co-expression on subcellular distribution of TCF4-A^−^. The localization patterns of overexpressed TCF4 proteins are indicated at the right of the panels in C, D and E. (**F**) Immunohistochemical analysis of endogenous TCF4 in human hippocampal and cerebellar sections. NeuN staining was used to identify neurons.

To gain insight into possible functional variation amongst the TCF4 isoforms we first investigated, by indirect immunofluorescence, the intracellular localization of TCF4 isoforms overexpressed in HEK293 cells (Figure 6A). We observed two patterns of distribution – exclusively nuclear and nuclear plus cytoplasmic. Full-length TCF4 isoforms that have longer N-termini (TCF4-B^+^, -B^−^, -C^−^, -D^−^, -E^−^, -F^−^ and -G^−^) were restricted to cell nucleus whereas isoforms with shorter N-termini (TCF4-A^−^, -H^−^ and -I^−^) and Δ isoforms (TCF4-BΔ^+^, -BΔ^−^ and -CΔ^−^) localized to the nucleus and, in addition, to the cytoplasm.

Since all isoforms with exclusively nuclear distribution contain the amino acids coded by exons 8–9 and the isoforms with broader intracellular localization are devoid of these amino acids, we searched for possible nuclear localization signal (NLS) in this region. Using PSORT software we identified a potential bipartite NLS that contains two clusters of basic amino acids (RRR and KKVRK) separated by a linker of 11 amino acids. The two clusters are conserved in TCF4 of *Mus musculus*, *Xenopus laevis* and *Danio rerio*, and also in two other E-proteins E2A and HEB (Figure 6B). To validate the NLS we performed site-directed mutagenesis in the context of TCF4-B^−^ isoform and replaced the basic amino acids in cluster 1, 2 or both with alanines. As shown in Figure 6C mutagenesis of either cluster 1 (M1) or 2 (M2) produced a protein that is partly cytoplasmic, but even when both clusters were mutated (M1+2) a substantial portion of the protein remained nuclear, similarly to the genuine TCF4 isoforms not containing the region coded by exons 8–9. As a next step we fused the NLS to the C-terminus of EGFP and monitored its localization in HEK293 cells. EGFP-NLS fusion protein was confined to the nucleus whereas control EGFP was distributed diffusely all over the cell (Figure 6D). From these results we concluded that the identified NLS is functional, but additional region(s) responsible for nuclear import of isoforms lacking the amino acids coded by exons 8–9 exist in TCF4 protein.

Another region with high basic charge density is located in the DNA-binding domain of TCF4. To test whether this part of the protein is able to mediate nuclear import we constructed different fusion proteins where the entire bHLH domain or its N-terminal or C-terminal part is fused with EGFP. The fusion proteins were overexpressed in HEK293 cells and their localization was studied by direct EGFP fluorescence (Figure 6D). EGFP-bHLH was present in the nucleus and cytoplasm indicating that the bHLH domain was to some extent able to direct the protein to the nucleus, since in contrast to EGFP (29 kDa), the EGFP-bHLH (43 kDa) fusion protein cannot be transported to the nucleus by passive mechanisms due to its higher molecular mass. At the same time EGFP-bHLH-N (36 kDa) and EGFP-bHLH-C (37 kDa) were detected only in the cytoplasm, meaning that neither part alone was able to mediate nuclear import. These data led us to hypothesize that structural integrity of the bHLH domain is necessary for EGFP-bHLH nuclear localization mediated by heterodimerization with endogenous NLS bearing HLH-proteins. To test this assumption, we co-expressed EGFP and the described fusion proteins together with known TCF4 dimerization partners: E2-tagged NeuroD2 (Gene 18013), that features nuclear localization, and mCherry-fused Id2 (Gene 15902), that bears a nuclear export signal (NES) and is similarly to the described EGFP-Id2 fusion protein [29] predominantly cytoplasmic. The results of this assay showed that NeuroD2-E2 did not influence the distribution of EGFP and EGFP-bHLH-N, but was able to direct EGFP-bHLH and to lesser extent also EGFP-bHLH-C to the nucleus. Co-expression of mCherry-Id2 altered only the localization of EGFP-bHLH by excluding it from the nucleus (Figure 6D). We additionally monitored the effects of NeuroD2-E2 and mCherry-Id2 co-expression on the localization of genuine NLS-lacking TCF4 isoform A^−^. Similarly to EGFP-bHLH, the distribution of TCF4-A^−^ was guided by its heterodimerization partner (Figure 6E). Altogether these results demonstrate that TCF4 can be directed to the nucleus by two mechanisms. Firstly, isoforms that bear the NLS independently translocate to the nucleus. Secondly, all TCF4 isoforms can be transported to the nucleus by a piggy-back mechanism through heterodimerization with NLS containing bHLH partners. Additionally, isoforms lacking NLS can be exported from the nucleus by heterodimerization with NES containing partner proteins.

Subsequently, we examined the intracellular distribution of endogenous TCF4. For this we chose to study human hippocampus and cerebellum because of high TCF4 expression levels in these brain regions. We performed immunohistochemical staining of the sections using TCF4 and neuronal marker NeuN or glial marker GFAP specific antibodies, followed by confocal microscopy. As shown in Figure 6F, TCF4 signal was found mainly in the neuronal nuclei but also in the cytoplasm of neurons in different hippocampal regions (CA, dentate gyrus, hilus) and cerebellar granular layer. Of note, the TCF4 antibody additionally marked processes of GFAP-positive cells in tissues and rat primary cultures, but to our knowledge this glial cytoplasmic signal is non-specific as no such staining was observed when tagged TCF4 isoforms were overexpressed in cultured rat glial cells and stained with tag-specific antibodies (data not shown).

### TCF4 isoforms activate transcription differentially

**Figure 7:**
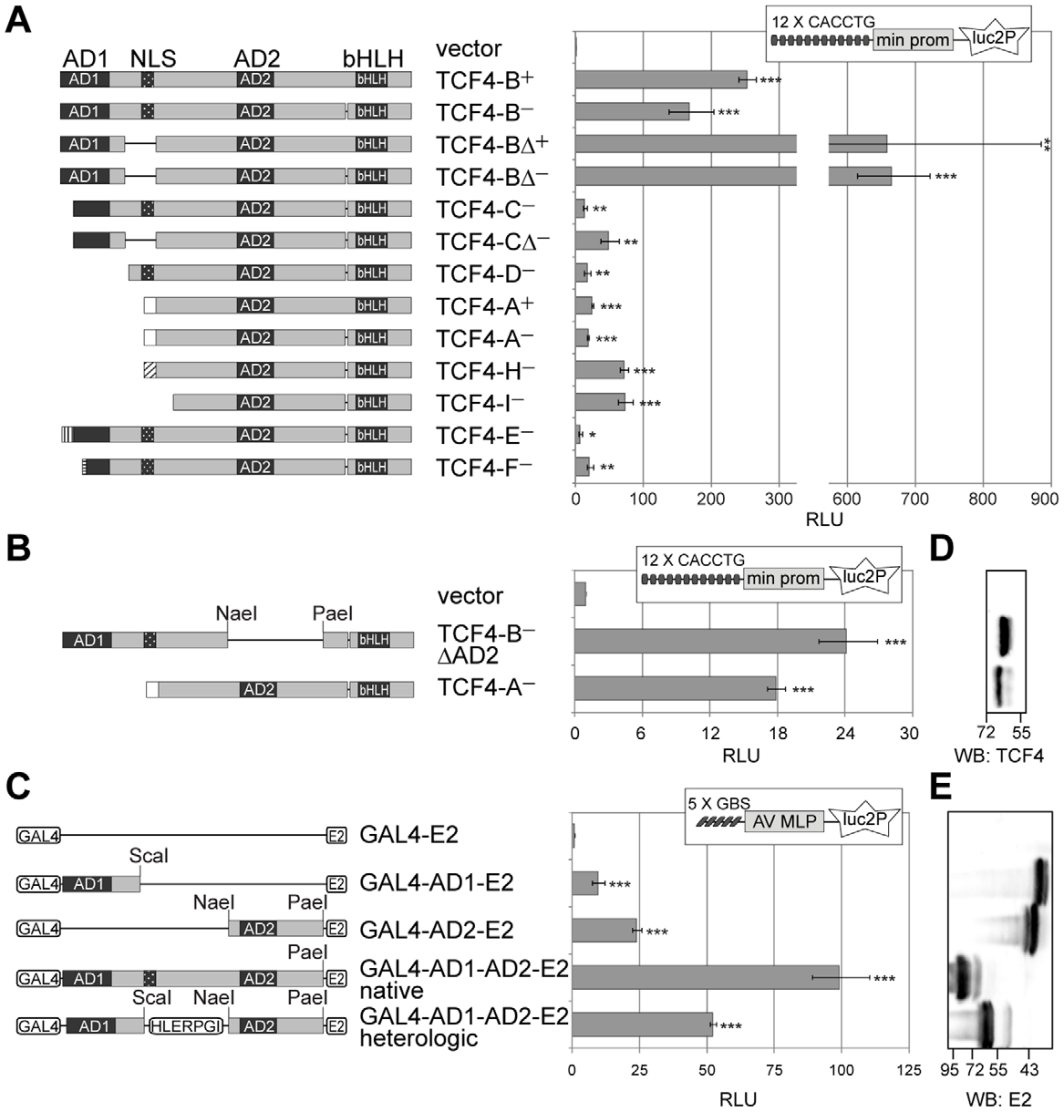
Transcription activation by alternative TCF4 isoforms. (**A**) Reporter assay with HEK293 cells transfected with firefly luciferase construct carrying 12 µE5 E-boxes in front of a minimal (min) promoter along with the indicated TCF4 isoform encoding plasmid or an empty vector. (**B**) Reporter assay with HEK293 cells transfected with luciferase constructs and TCF4-B^−^ΔAD2 or TCF4-A^−^ encoding plasmid or empty vector as indicated. (**C**) Reporter assay with HEK293 cells transfected with firefly luciferase construct carrying 5 GAL4 binding sites (GBS) in front of adenovirus major late promoter (AV MLP) along with the indicated E2-tagged GAL4 fusion proteins. (**A**, **B** and **C**) For normalization *Renilla* luciferase construct with minimal promoter was cotransfected. Luciferase activities were measured and data are presented as fold induced levels above the signals obtained from empty vector transfected cells. Shown are the mean results from at least three independent experiments performed in duplicates, error bars indicate standard deviations. Statistical significance shown with asterisks is relative to the luciferase activity measured from empty vector transfected HEK293 cells (*, p<0.05; **, p<0.01; ***, p<0.001; t-test). RLU, relative luciferase units. Schematic representation of the expressed TCF4 proteins with the locations of restriction enzymes used for the generation of the respective plasmids is shown at the left. (**D**) Western blot analysis of TCF4-B^−^ΔAD2 and TCF4-A^−^ expressed in HEK293 cells. (**E**) Western blot analysis of E2-tagged GAL4 fusion proteins expressed in HEK293 cells. (**D** and **E**) Localization of molecular mass (in kDa) marker bands is indicated at the bottom and the order of samples is as in B and C, respectively.

As a next step in functional characterization of TCF4 isoforms we performed reporter assays with a construct carrying 12 µE5 (CACCTG) E-boxes [1] in front of a minimal promoter controlling the expression of firefly luciferase gene *luc2P* (Figure 7A). The reporter construct was transfected into HEK293 cells together with expression plasmids encoding different TCF4 isoforms and a vector with the minimal promoter in front of *Renilla* luciferase *hRlucP* gene. Compared to empty vector transfected cells the normalized luciferase activity was approximately 250 or 150 times higher in cells expressing TCF4-B^+^ or -B^−^, respectively. More than 650-fold increase was observed with TCF4-B Δ^+^ and -B Δ^−^ isoforms. TCF4-C^−^ activated reporter gene transcription over 10 and TCF4-C Δ^−^ almost 50 times. The increased luciferase activity in Δ isoforms expressing cells compared to the respective full-length isoform expressing cells could reflect higher levels of Δ isoforms in HEK293 cells (Figure 5C). The two isoforms with low expression level, TCF4-E^−^ and -F^−^, activated reporter gene transcription about 7- and 21-fold, correspondingly. Isoforms completely lacking AD1 elevated luciferase activity approximately 20 (TCF4-D^−^, -A^+^ and -A^−^) or 70 times (TCF4-H^−^ and -I^−^). Search for correlations revealed a strong positive relationship between the presence of full-length AD1 and isoform's ability to transactivate in HEK293 cells (point biserial correlation coefficient r = 0.87, p<0.00011). There were no significant correlations between the isoform's capacity to activate transcription and the presence of NLS, presence of only the C-terminal part of AD1 or presence of the extra four amino acids in the + isoforms. These results indicate that, although to a different extent, all TCF4 isoforms analyzed are able to activate transcription controlled by µE5 E-boxes in HEK293 cells.

To study further the individual role of TCF4 transcription activation domains AD1 and AD2, we took two approaches. Firstly, we compared the abilities of artificial TCF4-B^−^ without AD2 (ΔAD2) and native TCF4-A^−^ to regulate E-box controlled transcription. TCF4-B^−^ ΔAD2 contains the AD1 and TCF4-A^−^ the AD2 domain, both have the bHLH domain. Secondly, we used heterologic constructs where AD1 or AD2 is fused with GAL4 DNA binding domain and E2 epitope-tag. We assessed the ability of the GAL4 fusion proteins to activate reporter transcription from pG5luc vector that carries GAL4 binding sites in front of firefly luciferase *luc* gene. As shown in Figures 7B and 7C similar reporter gene activation levels were achieved with TCF4-B^−^ ΔAD2 compared to TCF4-A^−^, and GAL4-AD1-E2 compared to GAL4-AD2-E2 in HEK293 cells. When joined to native bHLH DNA binding domain, 24 or 18 fold upregulation of reporter gene transcription were seen with AD1 or AD2 containing proteins, respectively (Figure 7B) When fused with heterologic GAL4 DNA binding domain, AD1 activated transcription about 10 times and AD2 about 24 times (Figure 7C). We noted that when both activation domains are present in a single protein as is the case for TCF4-B isoforms, the activation of transcription exceeds the additive effect of AD1 and AD2, suggesting that the two domains may act synergistically in HEK293 cells. To test this we studied the transactivation capacity of GAL4 fusion proteins that contain both TCF4 activation domains joined by native or heterologic amino acids. As shown in Figure 7C these proteins activated reporter transcription approximately 100 and 50 times, respectively. In both cases the activation fold exceeded the additive effect of single TCF4 activation domain containing GAL4 fusion proteins. All the compared proteins were expressed at similar levels in HEK293 cells as determined by western blotting with TCF4 or E2-specific antibodies (Figures 7D and 7E). Altogether these data show that the two activation domains are capable of mediating transactivation to a similar extent in HEK293 cells. Additionally, the results indicate that when both activation domains are present in a single protein, the two domains act synergistically in HEK293 cells.

## Discussion

Large-scale human transcriptome analyses have revealed that more than 90% of protein-coding genes undergo alternative splicing and around half have two or more alternative promoters [26], [30]–[32]. Additionally, over-representation of alternative promoters has been attributed to genes involved in development and regulation of transcription [32]. Here, we demonstrate that the human gene for the transcription factor TCF4 is transcribed using 21 mutually exclusive 5′ initial exons, many of which contain more than one transcription start sites and/or splice donor sites. These 5′ exons are located at various positions in the gene interspersed with internal exons 1–9, followed by constitutive internal exons 10–20 and 3′ exon 21. Therefore, *TCF4* transcripts containing different number of internal exons are generated and, according to our data, these are expressed at comparable levels in human tissues and potentially encode for TCF4 protein isoforms with 18 different N-termini. We named the isoforms TCF4-A – TCF4-R in agreement with the previous studies that have described three N-terminally distinct isoforms, i.e. TCF4-A, -B and -D [2], [18]. Additionally, several studies have described TCF4 isoforms differing by the presence or absence of four amino acids (RSRS) N-terminal to the bHLH domain [2], [18], [33], [34], which are denoted here as + and − isoforms, respectively. These isoforms result from alternative splice donor site selection at exon 18 and their mRNAs are present at comparable levels in most human tissues analyzed here. We show that in human *TCF4* gene there is another site of alternative splicing at internal exons 8–9. Transcripts lacking these exons are expressed at low levels in human tissues and code for protein isoforms indicated with Δ in this study.

One by one analysis of alternative 5′ exons containing *TCF4* transcripts' expression and quantitative analysis of overall *TCF4* expression in human tissues corroborated the concept of TCF4 being a ubiquitous transcription factor. Most alternative 5′ exons containing transcripts were broadly expressed. Nevertheless, expression of transcripts containing 5′ exons 1a, 1b, 3a and 5c was detected only in a few tissues including testis and prostate. These exons originate from exonization of different transposable elements (TEs) or are located immediately behind a TE in the human genome. Incorporation of TE-derived sequences into promoters or UTR or coding regions of genes has been documented by many studies [35]–[37]. In accordance with the restricted expression of TE-dependent *TCF4* transcripts, TEs are known to be transcriptionally silenced in most mammalian tissues as a defense mechanism against potentially deleterious effects of their activity [38], [39]. TE-dependent *TCF4* transcripts code for 5 unique TCF4 protein isoforms with long N-termini (TCF4-J – TCF4-N). Since it was not possible to distinguish all TCF4 isoforms from each other by SDS-PAGE fractionation, we were not able to conclusively determine which protein isoforms are expressed in human tissues; nevertheless, we detected three prominent TCF4 forms of different molecular weight. Notably, high Mw TCF4 protein was relatively more abundant in testis, medium Mw TCF4 in lung and low Mw TCF4 was the major form in the brain tissue, insinuating that the ratio of different TCF4 isoforms with distinct N-termini is varied between human tissues.

Our report reveals that although broadly expressed, *TCF4* transcript levels differ greatly between tissues. The highest levels are present in fetal brain, but expression remains elevated also in adult brain where we detected *TCF4* mRNA and protein in neurons. These findings are consistent with earlier studies that have demonstrated TCF4 expression in human or rodent nervous system [18], [40]–[44] and further support the important role for TCF4 in the development and functioning of the nervous system as exemplified by involvement of TCF4 in the pontine nucleus development in mice, association of *TCF4* with schizophrenia and identification of *TCF4* haploinsufficiency as the cause of Pitt-Hopkins mental retardation syndrome [17], [21]–[24]. Interestingly, translocation that results in a *TCF4* allele without at least 6 upper 5′ exons has been described in a patient with mild mental retardation [45]. Possibly, in this patient, initiation of *TCF4* transcription at downstream 5′ exons still takes place, explaining the less severe phenotype than in classical Pitt-Hopkins syndrome patients. Nevertheless, it seems that for production of sufficient amounts of TCF4 protein and normal development, the presence of all transcription initiation sites is critical. Based on experiments with *HEB* and *E2A* knockout mice, it has been suggested that the overall E-protein dosage is more crucial in the development of nervous system than family member identity [46]. However, since even slight disturbances in *TCF4* expression in human cause a neurodevelopmental disorder and *TCF4* knockout mice display disrupted pontine nucleus development [17], it is apparent that other E-proteins are not able to compensate for the loss of TCF4 in all aspects of the nervous system development. Additionally, transgenic mice with mild overexpression of *TCF4* in forebrain display deficits in contextual and cued fear conditioning and sensorimotor gating [47], indicating that precise regulation of *TCF4* expression is crucial for correct brain function and deviations in either way result in cognitive disturbances. All this substantiates the complex structure and the high number of 5′ exons in *TCF4* gene, as these probably enable proper amounts of TCF4 protein to be synthesized. It is noteworthy that according to the *TCF4* gene structure described here, the SNP rs9960767 associated with susceptibility to schizophrenia [21] is located in *TCF4* intronic sequence between 5′ exons 5b and 5c. However, whether and how this marker is coupled to dysregulation of *TCF4* expression remains to be elucidated.

The variety of TCF4 isoforms raises a question – are they produced only for sufficient amount of TCF4 proteins or are the functions of different isoforms divergent? In the present study we have shown that the alternative TCF4 isoforms differ in two aspects: first, their intracellular distribution is differentially regulated depending on the presence or absence of a nuclear localization signal; second, their ability to activate E-box controlled transcription is varied depending mainly on whether they contain one or two functional transcription activation domains. We discovered in the region coded by exons 8–9, upstream of AD2, a bipartite NLS that is responsible for nuclear localization of TCF4 isoforms with longer N-termini. This NLS is conserved among E-proteins and mutating the second half of the NLS has been demonstrated to reduce nuclear import of E2A [48]. TCF4 isoforms with shorter N-termini (TCF4-A, -H and -I) and Δ isoforms do not contain the NLS. Our analysis shows that these proteins are transported to the nucleus via heterodimerization with NLS-bearing partners and directed to the cytoplasm by forming dimers with NES-containing proteins. As substantial amount of NLS-lacking TCF4 isoforms overexpressed in HEK293 cells is localized to the nuclei, there must be abundance of NLS-containing heterodimerization partners expressed endogenously. This is not surprising since TCF4 is able to form dimers with a variety of class B bHLH factors [3]. In addition, we noticed that NLS-bearing TCF4-B isoform was not able to mediate nuclear redirection of NLS-lacking TCF4-A isoform (data not shown), suggesting that TCF4 homodimers are not efficiently formed in HEK293 cells.

E-proteins function as transcription activators or repressors [49]. In our reporter assays all TCF4 isoforms were able to activate transcription from a promoter containing µE5 E-boxes in HEK293 cells whereas TCF4-B isoforms were more potent transactivators than other isoforms. All the studied isoforms contain the AD2 domain, but only TCF4-B isoforms contain full-length AD1, including exon 3 encoded LDFS motif that is known to be required for AD1 mediated transactivation by recruiting histone acetyltransferases [50], [51]. This motif is present also in some of the TE-dependent isoforms (TCF4-J, -K, and -L) that were not included in our reporter assays. We suggest that the AD1 acts synergistically with the AD2, since the additive effect of individual capacities of these domains to activate transcription is surpassed by proteins that contain both TCF4 activation domains. Similar synergism between two activation domains has been described in bHLH-zipper protein TFE3 and POU homeodomain protein Oct-2 [52], [53]. In contrast to a study on *FGF-1* promoter that proposed differential roles for TCF4 + and − isoforms [33], we saw no differences between the + and − isoforms in ability to activate transcription from µE5 E-boxes controlled promoter. However, we noticed higher reporter gene transcription in the presence of Δ isoforms than the respective full-length isoforms, probably due to higher expression levels of Δ isoforms compared to full-length isoforms. This indicates that NLS coding region absent in Δ isoforms could affect the stability of TCF4 proteins. In sum, we show that differences in the functioning of alternative TCF4 isoforms do exist. In the light of the knowledge that an isoform of HEB, homologous to TCF4-A, is specifically required for the generation of T-cell precursors *in vivo* and this function cannot be carried out by the HEB isoform homologous to TCF4–B [54], it would be of importance to study the distinct functions of TCF4 isoforms by rescue experiments with different TCF4 isoforms in *TCF4* knockout background.

## Materials and Methods

### Ethics statement

All experiments with human postmortem tissues were approved by the ethics committee of medical studies at National Institute for Health Development of Estonia (Permit Number: 402). The protocols involving animals were approved by the ethics committee of animal experiments at Ministry of Agriculture of Estonia (Permit Number: 45).

### Bioinformatic analyses

Human *TCF4* gene structure and mRNAs were identified by analyzing genomic, mRNA and expressed sequence tag (EST) databases using tools available at http://www.ncbi.nlm.nih.gov and http://genome.ucsc.edu. The locations of transposable elements were determined by RepeatMasker track in UCSC Genome browser. Nuclear localization signal (NLS) was predicted using software at http://wolfpsort.org. Sequence alignments were prepared with tools available at http://www.ebi.ac.uk/Tools. The nucleotide sequences have been deposited in the EMBL Nucleotide Sequence Database under Accession Numbers FR748202-FR748223.

### Constructs

Standard methods of recombinant DNA technology were used for generation of all constructs. Full-length coding regions of TCF4 isoforms were PCR-amplified from human brain cDNA and cloned into pcDNA3.1 (Invitrogen). Both, constructs coding for native TCF4 and C-terminally V5/His-tagged TCF4 isoforms, were created. TCF4-B^−^ amino acid sequence is used as a reference for the description of following TCF4 constructs. pEGFP-C1-C3 (Clontech) were used for generation of pEGFP-NLS, pEGFP-bHLH, pEGFP-bHLH-N and pEGFP-bHLH-C that code for EGFP fusion proteins containing TCF4 amino acids P156-P178, I541-M667, I541-K585 and E586-M667, respectively. Mutagenesis of TCF4 NLS was performed using complementary primers against the target sequence containing the respective mutation using Phusion High-Fidelity DNA Polymerase (Finnzymes). pcDNA-TCF4B^−^ΔAD2 codes for TCF4-B^−^ without amino acids G316-M497. GAL4 DNA-binding domain was obtained from pBind vector (Promega) and inserted into pQM-CMV-E2-C vector (Icosagen). TCF4 activation domains AD1 (M1-Y148), AD2 (G316-G496), AD1–AD2 (M1-G469) and AD1 plus AD2 with heterologic linker (M1-Y148, HLERPGI, G316–G496) were cloned in-frame between GAL4 DNA-binding domain and E2-tag. Full-length coding regions of NeuroD2 and Id2 were PCR-amplified from mouse brain cDNA and inserted into pQM-CMV-E2-C (Icosagen) in front of E2 tag and pmCherry-C (Clontech) behind mCherry sequence, respectively. For E-box reporter vector pGL4.29[luc2P/12 µE5/Hygro], the CRE binding-site in pGL4.29[luc2P/CRE/Hygro] (Promega) was replaced with 12 µE5 E-boxes by tandem insertion of annealed oligonucleotides. For pGL4[hRlucP/min/Hygro], the 12 µE5 E-boxes were removed from pGL4.29[luc2P/12 µE5/Hygro] and firefly luciferase encoding *luc2P* was replaced with *Renilla* luciferase encoding *hRlucP* gene from pGL4.83[hRlucP/Puro] (Promega). For pBluescript-TCF4(3F-11R) and pSC-A-TCF4(10F-16R) used for cRNA probe synthesis, PCR amplified TCF4 cDNA fragments spanning exons 3–11 and 10–16 were ligated into EcoRV linearized pBluescriptKS+ vector or cloned into pSC-A vector (Stratagene), respectively. Sequences of all oligonucleotides used are listed in Supporting Table S2.

### Ribonuclease protection assay

EcoRI linearized pBluescript-TCF4(3F-11R) was subjected to *in vitro* transcription using MAXIscript Kit and T3 polymerase (Ambion). The concentration of limiting nucleotide (UTP) was 10 µM of which 1/6 was [α-^32^P]UTP (specific activity 3000 Ci/mmol; Hartmann analytics). 10 µg of total RNA and 2.5×10^5^ CPM of radiolabeled probe were used for hybridization and the assay was performed with the RPA III Kit (Ambion) as suggested by the manufacturer. The protected fragments were separated in 5% acrylamid urea gel, visualized by autoradiography and quantified with ImageQuant T4 software (Amersham Biosciences).

### RNA isolation and RT-PCR

Total RNAs from postmortem adult human brain regions and muscle were purified using RNAwiz reagent (Ambion) and treated with TURBO DNase (Ambion). Other human tissue RNAs were obtained from Clontech. First-strand cDNAs were synthesized from 5 µg of total RNA with Superscript III reverse transcriptase (Invitrogen) with oligo(dT) primers according to manufacturer's recommendations. PCR amplification was performed using HotFire polymerase (Solis Biodyne). For quantitative PCR, LightCycler 2.0 engine (Roche), qPCR Core kit for SYBR R Green I No ROX (Eurogentec) and polycarbonate qPCR capillaries (Bioron) were used. The reactions were carried out in a volume of 10 µl containing 1/80 of reverse transcription reaction as a template. In control, PCR with primers specific for the ubiquitously expressed hypoxanthine-guanine phosphoribosyltransferase (HPRT), succinate dehydrogenase complex subunit A (SDHA), ubiquitin C (UBC), hydroxymethylbilane synthase (HMBS) and glyceraldehyde-3-phosphate dehydrogenase (GAPDH) were performed. For normalization of quantitative PCR data the geometric mean of four selected housekeeping genes (SDHA, UBC, HMBS and GAPDH) with high expression stability (M<1.5) was calculated using geNorm software [55]. For quantification of *TCF4* mRNA expression, three different primer pairs detecting all *TCF4* transcripts were designed (products spanning exons 10–11, 17–18 and 19–20). The results obtained with each primer pair were normalized with the geNorm calculated factor, log-transformed and standardized as described [56]. Means and standard deviations (SD) were calculated and the data were back-transformed to the original scale for graphical representation. The bars represent geometric means and error bars represent upper and lower limits back-transformed as mean+SD and mean–SD, respectively. All products from the RT-PCR reactions were verified by sequencing. The primers together with the used annealing temperatures, cycle numbers and product sizes are listed in Supporting Table S2. When indicated PCR amplified DNA was diluted three times and subjected to restriction with BglII (Fermentas).

### *In situ* hybridization

cRNA probes were synthesized from BamHI linearized pSC-A-TCF4(10F-16R) with MAXIScript *in vitro* Transcription Kit and T3 polymerase (Ambion), using [α-^35^S]UTP (Amersham Biosciences) for labeling. Serial coronal sections (16 µm) from fresh-frozen adult male human hippocampus and cerebellum were subjected to *in situ* hybridization following the protocol described earlier [57]. Emulsion-dipped sections were developed after 3 weeks using D-19 developer (Eastman Kodak), fixed (sodium fixer; Kodak), and counterstained with hematoxylin (Shandon).

### Cell culture and transfection

Human embryonic kidney HEK293 cells and mouse neuroblastoma Neuro2A cells were grown in MEM (Minimum Essential Medium Eagle; PAA) or DMEM (Dulbecco's modified Eagle's medium; PAA), respectively, supplemented with 10% fetal bovine serum (PAA), 100 U/ml penicillin (PAA) and 0.1 mg/ml streptomycin (PAA) at 37°C in 5% CO_2_. For transfection of DNA constructs 0.375 µg DNA and 0.75 µl of LipoD293 reagent (SignaGen) were used per well of a 48-well plate or scaled up accordingly. In case of cotransfections, equal amounts of all plasmids were used. For transfection of siRNAs 24 pmol siRNA and 4 µl of Lipofectamine RNAiMAX (Invitrogen) were used per well of a 6-well plate. siRNAs were ordered from Ambion and their sequences are brought in Supporting Table S2.

### Protein extracts and Western blotting

*In vitro* translation was performed using TnT Quick Coupled Transcription/Translation System (Promega) according to manufacturer's instructions. Cell and tissue extracts were prepared in RIPA buffer (50 mM Tris HCl pH 8.0, 150 mM NaCl, 1% NP-40, 0.5% Na-DOC, 0.1% SDS, 1 mM DTT, 1 mM PMSF, protease inhibitors cocktail Complete (Roche)). Protein concentrations were determined using BCA assay (Pierce). Equal amounts of proteins were separated in 8% SDS-PAGE and transferred to PVDF membrane (Biorad). For western blotting the antibodies were diluted in 2% skim milk and 0.1% Tween 20 in PBS as following: rabbit polyclone anti TCF4/ITF2 (CeMines) 1∶1000, mouse monoclone anti E2 (Icosagen; 5E11) 1∶5000, mouse monoclone anti tubulin β (Developmental Studies Hybridoma Bank) 20 ng/ml, HRP-conjugated goat anti mouse/rabbit IgG (Pierce) 1∶5000. Chemiluminescent signal was detected using SuperSignal West Femto Chemiluminescent Substrate (Pierce).

### Cyto- and histochemical immunostaining

For cytochemistry, cells were grown on poly-L-lysine (Sigma) coated coverslips and fixed with 4% paraformaldehyde for 15 minutes, treated with 50 mM NH_4_Cl in PBS for 10 minutes and permeabilized in 0.5% Triton X-100 in PBS for 15 minutes. Cells were blocked with 2% bovine serum albumin (BSA) in PBS. The reactions with primary and secondary antibodies were carried out in 0.2% BSA and 0.1% Tween 20 in PBS at room temperature. For histochemistry, 16 µm coronal sections from fresh-frozen adult male human hippocampus and cerebellum were fixed with 4% paraformaldehyde for 30 minutes and blocked in 0.25% Triton X-100, 0.5% Tween 20, 3% goat serum in PBS. Primary antibody reactions were carried out in blocking buffer overnight at 4°C. The antibodies were diluted as following: rabbit polyclone anti TCF4/ITF2 (CeMines) 1∶200, mouse monoclone anti V5 (Invitrogen) 1∶500, mouse monoclone anti E2 (Icosagen, 5E11) 1∶500, mouse monoclone anti NeuN 1∶100 (Chemicon), mouse monoclone anti GFAP 1∶800 (Chemicon), Alexa 488 or Alexa 568 conjugated goat anti mouse/rabbit IgG (Molecular Probes) 1∶2000. The samples were mounted in ProLong Gold antifade reagent with DAPI (Molecular Probes) and analyzed by confocal microscopy (LSM Duo, Zeiss).

### Luciferase assays

Cells on 48-well plates were lysed 24 hours post-transfection in 50 µl Passive Lysis Buffer (Promega). Dual-Glo Luciferase assay (Promega) was performed following manufacturer's instructions and luminescence was measured with GENios pro (Tecan) plate reader. For data analysis background signals from untransfected cells were subtracted and firefly luciferase signal values were normalized to *Renilla* luciferase signals. The obtained data were log-transformed, autoscaled, means and standard deviations (SD) were calculated and t-tests for analyses of statistical significance were performed. For graphical representation, the data were back-transformed to the original scale. Error bars represent upper and lower limits back-transformed as mean+SD and mean–SD, respectively. Correlation analysis was performed with tools at http://faculty.vassar.edu/lowry/pbcorr.html.

## Supporting Information

Figure S1 Alignment of TCF4 isoforms. Amino acids different from the consensus are in blue. Localization of functional domains is indicated with lines above the sequence. Amino acid(s) in parentheses are absent from (1) isoforms coded by transcripts spliced at acceptor II of exon 8, (2) Δ isoforms coded by transcripts without exons 8–9, (3) isoforms coded by transcripts spliced at acceptor II of exon 15, (4) − isoforms (as opposed to + isoforms) coded by transcripts spliced at donor I of exon 18. AD, activation domain; NLS, nuclear localization signal; bHLH, basic helix-loop-helix domain.
(PDF)

Figure S2 Multiple alignment of *Homo sapiens* (h) genomic DNA regions containing *TCF4* 5′ exons and internal exons 1–2 with the respective regions in *Pan troglodytes* (c), *Mus musculus* (m), *Rattus norvegicus* (r) and/or *Macaca mulatta* genomes (rh). The exons' names are given above each aligned region. The nucleotides in the aligned regions are numbered according to human genome assembly March 2006 NCBI36/hg18, chimp genome assembly March 2006 CGSC 2.1/panTro2, mouse genome assembly July 2007 NCBI37/mm9, rat genome assembly November 2004 Baylor 3.4/rn4 and rhesus genome assembly January 2006 MGSC merged 1.0/rheMac2. Alignments were produced with ClustalW and the percentages of identity were calculated between human TCF4 5′ exon sequence and the respective mouse sequence using Needleman-Wunsch global alignment. The exon sequences are in bold case, internal exons are in blue and sequences of primers used for expression analysis are underlined. Dotted blue lines above the sequences indicate transposable elements. Arabic numerals above exonic sequences indicate the number of human ESTs starting at the respective position and obtained from oligo-cap, cap-trapping and SMART libraries available in public databases through UCSC genome browser as of 3rd of November 2010. Possible in-frame translation start codons are shaded in gray. For each in-frame ATG codon NetStart translation start score is shown above the start codon. When needed the locations of alternative splice donor sites are indicated with blue roman numerals above the first intronic nucleotides (or above the first internal exon nucleotides in case of exon 7b-II).
(PDF)

Table S1 Accession numbers of representative mRNA or EST sequences for alternative *TCF4* transcripts and complete coding sequences of TCF4 isoforms cloned in full-length in this study.
(PDF)

Table S2 List of oligonucleotides used in this study.
(PDF)
