# Supplementary material for: Functional Diversity of Human Basic Helix-Loop-Helix Transcription Factor TCF4 Isoforms Generated by Alternative 5′ Exon Usage and Splicing
Source: PLoS One. 2011 Jul 15;6(7):e22138. doi: 10.1371/journal.pone.0022138 (PMC3137626; doi:10.1371/journal.pone.0022138)
Supplement: Figure S1 — Alignment of TCF4 isoforms. Amino acids different from the consensus are in blue. Localization of functional domains is indicated with lines above the sequence. Amino acid(s) in parentheses are absent from (1) isoforms coded by transcripts spliced at acceptor II of exon 8, (2) Δ isoforms coded by transcripts without exons 8–9, (3) isoforms coded by transcripts spliced at acceptor II of exon 15, (4) − isoforms (as opposed to + isoforms) coded by transcripts spliced at donor I of exon 18. AD, activation domain; NLS, nuclear localization signal; bHLH, basic helix-loop-helix domain. (PDF) [file pone.0022138.s001.pdf]

```
TCF4-K   MVTLVQHSFSNSAHSLDVYSITFLVDWHLCGQRNNTMFSKRLEKIAQVPLLFPFIFIILTNYSKMEGAVESQPSFFKTSQDIVTCTWVENCYSSFSRRPLEMESHSVIQTGVQWHHLSSLQPP
TCF4-J   MVTLVQHSFSNSAHSLDVYSITFLVDWHLCGQRNNTMFSKRLEKIAQVPLLFPFIFIILTNYSKMEGAVESQPSFFKTSQDIVTCTWVENCYSSFSRRPLEQMFCKHQSKNIISWTGMVAHTC
TCF4-L                                                                                                        MESHSVIQTGVQWHHLSSLQPP

                                    AD1
TCF4-K   PPRFKLSSCLSLLSTRDRRLCDFAKMHHQQRMAALGTDKELSDLLDFSAMFSPPVSSGKNGPTSLASGHFTGSNVEDRSSSGSWGNGGHPSPSRNYGDGTPYDHMTSRDLGSHDNLSPPFVNS
TCF4-J   NPSTLGGQG----------LCDFAKMHHQQRMAALGTDKELSDLLDFSAMFSPPVSSGKNGPTSLASGHFTGSNVEDRSSSGSWGNGGHPSPSRNYGDGTPYDHMTSRDLGSHDNLSPPFVNS
TCF4-L   PPRFKLSSCLSLLSTRDRRLCDFAKMHHQQRMAALGTDKELSDLLDFSAMFSPPVSSGKNGPTSLASGHFTGSNVEDRSSSGSWGNGGHPSPSRNYGDGTPYDHMTSRDLGSHDNLSPPFVNS
TCF4-B                             MHHQQRMAALGTDKELSDLLDFSAMFSPPVSSGKNGPTSLASGHFTGSNVEDRSSSGSWGNGGHPSPSRNYGDGTPYDHMTSRDLGSHDNLSPPFVNS
TCF4-E                               MQRAKTELFRLQIVTDDLRKNEMFSPPVSSGKNGPTSLASGHFTGSNVEDRSSSGSWGNGGHPSPSRNYGDGTPYDHMTSRDLGSHDNLSPPFVNS
TCF4-M                         MRASKGSEGCQHAVTSQLVTGAGGDQTEMFSPPVSSGKNGPTSLASGHFTGSNVEDRSSSGSWGNGGHPSPSRNYGDGTPYDHMTSRDLGSHDNLSPPFVNS
TCF4-O                                         MLEREGVKERVRMFSPPVSSGKNGPTSLASGHFTGSNVEDRSSSGSWGNGGHPSPSRNYGDGTPYDHMTSRDLGSHDNLSPPFVNS
TCF4-P                                          MEMDIATFMAKMFSPPVSSGKNGPTSLASGHFTGSNVEDRSSSGSWGNGGHPSPSRNYGDGTPYDHMTSRDLGSHDNLSPPFVNS
TCF4-C                                                     MFSPPVSSGKNGPTSLASGHFTGSNVEDRSSSGSWGNGGHPSPSRNYGDGTPYDHMTSRDLGSHDNLSPPFVNS
TCF4-N                                                         MLKMFFRISQVAGSSWYSKDVEDRSSSGSWGNGGHPSPSRNYGDGTPYDHMTSRDLGSHDNLSPPFVNS
TCF4-F                                                                      MEEDSRDVEDRSSSGSWGNGGHPSPSRNYGDGTPYDHMTSRDLGSHDNLSPPFVNS
TCF4-R                                                                       MSIFENVEDRSSSGSWGNGGHPSPSRNYGDGTPYDHMTSRDLGSHDNLSPPFVNS
TCF4-Q                                                                                                           MTSRDLGSHDNLSPPFVNS
TCF4-G                                                                                                    MKDIFFQFIIARVRKCYSLSCLHTLPVV

                                         1                                   NLS                    2
TCF4     RIQSKTERGSYSSYGRESNLQGCHQ(Q)SLLGGDMDMGNPGTLSPTKPGSQYYQYSSNNPRRRPLHSSAMEVQTKKVRKVPPGLPSS(V)YAPSASTADYNRDSPGYPSSKPATSTFPSSFFMQDGH
TCF4-G   PTLRKTERGSYSSYGRESNLQGCHQ(Q)SLLGGDMDMGNPGTLSPTKPGSQYYQYSSNNPRRRPLHSSAMEVQTKKVRKVPPGLPSS(V)YAPSASTADYNRDSPGYPSSKPATSTFPSSFFMQDGH
TCF4-D                                   MDMGNPGTLSPTKPGSQYYQYSSNNPRRRPLHSSAMEVQTKKVRKVPPGLPSSVYAPSASTADYNRDSPGYPSSKPATSTFPSSFFMQDGH
TCF4-A                                                                MYCAYTIPGMGGNSLMYYYNGKAVYAPSASTADYNRDSPGYPSSKPATSTFPSSFFMQDGH
TCF4-H                                                                MKFKQCRCSDTGLCCLDHEGKAEVYAPSASTADYNRDSPGYPSSKPATSTFPSSFFMQDGH
TCF4-I                                                                                                                       MQDGH

                                                                                                                           AD2
TCF4     HSSDPWSSSSGMNQPGYAGMLGNSSHIPQSSSYCSLHPHERLSYPSHSSADINSSLPPMSTFHRSGTNHYSTSSCTPPANGTDSIMANRGSGAAGSSQTGDALGKALASIYSPDHTNNSFSSN

                      3
TCF4     PSTPVGSPPSLS(A)GTAVWSRNGGQASSSPNYEGPLHSLQSRIEDRLERLDDAIHVLRNHAVGPSTAMPGGHGDMHGIIGPSHNGAMGGLGSGYGTGLLSANRHSLMVGTHREDGVALRGSHSL

                                                                                       4                    bHLH
TCF4     LPNQVPVPQLPVQSATSPDLNPPQDPYRGMPPGLQGQSVSSGSSEIKSDDEGDENLQDTKSSEDKKLDDDKKDIKSIT(RSRS)SNNDDEDLTPEQKAEREKERRMANNARERLRVRDINEAFKE

TCF4     LGRMVQLHLKSDKPQTKLLILHQAVAVILSLEQQVRERNLNPKAACLKRREEEKVSSEPPPLSLAGPHPGMGDASNHMGQM
```

Supporting Figure S1. Alignment of TCF4 isoforms. Amino acids different from the consensus are in blue, localization of functional domains is indicated with lines above the sequence. Amino acid(s) in parentheses are absent from (1) isoforms coded by transcripts spliced at acceptor II of exon 8, (2) Δ isoforms coded by transcripts without exons 8-9, (3) isoforms coded by transcripts spliced at acceptor II of exon 15, (4) – isoforms (as opposed to + isoforms) coded by transcripts spliced at donor I of exon 18. AD, activation domain; NLS, nuclear localization signal; bHLH, basic helix-loop-helix domain.
