# Supplementary material for: Functional Diversity of Human Basic Helix-Loop-Helix Transcription Factor TCF4 Isoforms Generated by Alternative 5′ Exon Usage and Splicing
Source: PLoS One. 2011 Jul 15;6(7):e22138. doi: 10.1371/journal.pone.0022138 (PMC3137626; doi:10.1371/journal.pone.0022138)
Supplement: Figure S2 — Multiple alignment of Homo sapiens (h) genomic DNA regions containing TCF4 5′ exons and internal exons 1–2 with the respective regions in Pan troglodytes (c), Mus musculus (m), Rattus norvegicus (r) and/or Macaca mulatta genomes (rh). The exons' names are given above each aligned region. The nucleotides in the aligned regions are numbered according to human genome assembly March 2006 NCBI36/hg18, chimp genome assembly March 2006 CGSC 2.1/panTro2, mouse genome assembly July 2007 NCBI37/mm9, rat genome assembly November 2004 Baylor 3.4/rn4 and rhesus genome assembly January 2006 MGSC merged 1.0/rheMac2. Alignments were produced with ClustalW and the percentages of identity were calculated between human TCF4 5′ exon sequence and the respective mouse sequence using Needleman-Wunsch global alignment. The exon sequences are in bold case, internal exons are in blue and sequences of primers used for expression analysis are underlined. Dotted blue lines above the sequences indicate transposable elements. Arabic numerals above exonic sequences indicate the number of human ESTs starting at the respective position and obtained from oligo-cap, cap-trapping and SMART libraries available in public databases through UCSC genome browser as of 3rd of November 2010. Possible in-frame translation start codons are shaded in gray. For each in-frame ATG codon NetStart translation start score is shown above the start codon. When needed the locations of alternative splice donor sites are indicated with blue roman numerals above the first intronic nucleotides (or above the first internal exon nucleotides in case of exon 7b-II). (PDF) [file pone.0022138.s002.pdf]

Supporting Figure S2. Multiple alignment of *Homo sapiens* (h) genomic DNA regions containing *TCF4* 5' exons and internal exons 1-2 with the respective regions in *Pan troglodytes* (c), *Mus musculus* (m), *Rattus norvegicus* (r) and/or *Macaca mulatta* genomes (rh).

- The exons' names are given above each aligned region.
- The nucleotides in the aligned regions are numbered according to human genome assembly March 2006 NCBI36/hg18, chimp genome assembly March 2006 CGSC 2.1/panTro2, mouse genome assembly July 2007 NCBI37/mm9, rat genome assembly November 2004 Baylor 3.4/rn4 and rhesus genome assembly January 2006 MGSC merged 1.0 / rheMac2.
- The multiple alignments were produced with ClustalW program at http://www.ebi.ac.uk/Tools/clustalw2/index.html
- The percentages of identity were calculated between human TCF4 5' exon sequence and the respective mouse sequence using Needleman-Wunsch global alignment algorithm at http://www.ebi.ac.uk/Tools/emboss/align/.
- The **exon** sequences are in bold case.
- The **internal exons** are in blue.
- The sequences of primers used for expression analysis are underlined.
- Dotted blue lines above the sequences indicate the transposable elements as determined by RepeatMasker track in UCSC genome browser.
- The arabic numerals above exonic sequences indicate the number of human ESTs starting at the respective position and obtained from oligo-cap, cap-trapping and SMART libraries available in public databases through UCSC genome browser as of 3rd of November 2010.
- Possible in-frame translation start codons are shaded in gray. For each in-frame ATG codon translation start score calculated at http://www.cbs.dtu.dk/services/NetStart is shown above the start codon.
- When needed the locations of alternative splice donor sites are indicated with blue roman numerals (**I**, **II**) above the first intronic nucleotides (or above the first internal exon nucleotides in case of exon 7b-II).

TCF4 exon 1a

Aligned regions

```
h        chr18:51482817-51483103
c        chr18:52061786-52062072
rh       chr18:48945911-48946209
```

note: exon 1a is located in ERV1 family LTR repeats containing region that is not conserved in mouse and rat; the position of both LTR12c and MER51a transposons is conserved in chimp, whereas only MER51a is present at this location in the rhesus macaque genome.

```
h               CAGGCTGAGG--GAGCCGGCTCCCGCCTCGGCCATCCCAGGAAGGGGCTCCCACAGTGCC
c               CAGGCTGAGG--GAGCCGGCTCCCGCCTCGGCCATCCCAGGAAGGGGCTCCCACAGTGCC
rh              CAGTCTGTGGTCAGTCTGATTAGTTGTTGGAGGGGGCCAATCAGAGGCTGAAACAAAGTT
                *** *** **     * *  *      * *      ***   ** ****   ***  *

h               GCGGCG----GGCTGAAGGGCTT--CTCAAGCGCCGCCAGAATTGGCA---CGGACGCCG
c               GCGGCG----GGCTGAAGGGCTT--CTCAAGCGCCGCCAGAATTGGCA---CGGACGCCG
rh              ACAAAGTTATACCCTATGCAATCAACTGATTGGTTGTGGAAAGTAACCAATCAGAGGCTG
                 *   *      *  * *   *   ** *   *  *    ** *  *    * ** ** *

                      0.626                                       ← LTR12c
h               AGGAGGCATGAGAGCGAGCAAGGGCTCTGAGGGCTGCCAGCATGCTGTCACCT--CTCAA
c               AGGAGGCATTAGAGCGAGCAAGGGCTCTGAGGACTGCCAGCATGCTGTCACCT--CTCAA
rh              AAGTGAAGTTACAAAGTTATACTCCTATAAAAATGAAGACTTGGCCCACAACCAGCCTGA
                * * *   * * *  *    *   ** * *        *    **   ** *   *   *

                             MER51A ←
h               TTGGTTACAGGAGCAGGAGGGGACCAAACAGAGGTACTTTCAATTTTTCATCTGCCTCAC
c               TTGGTTACAGGAGCAGGAGGGGACCAAACAGAGGTACTTTCAATTTTTCATCTGCCTCAC
rh              TTGGTTACAGGAGCAGGAGGGGAAAAAACAGAGGTACTTTCAAGTTTTCATCTGCTGCAC
                ***********************  ****************** ***********  ***

h               AGAAAAGGGGGAGGTTTCAAAGGAAGTAGCCTCTGGTCCTTTTGTTACGTGGGCTTGGAA
c               AGAAAAGGGGGAGGTTTCAAAGGAAGTAGCCTCTGGTCCTTTTGTTACGTGGGCTTGGAA
rh              AGAAAAGGGGGAGGTT-CAAAGGGAATAGCTTCTGGTCCTTCTGTTACATGGGCTTGGGA
                **************** ****** * **** ********** ****** ********* *
```

TCF4 exon 1b

```
h        chr18:51453879-51454330
c        chr18:52029997-52030448
m        chr18:69475444-69476010
r        chr18:65989132-65989715
```

exon 1b sequence identity between h and m: 49%
note: exon 1b contains DNA transposon (MER5b) element.

```
h               ATTAATTCTCTTGGCAAGAATCTTTCTCTTATCTTGTTTGTTTACAACAATGCCAACAGC
c               ATTAATTCTCTTGGCAAGAATCTTTCTCTTATCTTGTTTGTTTACAACAATGCCAACAGC
m               GCTAACTCTCTTGCTAACAATCTGTCCCCAAACCTGACTGTTTTTGACAA-GCTACCAAA
r               ACTAAGTCTTTTGCTACCAATCTGTTCCCAAACCTGACT--TTATAACAA-GCTATCAAA
                  *** *** ***  *  ***** *  *  * * **  *  **   **** ** * **

                              1                    0.635
h               ATGCTGGGTTACACAGTAGACTCTTCCAGCTTTGCCATGGTAACACTTGTGCA----GCA
c               ATGCTGGGTTACACAGTAGACTCTTCCAGCTTTGCCATGGTAACACTTGTGCA----GCA
m               ATGCTAGGTTGTATC---------------TTCACAATGGTTGTGGTTGTGAAAGAAGCC
r               ATGCTAGCCTGTATCGTAGACTCTTTGAACTTCACGGTGGTTATAGACGCAAC----GCC
                ***** *  *  *                 **  *  ****       *        **

                             1              3  1   1 1
h               TTCCTTTTCGAACAGTGCCCATTCTCTTGATGTCTACAGTATCACCTTTCTTGTAGATTG
c               TTCCTTTTCGAACAGTGCCCATTCTCTTGATGTCTACAGTATCACCTTTCTTGTAGATTG
m               TTCCTATTTGAAAAGTGCTCATTTCTTTGCTG-CTATAGGATTGTCTTTGTTGGAGAGCT
r               TTCCTATTTGAAAACTGCCCACTTCTTTGCTGGCCATAGGACTGCTTTTGTTGGAGAGCT
                ***** ** *** * *** ** *   *** ** * * ** *     *** *** ***

                                           0.676 1
h               GCACTTATGCGGCCAAAGGAACAACACCATGTTTTCTAAAAGGCTGGAGAAAATAGCACA
c               GCACTTATGTGGCCAAAGGAACAACATCATGTTTTCTAAAAGGCTGGAGAAAATACCACA
m               GTATGTGTAGAGCCGTTGGAATGAGTCCATGGTGTTTGACAGGCCAAA-AGAACATTATT
r               GTATACATAGAGCTAATGGAATGAGCCCATGATTTTTGATAGGCCAG------CATTATT
                * *    *   **    ****  *   **** * * * * ****          *  *
```

```
h                GGTGCCTCTCCTCTTTCCCTTCATCTTCATCATTTTGACAAAT--TACTCA---------
c                GGTGCCTCTCCTCTTTCCCTTCATCTTCATCATTTTGACAAAT--TACTCA---------
m                GGTACCTTACCTCTTTCCCTTTGTTCCCATCATTCAGAGCAGTGGTCCTCA-TCCTGTGG
r                GGTACTTTACCTCTTTCCCTTTTCTCCCATCATTCAGAGAAGTGGTTCTCAACCCCATGG
                 *** * *  ************      *******  **  * *  * ****

                                 0.386                   MER5B →
h                --------------AAGATGGAGGG----------------TGCAG---TTGAAAGCCAG
c                --------------AAGATGGAGGT----------------TGCAG---TTGAAAGCCAG
m                G---------CTCTGGGGTAGGGGGGGTTGGATGAGTCTTTTGCAGGAGTGGCATATCAG
r                GTCATGACTGCTTTGGGAGGAAGGGGATTGAATGAGTCTTTCACAGGGGTGGCATATCAG
                                *     **                   ***   * * *   ***

h                CCAT---CATT----TTT----------TAAAACTTC--TCAGGACATTGTAACATG---
c                GCAT---CATT----TTT----------CAAAACTTC--TCAGGACATTGCAACATG---
m                ATATTTACATTATGATTCACAACAGTAGCAAAACTACAGTTAGGAAGTAGCAACAAAATC-
r                ATATTTACATCCCAACTTGTAACAGTAGTAAAGATACAGATATAAAGTAGCAACAAG-TA
                   **   ***      *            ***  * *    *  *  * * ****

h                -CACTTGGGTTGAGA---ACTGCTACTCGA---GCT-TCTCCAGGAG-----GCCCTTGG
c                -CATCGGGGTTGAGA---ACTGCTACTCGA---GCT-TCTCCAGGAG-----GCCCTTGG
m                ATTTTATAGTTGGGAGTCACCATGGCCTGAGGAGCTGTATGAAAGGGC---AGCCTTTGG
r                ATATTATAGCTGGGAGTCACCGTAGCATGA--AGCTATATTAAAGGGCTGCAGCATTTGG
                         * ** **   **     *  **   *** * *  * * *     **  ****

h                AGG--------------------------TAGGAAC----------CAG-----------
c                AGG--------------------------TAAGAAC----------CAG-----------
m                AAGGTTGA-GGCCGCTGATTATGAGGCGCTAAGAAAGTGGAGCATACAGATGAAAATAAA
r                AAGGTTAATGATTGCAGGTTAAGAGGAGCTAAGAAGGTGGTGGGTGCAGATGAAAATAAA
                 * *                          ** ***           ***

h                -----------------CATTTGGGCTACTTTAGCATCTTAGTAGAAACTGAAACCAA-A
c                -----------------CATTTGGGCTACTTTAGCATCTTAGTAGAAACTGAAACCAA-A
m                GTA-CCGGTTTGAAAACCTCTCAGGATACTTTAATGTCTAGCCAGAAGACAGAGCTATCA
r                GTATCCTTTTCAAAAACCTCTCAGGATATTTTAATGTCAAGCCAGAAGACAGAACTATCA
                                  *  *  ** ** ****   **     ****     * * *  *
```

TCF4 exon 1

Aligned regions

h   chr18:51449467-51449706
c   chr18:52025588-52025827
rh   chr18:48911757-48911996

note: internal exon 1 is located in SINE (AluSx) and LINE repeats containing region; this exon is used only together with 5' exon 1b.

```
h                CATTTCTTCCCCAGACTACCAGTACCATCTTCTAACTTGTCTACCTGCCTTCATTCTAAC
c                CATTTCTTCCCCAGACTACCAGTACCATCTTCTAACTTGTCTACCTGCCTTCATTCTAAC
rh               CATTTCTTCCCCAGACTACTAGTACCATCTTCTAACTTGTCTACCTGCCTTCATTCTAAC
                 ******************* ****************************************

h                ATGTCCACCATCTTTTCTCCACCCTGCAGCAAATGTTTTGTAAACACCAATCTAAGAACA
c                ATGTCCACCATCTTTTCTCCACCCTGCAGCAAATGTTTTGTAAACACCAATCTAAGAACG
rh               AAGTCCACCATCTTTTCTCCACGCTGCAGCGAATGTTTTCTAAACACCAATCTAAGAACA
                 * ******************** ******* ******** ******************* 

                 → Line2B  AluSx →
h                TTATCTCTTGGACGGGCATGGTGGCTCACACCTGTAATCCCAGCACTTTGGGAGGCCAAG
c                TTATCTCTTGGACGGGCATGGTGGCTCACACCTGTAATCCCAGCACTTTGGGAGGCCAAG
rh               TTATCTCTTGGACGGGCATGGTGGCTCACGCCCGCAATTCCAGCACTTTGGGAGGCCAAG
                 ***************************** ** * *** *********************
h                GTAGGTGGATCACCTGAGGTCAGGTGTTCAAGACAGCCTGGCCAACATGATGAAATCCTG
c                GTAGGTGGATCACCTGAGGTCAGGTGTTCAAGACAGCCTGGCCAACATGATGAAATCCTG
rh               GTAGGTGGATCACCTGAAGTCAGGTGTTCAAGACAGCCTGGCAAACATGATGAAATCCTG
                 ***************** ************************ *****************
```

TCF4 exon 2

Aligned regions

```
h        chr18:53263228-53263515
c        chr18:51988836-51989124
rh       chr18:48870958-48871256
note: internal exon 2 is located in SINE (AluSz) repeat containing region.
this exon is used only together with 5’ exon 1b.
```

```
                                                              AluSz ←
h               CATAGCTTTAAGTGACAGCCAATGCAATGAAGATTTCCATGTGCTGATTTTTTTCC----
c               CATAGCTTTAAGTGACAGCCAATGCAGTGAAGATTTCCATGTGCTGATTTTTTTCC----
rh              CATAGCTTTATGTGACAGCCAATGCAGAGAAGATTTGCATGTGCTGATTTTTTTTTTTTT
                ********** ***************  ******** *****************

                        0.537
h               -------AGATGGAGTCTCATTCTGTCATCCAGACCGGAGTGCAGTGGCACCATCTCAGC
c               -------AGATGGAGTCTCATTCTGTCATCCAGACCGGAGTGCAGTGGCACCATCTCAGC
rh              TTTTTTGAGATGGAGTCTTGTTCTGTCATCCAGACTGGAGTGCAGTGGCACCATCTCGCC
                       ***********  *************** *********************  *

h               TCACTGCAACCTCCACCTCCCAGGTTCAAGCTATCCTCCTGCCTCAGCCTCCTGAGTACC
c               TCACTGCAACCTCCACCTCCCGGGTTCAAGCTATCCTCCCGCCTCAGCCTCCTGAGTACC
rh              TCACTGCAACCTCCACCTGCAGGGTTCAAGCTATTCTCCCGTCTCAGCCTCCTGAGTACC
                ****************** *  ************ **** * ******************

h               CGGGATAGGCGTGAGTCATCACTCGAGGCTAATTTTTGTATTTTTAGTAGAGACGGGG-T
c               CGGGATAGGCGTGTGTCATCACTCGAGGCTAATTTTTGTATTTTTAGTAGAGACGGGGGT
rh              TGGGATCGGCATGTGCCATCACTCCAAGCTAATTTTCATATTTTTAGTAGACACGGGG-T
                 ***** *** ** * ******** * *********  ************* ****** *

h               TTCACCACGTTGGCCAGGCTGGTCACGAACTCCTGACCTCAAGTGATCCACCCACCTCGG
c               TTCACCACGTTGGCCAGTCTGGTCACGAACTCCTGACCTCAAGTGATCCACCCACCTCGG
rh              TTCACCATGTTGGCCAGGCTGGTCACAAACTCCTGACCTCAAGTGATCCACCCACCTCGG
                ******* ********* ******** *********************************
```

TCF4 exon 3a

Aligned regions

```
h        chr18:51409263-51409608
c        chr18:51983884-51984229
m        chr18:69502792-69503123
r        chr18:66016648-66016980
exon 3a sequence identity between h and m: 70%
note: a SINE (MIR3) element is located upstream of this exon
```

```
h               CTAGAGGTAGAGCCAGTGGAAT---TTCTCCTAACCCAACGTTTGTGATCTTTTCATTAC
c               CTAGAGGTAGAGCCAGTGGAAT---TTCTCCTAACCCAACGTTTGTGATCTTTTCATTAC
m               TTGGAGGTAGAGCCAATGGAATATTTTTTCCTACGCCAATGCTTGTGATCTTTTCATTAC
r               TTGGAGGTAGAGCCGATGGAATATTTTTTTCCAAGCCAATGTTTTTGATCTTTTCATTAC
                 * ***********  ******   ** * * *  **** * ** ***************

                     ← MIR3                                         1
h               A--------TCATCTAAGCAGAACCTGT-AGGGGTTAACTCTGAGAATTGGGGATGATGC
c               A--------TCATCTAAGCAGAACCTGT-AGGGGTTAACTCTGAGAATTGGGGATGATGC
m               A-GTGATTTTTTTCCCCCCAGAGCCTGTCGAGGGCTTACTCTGAGAACCAGAGATAATGT
r               ATGATTTTTTTTTTTTCCCAGAGCATGTCAAGGGCTTACTCTGAGAACCAGAGATAATGT
                *        *  *     **** * ***   *** * **********   * *** ***

h               TTGTGATAATGCAGCAAAAAGTAATGTCTAAATGTAAGATATTATTTTGAGATGAGAACA
c               TTGTGATAATGCAGCAAAAAGTAATGTCTAAATGTAAGATATTATTTTGAGATGAGAACA
m               CTGTGATAATGCACTATAAAGAGGTGGCTAAATGTAAAATATTATTTTAGGATGAAAATG
r               CTGCGGTAATGCAGCATAAAGAGGTGGCTAAATGTAAAATATTATTTTAGCATGCAAATG
                 ** * *******  * ****   ** ********** **********   ***  **

h               CCGAATGGGG-ATGGAAGACAGGAATCTTCACCATGTGTGAATGTACAGGAAACAACCAA
c               CCGAATGGGG-ATGGAAGACAGGAATCTTCACCATGTGTGAATGTACAGGAAACAACCAA
m               TGGAATGGGGGATAAAACACTGGAGTCCTCATGATGTGTGAA----------ATAGCCCA
r               TGGAATGGGGGATAAAACACTGGAATCTTCCTGATGTGTGAA----------ATAGCCCA
                  ******** **  ** ** *** ** **   *********          * * ** *
```

```
h               TAAATGCCTTAATTCTTGTTCTGTAAAAACTTACATGTAAGTAAAAGATCGCCGAAATTG
c               TAAATGCCTTAATTCTTGTTCTGTAAAAACTTACATGTAAGTAAAAGATCGCCGAAATTG
m               TAAATGTCCCAATTCTTGTCCTGTAAAA----ACTCTCAAGTAAAACATCTC--------
r               TAAATGTCCCAATTCTTGTCCTGTAAAAG---ACTCTCAAGTAAAACATCTC--------
                ****** *  ********* ********    **    ******** *** *

h               ATCTGAACTCTGTTACTG-CCACAAACTTTTATCAGCAAATGGAGATTAGGATGTTCCCA
c               ATCTGAACTCTGTTACTG-CCACAAACTTTTATCAGCAAATGGAGATTAGGATGTTTCCA
m               -----AATTCTGTCATGGGCCACGAGTTAAAATCAGAAGATAGAGATGAGGACGTTCTCA
r               -----AATTCTGTCACTG-CAGTGAATTAAAATCAGAAAATAGAGATGAGTACGTTCTCA
                     ** ***** *  * *    *  *   ***** * ** ***** ** * ***  **
```

TCF4 exon 3b

```
        Aligned regions
h       chr18:51407362-51408184
c       chr18:51981981-51982803
m       chr18:69504022-69504842
r       chr18:66017864-66018680
exon 3b sequence identity between h and m: 87%
```

```
h               CCGC------TCCCGGAGGAGGCGTGGTCGACCAGCACCGCCATCTTGGCCGCCTCTCTA
c               CCGC------TCCCGGAGGAGGCGTGGTCGACCAGCACCGCCATCTTGGCCGCCTCTCTA
m               CCGCCCTGGTTCCCGGAGGAGGCGTGGCCTAGA-GCGCCGCCATCTTGGCGGCCTCTCTA
r               CCGCCCCGGTTCCCGGAGGAGGCGTGGCTTAGC-GCGCCGCCATCTTGGCGGCCTCTCTA
                ****      *****************   *   ** ************* *********
                                                                       11
h               GGCGCTCTGTTTACCACTCTATGGTCGCCTGCTCCCGCCCCGCCCCTTCCCAGTCATTGT
c               GGCGCTCTGTTTACCACTCTATGGTCGCCCGCTCCTGCCCCGCCCCTTCCCAGTCATTGT
m               GGCGCTCTGTTTACCACTCTATGGTCGCGTGCTCCCGCCCCGCCCCTCCCCAGTCATTGT
r               GGCGCCGTGTTTACCACTCTATGGTCGCGCGCTCCCGCCCCGCCCCTCCCCAGTCATTGT
                *****  ********************* ***** *********** ************
                                         1 2  11   2614
h               CTGGAGGAGCAGCCGCGGCCGCAGCTCCTTCTCTTTATAAGCCCGCAGTTCCCGGATGTG
c               CTGGAGGAGCAGCCGCGGCCGCAGCTCCTTCTCTTTATAAGCCCGCAGTTCCCGGATGTG
m               CTGGTGGAGCAGCCGCGGCCGCAGCGCCTTCTCTTTATAAGCC-GCAGTGCCCGGATGTG
r               CTGGTGGAGCAGCCGCGGCCGCAGCGCCTTCTCTTTATAAGCC-GCAGTGCCCGGATGTG
                **** ******************** ***************** ***** **********
                   4211                            3 1   1
h               AATGGATTACAATGTATCTTTCAGGGAAACCTATTATTATCAATGTGACTCCACGGGGGA
c               AATGGATTACAATGTATCTTTCAGGGAAACCTATTATTATCAATGTGACTCCACGGGGGA
m               AATGGATTACAATGTATCTTTCAGGGAAACCTATTATTATCAATGTGACTCCTCGGGGGA
r               AATGGATTACAATGTATCTTTCAGGGAAACCTATTATTATCAATGTGACTCCTCGGGGGA
                **************************************************** *******
                            1
h               GTCCATGGTGATGATGATGAGGAGGAGGATGATGATGATGAGACACCTCTAAACTTGGAA
c               GTCCATGGTGATGATGATGAGGAGGAGGATGATGATGATGAGACACCTCTAAACTTGGAA
m               GTCAATGATGGTGTTGG------GGAGGAGGATGATGATGAGACGCCTCTAAACTTGGAA
r               GTCAATGATGGTGTTGA------GGATGATGATGATGATGAGACGCCTCTAAACTTGGAA
                *** *** ** ** **       *** ** ************** ***************

h               CAAGTTTAAGACTTTATGAGAGAAGA-----AAAAAAATCACCAACAAGAATTGTTTGAG
c               CAAGTTTAAGACTTTATGAGAGAAGA-----AAAAAAATCACCAACAAGAATTGTTTGAG
m               CAAGTTTAGGACTTTGAAAGAGAAGAGAAAAAAAAATACAACCAACAAGA-----CCGAA
r               CAAGTTTAGGACTTTGAAAGAGAAGA----AAAAAATACAACCAACAAGA-----CCGAA
                ******** ******   ********     ***** *  **********       **

h               GAAAAATTATAACTATCCTGTGTTCATTTTTTTTTTTATAAACAATAAGAAAAAGTTGTT
c               GAAAAATTATAACTATCCTGTGTTGATTTTTTTT--TATAAACAATAAGAAAAAGTTGTT
m               GAACAATTATAACTATCCAGTGTTGATTATTTTT---ATAAACAATACGAAAAAGTTGTC
r               GAACAATTATAACTATCCTGTGTTGATTATTTTTT--ATAAACAATAAGAAAAAGTTGTC
                *** ************** ***** *** *****   ********** ***********
                                           1
h               GGATTTTTTTTT--AATGATTTCTTTTTTGGGGGAGGGAATTTTGTTGCAGTTTTATGGT
c               GGATTTTTTTTTTTAATGATTTCTTTTTTGGGGGAGGGAATTTTGTTGCAGTTTTATGGT
m               GGATTTTTTTTTTTAATGATTACTTTTTGGGGGGAGGGAATTTTGTTACAGTTTGATGAT
r               GGATTTTTTTTT---ATGATTACTTTTTTGGGGGAGGGGATTTTGTTTCAGTTTTATGAT
                ************   ****** ****** ********* ******** ****** *** *
```

```
                                                                
h   GGAAAATGCAAAAACCAGAGCCAGGTGCATAATCTTGTAATCTGTGGATATCCCTGGAGC
c   GGAAAATGCAAAAACCAGAGCCAGGTGCATAATCTTGTAATCTGTGGATATCCCTGGAGC
m   GGAAAATGCAAAAACC-GAGCCAGGTGCATAATCTTGTAATCTGTGGCTAACCCTGGAAC
r   GGAAAATGCAAAAACCAGAGCCAAGTGCATAATCTTGTAATCTGTGGCTAACCCTGGAAC
    **************** ****** *********************** ** ******* *
                                                               1
h   AGGACTGAGTACCAGTTAAAATACTTTTT---GGGGATACACATGTGAGATACTAAGTAC
c   AGGACTGAGTACCAGTTAAAATACTTTTT---GGGGATACACATGTGAGATACTAAGTAC
m   AGGACTGACTTCTATTTAAAATACTCTTTTGGGGGAACACTCATGTGAGACACTAAGTTC
r   AGGACTGACTACTATTTAAAATACTCTTTTGGGGGAATACACATGTGAGATACTAAGTAC
    ******** * * * ********** ***   *** * ** ********* ******* *
              1
h   TTGCAGAAGATTTTTGTCTCTCTTTTTAAAGTCTCTTTCCTTGGAATATTGTGAGAATAT
c   TTGCAGAAGATTTTTGTCTCTCTTTTTAAAGTCTCTTTCCTTGGAATATTGTGAGAATAT
m   TTGCAGAAGATTTTTGTCTCTCTTTTTAAAGTCTCTTTCCTTGGAATATTGTGAGCATAT
r   TTGCAGAAGATTTTTGTCTCTCTTTTTAAAGTCTCTTTCCTTGGAATATTGTGAGCATAT
    ******************************************************* ****

h   TTGTGGCCATTTAAGGTAACGTTTCAATTTGCCGTCAGAGTAAACTGTTTGTAATTGAAT
c   TTGTGGCCATTTAAGGTAACGTTTCAATTTGCCGTCAGAGTAAACTGTTTGTAATTGAAT
m   TTGTGGCCATTGAAGGTAACGTTACAATTTGCTGTCAGAGTAAACTGTTTGTAATTGAAC
r   TTGTGGCCATTGAAGGTAACGTTACAATTTGCTGTCAGAGTAAACTGTTTGTAATTGAAC
    *********** *********** ******** ************************** 

h   TTAATTTTTAAAATGTCGAT-CCCGATGTTTTATTAAAACAAAAGGGACAACCTATTAAA
c   TTAATTTTTAAAATGTCGAT-CCCGATGTTTTATTAAAACAAAAGGGACAACCTATTAAA
m   TGAATTCTTAGAATGTTGAG-CCCAATGTTTTATTCAAACAAAAGGAACAACCTATTAAA
r   TGAATTTTAAGAATGTTGATTCCCAATGTTTTATTCAAACAAAAGGAACAACCGATTAAA
    * **** * * ***** **  *** ********** ********** ****** ******

h   TAATTGCCAATGGGAAAAAGTGTGTGCATGCATTTTGTGTATGTACTCGCACATCTAGCA
c   TAATTGCCAATGAGAAAAAGTGTGTGCATGCATTTTGTGTATGTACTCGCACATCTAGCA
m   TAATTGCCAGTGGGAAAACGTGTGTGCATGCATTTCC-GTACGTACTCGTACATTTAGCA
r   TAATTGCCAGTGGGCAAACGTGTGTGCATGCATTTCG-GTGCGTACTCGCACATTTAGCA
    ********* ** * *** ****************   **  ******* **** *****
```

TCF4 exons 3c, 3d

```
        Aligned regions
h       chr18:51406110-51407003
c       chr18:51980740-51981619
m       chr18:69505196-69506161
r       chr18:66019053-66020010
exon 3c sequence identity between h and m: 63%
exon 3d sequence identity between h and m: 80%
```

```
h   TCG-CCCCCAACGCAAAACTACCGAAACGAAA-ATCTCCC-CAGGAAAA--TGTGGCTTG
c   TCG-CCCCCAACGCAAAACTACCGAAACGAAA-ATCTCCC-CAGGAAAA--TGTGGCTTG
m   TCGGCCCCAAACGCAAAACTATCCAAACAAAATATCCCCC-TAGTAAAAA-TGTGGATGG
r   TCGGCCCCAAACGCAAAACTATCCAAACAAAATATCCCCCCTAGTAAAAAATGTGGATGG
    *** **** ************ * **** *** *** ***  ** ****  ***** * *

h   ATTTATAGACGAAAAAGAAAAATATTGTTCCTCTTGGCAAAGTCTTTTTGCATCACGTGT
c   ATTTATAGACGAAAAAGAAAAATATTGTTCCTTTTGGCAAAGTCTTTTTGCATCACGTGT
m   GTTTCTGTATGGAAAAGAAAAATATTGTTCGTCTTGGCAAAGTCTTTATGCATCACGTGT
r   CTTTCTGTATGGAAAAGAAAAATATTATTCGTCTTGGCAAAGTCTTTATGCATCACGTGT
     *** *  * * ************** *** * ************** ************
             1                    2           1
h   ATTTGCAGCCTAGTATAAACTTGCTTTGCCGTGTGTGGATGTGTG---------------
c   ATTTGCAGCCTAGTATAAACTTGCTTTGCCGTGTGTGGATGTGTG---------------
m   ATTTGCAGCAAAGCA-AAACTTGCTTTGCAGTGTGTGTGTGTGTGTGTGTGTGTGTGTGT
r   ATTTGCAGCAAAGCA-AAACTTGCTTTGCAGTGTGTGTGTGTGTGTGTGTGTGTGTGTGT
    *********  ** * ************* *******  ******

h   ---------------------------AGTGAGAGGGAACGAGAGTAAGAGAAAGAAA--
c   ---------------------------AGTGAGAGGGAACGAGAGTAAGAGAAAGA----
m   GTGTGTGTGTGTGTGA--GAGAGAGAGAGAGAGAGAGAGAGAGAGAGAGAGAGAGAGACA
r   GTGTGTGTGTGTGTGTGTGTGAGAGAGAGAGAGAGAGAGAGAGAGAGAGAGAGAGAGAGT
                               ** ***** **  *****  ***** ***
```

```
                                               2
h   --------------------------GAAGTGAG--GGGATGTAAACTCGAATAAATTTC
c   ----------------------------AGTGAG--GGGATGTAAACTCGAATAAATTTC
m   TACAGAGAC--ACAGAGAGACAGACAGAAGTGAGAAGGGATGTAAACTCGAATGAATTTC
r   TACAGAGACAGACAGAGAGACAGACAGAAGTGAGAGGGGATGTAAACTCGAATCAATTTC
                                ******  ***************** ******
    1
h   AAAGTGCCTCCGAGGGATGCAACGGGCAAAAACTGAACTGTTCAGGCTTCAGATTGTAAC
c   AAAGTGCCTCCGAGGGATGCAACGGGCAAAAACTGAACTGTTCAGGCTTCAGATTGTAAC
m   AAAGTGCCTCCGATGGATGAACCCGGCAAACCCTGAACTGTTCAGGCTTCAGATTGTAAC
r   AAAGTGCCTCCGGTGGATGAACCCGGCAAATCCTCAACTGTTCAGGCTTCAGATTGTAAC
    ************  ***** * * ******  ** *************************

h   TGACGATCTGAGG--AAAAATGAGGTGCTCGATGAATTTTCGTTTGTA------------
c   TGACGATCTGAGG--AAAAATGAGGTGCTCGATGAATTTTCGTTTGTA------------
m   TGGCGATCTGAGGGGGAAAATGAGGTGCTCGGTGAATTTTCCTTGGTGGGTTTTTTTTTT
r   TGGCGATCTGAGGGGGAAAATGAGGTGCTGGGTGAATTTTCCTTGGTGGGTTTTGTTTTT
    ** **********   ************* * ********* ** **

h   -------TTTTTTGGCGAGGCGGGGGAGGTGTTGAGATTTTTTTTTTTTCCCCTCGGGGT
c   -------TTTTTTGGCGAGGCGGGGGAGGTGTTGAGATTTTTTTTTTT-CCCCTCGGGGT
m   CCCCTCTCTCTTTCTCAAGACGGGGGAGGTGTTGGGATTTTT----TTTCT----GGGGT
r   T---TTTTTCTTCCTCGAGACGGGGGAGGTGTTGGGATTTTTTTTTTTTCT----GGGGT
            * **   * ** ************** *******    ** *     *****

h   GGGTGCGAGGGGGATGCATCCTAGCCTGCCCGACCCGGAGCAAGTCGCGTCTCCCCGCCG
c   GGGTGCGAGGGGGATGCATCCTAGCCTGCCCGACCCGGAGCAAGTCGCGTCTCCCCGCCG
m   GGG---------GGTGTTTCCCAGGCAGCCCGGGCCGGAGC--GCCGCGTCTCCCCGCCG
r   GGG---------GGTGTTTCCTAGGCAGCCCGACCCGGAGC--GCCGCGTCTCCCCGCCG
    ***         * **  *** ** * *****  ******* * ***************
h   GAGCCCCCCCACCCATTTCTTTGCTGAACTTGCAATTCCGTGCGCCTCGGCGTGTTTCCC
c   GAGCCCCCCCACCCATTTCTTTGCTGAACTTGCAATTCCGTGCGCCTCGGCGTGTTTCCC
m   GAGCCCCCCCACCCATTTCTTTGCTGAACTTGCAATTCCGTGCGCCTCGCCGTGTT-CCC
r   GAGCCCCCCCACCCATTTCTTTGCTGAACTTGCAATTCCGTGCGCCTCGCCGTGTT-CCC
    ************************************************* ****** ***
                                            1      1 1
h   CCTCCCCCCTTCCCTCCGTCCCCTCCCCTCCCCGGAGAAGAGAGTTGGTGTTAAGAGTCA
c   CCTCCCCCCTTCCCTCCGTCCCCTCCCCTCCCCGGAGAAGAGAGTTGGTGTTAAGAGTCA
m   CCTCCCCCCTTCCCTC-GTCCCCTCCCCTCCCGGGAGAAGAGAGTTGGTGTTAAGAGTCA
r   CCTCCCCCCTTCCCTC-GTCCCCTCCCCTCCCGGGAGAAGAGAGTTGGTGTTAAGAGTCA
    **************** *************** ***************************
                2       2          1
h   GGGATCTTGGCTGTGTGTCTGCGGATCTGTAGTGGCGGCGGCGGCGGCGGCGGCGG----
c   GGGATCTTGGCTGTGTGTCTGCGGATCTGTAGTGGCGGCG---------GCGGCGG----
m   GGGATCTTGGCTGTGTGTCTGCGGATCGGCGGCGGCGGCGGCGGCG---GCGGCGGCGGC
r   GGGATCTTGGCTGTGTGTCTGCGGATCGGCGGCGGCGGCGGCGGCG---GCGGCGG----
    *************************** *  * *******         *******
                        3
h   -----------GGAGGC---AGCAGGCGCGGGAGCGGGC-GCAGGAGCAGGCGGCGGCGG
c   -----------GGAGGC---AGCAGGCGCGGGAGCGGGC-GCAGGAGCAGGCGGCGGCGG
m   AGCGGCGGTGGGGAAGCCGAAGCGGGCGCGGGCGCCGGCCGTTGGAGGAGGTGGTGGTGG
r   -----------GGAAGCCGGAGCGGGCGCGGGCGCCGGCCGTTGGAGGAGGTGGTGGTGG
               *** **   *** ******** ** *** *  **** *** ** ** **

h   TGGCGGC-GGCGGTTAGACATGAACGCCGCCTCGGCGCCGGCGGTGCACGGAGAGCCCCT
c   TGGCGGC-GGCGGTTAGACATGAACGCCGCCTCGGCGCCGGCGGTGCACGGAGAGCCCCT
m   CGGAGGCAGGGGGATAGACATGACCGCCGCCTGGGCGCCGGCGCTGCACCGGGAGCCCCT
r   CGGAGGCAGGGGGATAGACATGACCGCCGCCTGGGCGCCGGCGCTGCACCGGGAGCCCCT
     ** *** ** ** ********* ******** ********** ***** * ********

h   TCTCGCGCGCGGGCGGTAGGTACCGGCGCCTGCGGGGCTCGGCGGGGCGGAGGCGCCCGG
c   TCTCGCGCGCGGGCGGTAGGTACCGGCGCCGGCGGGGCTCGGCGGGGCGGAGGCGCCCGG
m   CGTCGCGCGCGGGCGGTAGGTAGCGGCGC-GGCGGGGC-CGGCTGGGCGGATGCGC--GG
r   CGTCGCGCGCGGGCGGTAGGTAGCGGCGC-GGCGGGGC-CGGCTGGGCGGATGCGC--GG
      ******************** ******  ******* **** ******* ****  **
```

```
h               CGGCGCGGGGTT----CGGGCTCGGCGGCCCCGCACGCGGCTCCGCGCCTCCCG--CGCC
c               CGGCGCGGGGCT----CGGGCTCGGCGGCCCCGCACGCGGCTCCGCGCCTCCCG--CGCC
m               CGGGGCCCGGCCGGGCCGGGCTGGGCGATCGCGCTCG-GGCTCGGGGCCCCGTGGTCCCC
r               CGGGGCCGGGCCGGGCCGGGCTGGGCGGCCGCACACG-GGCTCGGGGCCCCGTGGTCCCG
                *** **  **      ****** ****  * * * ** ***** * *** *  *  * *

h               GCGGGCTCC--CGGCGCCCGGCGCTCCCAGAAGAGACACCCCTTCCCCTCCCGCCGCTTC
c               GCGGGCTCC--CGGCGCCCGGCGCTCCCAGAAGAGACACCCCTTCCCCTCCCGCCGCTTC
m               GCTGTCCCCGCCGAGGTTCGG-GAGCTCGGGAGCGACG-----------------GCGTC
r               GCTGTCCCCGCCGGGGTTCGG-GAGCTGGGGAGCGGCG-----------------GCGTC
                ** * * **  **  *  *** *  *   * ** * *                  ** **
```

TCF4 exons 4a (I,II,III), 4b, 4c, 4

```
        Aligned regions
h       chr18:51403400-51404497
c       chr18:51978044-51979134
m       chr18:69507820-69508908
r       chr18:66021490-66022567
exon 4a sequence identity between h and m: 67%
exon 4b sequence identity between h and m: 73%
exon 4c sequence identity between h and m: 77%
```

```
h               AAATAAAATCCAAACCGCCTTCCAAGTGGGGCTCTTTCATGCTGCTGCTGCTGCTGCTGC
c               AAATAAAATCCAAACCGCCTTCCAAGTGGGGCTCTTTCATGCTGCTGCTGCTGCTGCTGC
m               AAATAAAATCCAAACCACCTTCGAAGTGGGGCTCCCCCACGCTGCTCCTGCTCC-ACTGC
r               AAATAAAATCCACACCACCTTCTAAGTGGGGCTCCCCCACTCTGCTCCCGCTCCTGCTGC
                ************ *** ***** ***********   **  ***** * *** *  ****

h               TGCTGCTGCTGCTGCTGCTGCTGCTGCTGCTGCTGCTGCTGCTGCTGCTGCTGCTCCTCC
c               TGCTGCTGCTGCTGCTGCTGCTGCTGCTGCTGCTGCTGCTGCTGCTCCTG------CTCC
m               --CTGCCGCTGCTG--GCTGCCACCACTGCTGCT--------------------------
r               --CTGCCGAAGCTG--GCTGCCACCTCTGCTGCT--------------------------
                  **** *  ****  *****  *  ********
                                             1          3  21
h               TCCTCCTCCTCCTTCTCCTCCTCCTCCTCCTCTTCTAGACCTTCTTTTGGAGAAATGGCT
c               TCCTCCTCCTCCTTCTCCTCCTCCTCCTCCTCTTCTAGACCTTCTTTTGGAGAAATGGCT
m               ----------------------------CCTCTGGCAGACCCTGT---GGAGAAGTGGTT
r               ----------------------------CCTCCCGCAGACCCCGT---GGAGAAGTGGTT
                                            ****    *****   *   ****** *** *
                    1    12
h               TTCGG--AAGTTTTGCCAGGAAAC-GTAGCCCTAGGCAGGCAGCTTTGCAGCCCCCTTTC
c               TTCGG--AAGTTTTGCCAGGAAAC-GTAGCCCTAGGCAGGCAGCTTTGCAGCCCCCTTTC
m               CTCCGGAAAGTTTTGGCAGGAAGCAGTAGCCC---ATAGGCGGCTTTGCAGCCCCCTTTC
r               CTCTG-AAAGTTTTGGCAGGAAGCAGTGGCCC---ATAGGCGGCTTTGCAGCCCCCTTTC
                 ** *  ******** ****** * ** ****     **** ******************
                          1
h               TGCTTGTTGCACTTTCTCCATTCGTTCCTTTGCTTTTT-GCAGGCTCTGACTCAGGGAAG
c               TGCTTGTTGCACTTTCTCCCTTCGTTCCTTTGCTTTTT-GCAGGCTCTGACTCAGGGAAG
m               TGCTTGTTGCACTTTCTCCATTCGCTCTTTTGCTTTTTTGCAGGCTCTGACTCAGGGAAG
r               TGCTTGTTGCACTTTCTCCATTCGCTCCTTTGCTTTTTTGCAGGCTCTGACTCGGGGAAG
                ******************* **** ** ********** ************** ******
                I   1                1
h               GTGTGCATTATC-CACTAGATACGTCGAAGAAGAGGGAAACCAATTAGGGTCGAAATAAA
c               GTGTGCATTATC-CACTAGATACGTCGAAGAAGAGGGAAACCAATTAGGGTCGAAATAAA
m               GTGTGCGAAAACGCTTTAGAC-TGGAGGAGAAGAGGGAAATCAGTTAGGGCGGAAATAGA
r               GTGTGTGAAA-CGCATTAGAC-TGGAGGAGAAGAGGGAAACCAGTTAGGGCGGAAATAGA
                *****    * * *  ****   *  * ************ ** ******  ****** *
                0.223
h               TGCTGG------AGAGAGAGGGAGTGAAAGA-----------------------------
c               TGCTGG------AGAGAGAGGGAGTGAAAGA-----------------------------
m               TGCTGGAGAGTGAGAGAGAGGGAGTGAAAGATTGAGAGAGAGAGAGAGAGGGTGAGAGAG
r               TGCTGGAGAGAGAGAGAGAGGGAGTGAAAGATTGAGAGAGAGTGAGAGAG----------
                ******      *******************
```

```
                                     II
h       ------------GAGAGTGAGAGTGAGAGAGAGAGAGAGT--------------------
c       ------------GAGAGTGAGAGTGAGAGAGAGAGAGAGT--------------------
m       ACAGGAGAGAGAGAGAGAGAGAGAGAGAGAGAGAGAGAGAGAGAGAGAGAGAGAGGCTGAGAGA
r       -----AGAGAGAGAGAGAGAGAGAGAGAGAGAGAGAGAGA--------------------
                    ***** ***** ***************

           1    1                                          III
h       CTTGCTTCAAATTGCTCTCCTGTTAGAGACGAAATGAGAATTT---AGTGCAGGTGGCAC
c       CTTGCTTCAAATTGCTCTCCTGTTAGAGACGAAATGAGAATTT---AGTGCAGGTGGCAC
m       CTTGCCT-ACGTTGCTCTCATGTTTGAGCTGAAGTGAGAAATTGTTACTGCAGGTGGCAT
r       CTTGCCT-ACGTTGCTCTCATGTTTGAGCTGAAGTGAGAAATT---ACTGCAGGTGGCAT
        ***** * *  ******** **** ***  *** ****** **    * ***********

                                          1           0.275 0.484
h       TTTTATTTTTATTTGGGTTCACATATGACAGGCAAATCCTATACGAGATGGAAATGGACA
c       TTTTATTTT-ATTTGGGTTCACATATGACAGGCAAATCCTATACGAGATGGAAATGGACA
m       TTTGATTTTGATTTGAGTTCATGTATGGCAGACAAGTCCTGTAACTGATGGAAGGGGACA
r       TTTGATTTTGATTTGGGTTCATGTGTGGCAGACAAGTCCTGTAACGGATGGGAAGGGACA
        *** ***** ***** *****  * ** *** *** **** **    ***** *  *****

                  0.333
h       TTGCCACGTTTATG-----GCCAAGGTTTTCAATATAAAACAAAACAACTTTTTTCTTCT
c       TTGCCACGTTTATG-----GCCAAGGTTTTCAATATAAAACAAAACAACTTTTTTCTTCT
m       TTGCCACATTTATGTTATGTCCAAGGGTTTCAATATAAAACAAA-CAACTTTTTTCTTCT
r       TTGCCACATTTATGTTATGCCCAAGGTTTTCAATATAAAACAAA-CAACTTTTT-CTTCT
        ******* ******      ****** **************** ********* *****

h       CCTTGGTGAAACTAGTGTTTTTCTAGAGAGGCTGCTGG------------CCTCCAACCT
c       CCTTGGTGAAACTAGTGTTTTTCTAGAGAGGCTGCTGG------------CCTCCAACCT
m       CCTTTGTCAAACTAGTGTTTT-CTAGAAAGGCTGCTGT------------CCCCCTATGT
r       CCTTTGTCAACGTGGTGTTTT-CTAGAAAGACTGCTGTGTCCCCCCCCCCCCCCCAATCT
        **** ** **  * ******* ***** ** ******             ** ** *  *

h       GAATCTTGATAACATTATGGGGACTGTGTTTGTTCCAAATGTAGCAGTAGTACTGCTTGG
c       GAATCTTGATAACATTATGGGGACTGTGTTTGTTCCAAATGTAGCAGTAGTACTGCTTGG
m       ATGCCTTACTAATGCCATGGGGACTGTGTTCA----GAATGTGG---TAGGACTGTTTA-
r       ATGTCTTGATAATGCCTTGGGGACTGTGTTCA----GAATGTAG---TAGGACTGTCTAG
            ***  ***     *************       ***** *    *** ****   *

h       CCATCTAATGAACCTGAGGAAAAAGAAAGAACAGAGTGATAATGGGGGCTGGGGTGGGAT
c       CCATCTAATGAACCTGAGGAAAAAGAAAGAACAGAGTGATAATGGGGGCTGGGGTGGGAT
m       -------AGTCACCCGAGGA----GAAAGCTAACAGTGGTAATGGAGG--GAGGTGGAGG
r       CCATCTAAGTCACCCGAGGAGGA-GAAAGCTA-CAGTGGTAAAGGAGG--GAGGAGGAGG
               *    *** *****     *****      **** *** ** **   * ** **

h       CTGTAATGTTGTT-TCTCTTTTAGTTTTAAGTTGG------ATGGTGATGTATTTTACTA
c       CTGTAATGTTGTT-TCTCTTTTAGTTTTAAGTTGG------ATGGTGATGTATTTTACTA
m       TTGGAACAG-GCTGCCTGGTTTAGTTTTAGGTTGG------ATGGCTATATGTTTTCCTA
r       TTGGAACCCCGGTGCCTGGTTTAGTTTTAAGTTGGTTGGTGACGGCTCTATGTTTTCCTA
         ** **     * *  **  ********** *****       * **    * * **** ***

                                      1
h       AATAAACCCTTAGCATAAACTCTAAGCTGTTTGGTAACAGTATGAAAGATCTTTGAGGAG
c       AATAAACCCTTAGCATAAACTCTAAGCTGTTTGGTAACAGTATGAAAGATCTTTGAGGAA
m       AATAAAGCCTTAGCATTAACTCCAAGTTATTCATCAAGAGTGTGAGAGATCTTTGGTGAA
r       AATAATGCCTTAGCATTAACTCCAAGTTATTTATCAAGAGTGTGAGAGATCTTTGGTGAA
        *****  ********* ***** *** * **     ** *** *** *********  **

                                                         0.223-0.477
h       CTCTGAAGGCACAAGTGTCTTCTTTTCAACTGTAATATTTCTTTGTTT-CTTTTAGATGT
c       CTCTGAAGGCACAAGTGTCTTCTTTTCAACTGTAATATTTCTTTGTTT-CTTTTAGATGT
m       CTCTGAAGCCACGAATGTCTTCCTTTCAACTCTGCTGTCTCTTTGTTT-CCTTTAGATGT
r       CTCTGAAGCCACGAATGTCTTCCTTTCAACCATGCCATCTCTTTGTTTTCCTTTAGATGT
        ******** *** * ******* *******  *     * ********* * *********

h       TTTCACCTCCTGTGAGCAGTGGGAAAAATGGACCAACTTCTTTGGCAAGTGGACATTTTA
c       TTTCACCTCCTGTGAGCAGTGGGAAAAATGGACCAACTTCTTTGGCAAGTGGACATTTTA
m       TTTCGCCTCCTGTAAGCAGTGGGAAAAATGGACCAACTTCTTTGGCGAGTGGACATTTCA
r       TTTCGCCTCCTGTAAGCAGTGGGAAAAATGGACCAACTTCTTTGGCGAGTGGACATTTTA
        **** ******** ******************************** *********** *
```

```
h               CTGGCTCAAGTATGTAAATTCTTTTCTTAGTGT-TAAGTTAAATGTTCTGGGCTGTTTAT
c               CTGGCTCAAGTATGTAAATTCTTTTCTTAGTGT-TAAGTTAAATGTTCTGGGCTGTTTAT
m               CTGGCTCAAGTACGTGAATTCTTTTCTTGGTGTTGAAGTTCAATGGTCTCAGTTGTTTAT
r               CGGGCTCAAGTACGTGAATTCTTTTCTTGGTGT-GAAGTTCAATGTTCTGGGTTGTTTAT
                * ********** ** ************ ****  ***** **** ***  * *******

h               TTACTCAATTGTATGTTTTTGGCTCTGTTGAAGAGTTCTGAAA-AAGTCATTGAATTTTA
c               TTACTCAATTGTATGTTTTTGGCTCTGTTGAAGAGTTCTGAAA-AAGTCATTGAATTTTA
m               TTGCTCT-TTGTGTATTTTTGGCTCTGTTGGAGTGTTCTGAAAGAAGTCATTGAGTTTTA
r               TTGCTCT-TTGTGTATTTTTGGCTCTGTTGGAGTGTTCTGAAAGAAGTCATTGAGTTTTA
                ** ***  **** * *************** ** ********* ********** *****
```

TCF4 exon 5a (I,II)

Aligned regions

```
h       chr18:51364943-51365292
c       chr18:51938781-51939130
m       chr18:69544313-69544647
r       chr18:66056297-66056645
```

exon 5a sequence identity between h and m: 79%

```
h               GAATCTGTCTGGAATCGGCGCGGAACTTTGGAGAATGTTTTCTCCTCAGGAGCTGGAACT
c               GAATCTGTCTGGAATCGGCGCGGAACTTTGGAGAATGTTTTCTCCTCAGGAGCTGGAACT
m               GAATCTGTCTGCAACCAGCAGGGTGCACTGGGTG-CG-TTGGTCCTCAGGAGCTGGGTCT
r               GAATCTGTCTGGAACCAGCGGGGCGCATTGGGTACCAATTGCTCCTCAGGAGCTGGGCCT
                *********** ** * **  **  *  ***       **  **************  **

h               CAACTCTTCCTCA-GCCCACA-CAATGCAGGATCTGTGGTTTTCTGTCCCTGTTTCTTTT
c               CAACTCTTCCTCA-GCCCACA-CAATGCAGGATCTGTGGTTTTCTGTCCCTGTTTCTTTT
m               CCACACACCC----ACCCTCC---GCGCAGGATCCCAGAT---------CTCCTTTCTCT
r               CCACCCCTCCCGACACCCACCTCACTGCTGGATCTGAGAT---------CTAGTTTCTTT
                * ** *  **     *** *      ** *****   * *         **  **  * *
                           1
h               TCTCCATTTTTCTCCCCTTCACTTACTCTGATTGATGGAAACACGTGCT--GGGAGCTTT
c               TCTCCATTTTTCTCCCCTTCACTTACTCTGATTGATGGAAACACGTGCT--GGGAGCTTT
m               TCTC--TTTCCCCTCCCTTCCCTGGCTCTGATTGATGGAGACACGTGCT-GGAGAGCTCT
r               TCTCTCTTTCCCCTCCCTTCACTTGCTCTGATTGATGGAAACACGTGCCCGGGGAGCTTT
                ****  ***  *  ****** **  ************** ********   * ***** *
                                           I                           0.260
h               CCCTGACATTCTTTCCAAAGACTGAAAGTAAGAAATGTTATGGTGGTG------CTATGT
c               CCCTGACATTCTTTCCAAAGACTGAAAGTAAGAAATGTTATGGTGGTG------CTATGT
m               TCCTGACAATCTTCCCAAACACGGAGAGAGGGAGATGCTATGGTGGTGGTG---CTGGCT
r               CCCTGACAATCTTCCCAAACGCAGAAAGAAGGAGATGCTATGGTGGTGGTGGTGCTGGGT
                 ******* **** *****  * ** **   ** *** **********      **   *
                            II
h               CTATTTTTGAAAGTAAGTCTGTTAACGTAAAGCCTGTGTTGATATATTGGGATGAATTAT
c               CTATTTTTGAAAGTAAGTCTGTTAACGTAAAGCCTGTGTTGATATATTGGGATGAATTAT
m               CTATTTTTGAAAAGAAGACTGTTAATGTAAAGCCGGTGTTGATATATTCGGGTGAATTAT
r               CTATTTTTGAAAAGAAGGCTGTTAATGTAAAGCCGGTGTTGATATATTCGG-TGAATTAT
                ************  *** ******* ******** ************* ** ********

h               AATGGTGATATTATTACAGCCTTCAAAGCTGAAAATTTATTATTTTTCTCACCTACTTTT
c               AATGGTGATATTATTACAGCCTTCAAAGCTGAAAATTTATTATTTTTCTCACCTACTTTT
m               AATGGGGATATTATTACAGCCTTCCAAGCTGAAAATGTATTATTTTTCTCACCTACTTT-
r               AATGGGGTTATTATTACAGCCTTTCAAGCTGAAAATGTATTATTTTTCTCACCTACTTT-
                ***** * ***************  *********** ********************** 
```

TCF4 exon 5b

```
        Aligned regions
h       chr18:51328689-51329127
c       chr18:51902034-51902472
m       chr18:69575418-69575840
r       chr18:66088428-66088869
exon 5b sequence identity between h and m: 53%

h               TATTTTATTTTTGTTTCAGAAAAGGTCTGCTATTGTTCAAATTTCTAAT--GTAGCTCAT
c               TATTTTATTTTTGTTTCAGAAAAGGTCTGCTATTGTTCAAATTTCTAAT--GTAGCTCAT
m               TAGTTTATTTTTGTTGCGGTAAAGGTCTTCTATTGTTCTGATTTCTAACTCGCGGCTCAC
r               TATTTTATTTTTGTTTCGGTAAAGGTCTGCTATTGTTCAGATTTCTAAC--GCGGCTCAT
                ** ************ * * ******** *********  ********   *  *****

h               AAACACACAGCCAGTATCATTTCCCCTTCTACTGACTGATATATACAAATAAATTAAAAT
c               AAACACACAGCCAGTATCATTTCCCCTTCTACTGACTGATATATACAAATAAATTAAAAT
m               AAACACACAGTCCATATCCTCTCCCCTTCTCCTGACTGATGTGTAC-AATAAATGAAAAC
r               AAACACACAGCCAATATCCTCTCCCCTTCTCGTGACTGATGTATACCAACAAATGAAAAT
                ********** *  **** * *********  ******** * *** ** **** ****

                                    1  2  3    1
h               CTACGG------------TCATCATTAACTCATGTTCTGTTTCCATGGAGCACAGGAGAG
c               CTACGG------------TCATCATTAGCTCATGTTCTGTTTCCATGGAGCACAGGAGAG
m               CTCCAG---------TCATCGTCATTAGTCAGAGTTCTGTCTCTGTGAAGGGTAGAAAAG
r               CTCCCGCCGCCGCCGTCATCATCTTTAGTCAGAGATCGGTGTCTATGGACGGTAGGAAAA
                ** * *            ** ** ***      * ** ** **  ** *    ** * *
                                                                 1 1
h               TAACAAGGAAGGCCCCT-TAAAAGAAAGGCCTTTTAA-AGTGGAGGT-CTCAGTTCACAA
c               TAACAAGGAAGGCCCCT-TAAAAGAAAGGCCTTTTAA-AGTGGAGGT-CTCAGTTCACAA
m               GAACAAGGGAGACTTCTTCAGAAGAAAGTCCCTTTGAGAGCAGTGGTTCTCAGCCTGTAG
r               CAACAAGGGAGAGTCCT-CAGAAGAAAGTCCCTCTGAGAGCAGTGATTCTCAACCTGTGG
                 ******* **    **  * ******* ** * * * **  * * * ****

h               ACTTTAAAAGTTTACGAAGTAGTATTTTAGA-ATT--------------TTAA--TCACC
c               ACTTTAAAAGTTTACGAAGTAGTATTTTAGA-ATT--------------TTAA--TCACC
m               GTCGAGACCCCTTTGGTGGCTGAATATCAGATA----------------TTAGTGTTACA
r               GTCGTGACCCCTCCGGTGGCTGCGTGTCAGATAGCCAGCTTATCAGAAATTAGTGT-GCC
                      *    *   *  *  *  * * *** *                ***   *  *
                                                  0.379
h               AGATATATTGGGGGAATTTATTACTTCCTATTCATATGGAAGAGGACAGCAGAGGTAAAT
c               AGATATATTGGGGGAATTTATTACTTCCTATTCATATGGAAGAGGACAGCAGAGGTAAAT
m               ACACATGGCAGTAGCAAG-ATTGCC----ATT---ATGAAGTAG--CAACACAAATAATT
r               ATACAT--CGGTAGCAAG-ATCACA----GTT---ATGAAGCAG--CAGTAAAAATAATT
                * * **    *  * *   **  *      **   *** *  **  **  * *  *** *

h               ATTTATGTAACTTGTATTTTAAAAGCTTACTCTTGATTAAAATGTTTGATCTATCGACTT
c               ATTTATGTAACTTGTATTTTAAAAGCTTAGTCTTGATTAAAATGTTTGATCTATCGACTT
m               --TTATGA----------CTGGGGGGTCA-CCACAACACAGGGGACTGTTTTAAAGGGA-
r               --TTATGA----------TGGGGGGTTCA-CCACAACCCAGGGAACTGTTATAA-GGGT-
                  *****                 * * *  *   *   *      ** * **  *

h               CTGTATCCTTAGAAGTTTGA-----ATTGCTGGACATATTTGACTGGGGTTTTTA--CAA
c               CTGAATCCTTAGAAGTTTGA-----ATTGCTGGACATATTTGACTGGGGTTTTTA--CAA
m               CATAGCATTTGGAAGGTTGAGAGACACTGCTG-------TGGAGGGAGATTCTTATCCGT
r               CAGAGCATTTGGAAGGTTGAGAGCCACTGCTA-------TAGAAGGAGATTCTAATCCAC
                *       ** **** ****     * ****        * **  * * ** * *  *
```

TCF4 exon 5c

Aligned regions

```
h        chr18:51298148-51298550
c        chr18:51871746-51872156
m        chr18:69605796-69606177
r        chr18:66119076-66119463
```

exon 5c sequence identity between h and m: 57%

note: exon 5c is located in SINE (MIRb) element containing region.

```
                                                 MIRb ←
h               TAATATCCTCCCCCCGCCCCCCCGCTTTTTTTTTTTTTTTTTTTTTGGAGTTCCTACTGTG
c               TAGTATCCTCCCCCCGCCCCCCTGCTTTTTTTTTTTTTT-------GAGTTCCTACTGTG
m               TAATAAAATTG----GGAGTTATGTCTATACAGTTAT----------AA-TCCTACTATG
r               TAATAAAATTG----GGAGTTATACCTATAGATTTTT----------AGCTCCTACTACG
                ** **   *      *          * *    ** *          *  *******  *

                                            2   1
h               TTCCAGGTTCCATGGTAACATTTTCCTTGTTATCTGTAATCATCACAACAGTACTGGAGG
c               TTCCAGGTTCCATGGTAACATTTTCCTTGTTATCTGTAATCATCACAACAGTACTGGAGG
m               TTCCAGGTTTTATGGCAACACTTTCC-------CTATACCTTTCATGACAAT-CCAGAGA
r               TTCAAGGTTTCATGGTAACACTTTCC-------CTATACTTTTCACAACAGT-CCAGAGA
                *** *****  **** **** *****       ** **    ***  *** * *  ***

                                                                  0.367
h               GTAAATACGGTCATTCCCATTTTAC-AGATGAGGATACATTCAATGT-GAGATGTTAAAG
c               GTAAATACGGTCATTCCCATTTTAC-AGATGAGGATACATTCAATGT-GAGATGTTAAAG
m               GCAGAAGCAGTGACTCCCATTTTGTTGGATGAGAATCCATACAGTAT-GAGATATCAAAA
r               GGACAAGCAGTCCTTACCATTTTGTTGGATGAGACGCCATACAGTACAGAGATATCAAAA
                * * *  * **   * *******    ******    *** ** *   ***** * ***

                0.389
h               ATGTTCTTCAGAATTTCCCAGGTAGCAGGCAGCTCA---------------TGGTATTCA
c               ACGTTCTTCAGAATTGCCCAGGTAGCAGGCAGCTCACCACACCACCATGGGTGGTATTCA
m               ATGTCCCCCAG--TTGTCCGCAAAGCAGGCAGCTCCGCACACTCTCGTGGGTGGTGCTCG
r               AGGTCCTCAAA--TTGTCCGCAAAGCAGGCAGCTGAGTACACTCTCGTGGGTGGTGTGGG
                * ** *   *   **  **    ***********                 ****

h               AAAGGTAATGGTTAAGAGAAAGAATGGATAATGAATATCTTGTGTCTATTTTCTTGAGAC
c               AAAGGTAATGGTTAAGAGAAAGAATGGATAATGAATATCTTGTGTCTATTTTCTTGAGAC
m               GAAGGTAATGGT--GGAAAGGCGACA----CTAAATGCATTCTGTTTGTTTTCTTGAGAC
r               AAAGGTAATGGT--GGAACGGCGATAGATCCTAAATGCATTCTGTTTATTTTCTTGAGAC
                 ***********   **      *       * ***   ** *** * ************

h               AGACGAGACTGAGCAGCCTGAGAACATTTGGTGCTCAGAGGGAGGAAATGCTAAGAGGGG
c               AGACGAGACTGAGCAGCCTGAGAACATTTGGTGCTCAGAGGGAGGAAATGCTAAGAGGGG
m               AGCC-----TGAGCAGCCTGAGAACATTTGGTGCTCAGAGGGAGGAAGCGCTGAGAGGGG
r               AGAC-----TGAGCAGCCTGAGAACATTTGGTGCTCAGAGGGAGGAAGTGCTGAGAGGAG
                ** *     **************************************  *** ***** *

h               AAAACAAGTCTGAAAGGGAAAGAAGGATAGTTCAAAGACCAGTTTCCCCTGCTTCAGAAT
c               AAAACAAGTCTGAAAGGGAAAGAAGGATAGTTCAAAGACCAGTTTCCCCTGCTTCACAAT
m               AAAACAAGTCTGAGTGG-AAAGGAGGGTAGTTCAAAGACCAGTTTCCCCTGCTTCACCGT
r               AAAACAAGTCTGAATGG-GAAGGAGGAGAGTTCAAAGACCAGTTTCCCCTGCGTCGTCGT
                *************  **  *** ***  ************************ **    *
```

TCF4 exons 7a (I, II, III)

Aligned regions

```
h        chr18:51240265-51240881
c        chr18:51813659-51814272
m        chr18:69660167-69660803
r        chr18:66174462-66175075
```

exon 7a sequence identity between h and m: 76%

```
h               AACTTTTCAGGGAAGAAAAAAA--CACACACACACACTAAAAGCACCAGATGCAAATTAA
c               AACTTTTCAGGGAAGAAAAAAA--CACACACACACACTAAAAGCACCAGATGCAAATTAA
m               AACTTTTCAGGGAAGAAAAAAAAACACACACACACACTAAAAGCACCAGATGCAAATTAA
r               AACTTTTCAGGGAAGAAAAAAA--CACACACACACACTAAAAGCACCAGATGCAAATTAA
                **********************  ************************************
```

```
h    TGGTAGGGAGTCTT--TAACTCTTTAAACCACCCCCCTAAATTTCCATCATAGGAAACAT
c    TGGTAGGGAGTCTT--TAACTCTTTAAACCACCCCCCTAAATTTCCATCACAGGAAACAT
m    TGGTAGGGAGTCTTTTTAACTCTTTAAACAACCCCCCTAAATTTCCATCCGAGGAAACAC
r    TGGTAGGGAGTCTTTTTAACTCTTTAAACAACCCCCCTAAATTTCCATCGGAGGAAACAC
     **************  ************* *******************  ********
                                1                              2
h    GT-GAT--GAGTGTTAGCCTAAGAACTAGATTCTC-----------TTTTTTTTAATAAT
c    GT-GAT--GAGTGTTAGCCTAAGAACTAGATTCTC-----------TTTTTTTTAATAAT
m    GTCGGTTGGAGTGTTAGCCTAAGAGCAAGTTCTTTATGATTATTATTTTTTTTTAATGAC
r    GTCGGTCGGAGTGTTAGCCTAAGAGCTAGTTTTTTATAATTACTATTTTTTTTTAATGAC
     ** * *  **************** * ** *  *            *********** *

h    AG----GAAAGTACTTTTATTTGTGACCCAAGTATGAGAATTTCTGGCAAACTTTTTTTT
c    AG----GAAAGTACTTTTATTTGTGACCCAAGTATGAGAATTTCTGGCAAACTTTTTTTT
m    AGCAACGAGAGTGCTTTTATTTGTGGCCCAAGTATGAGATATTCTAATAAACTTTTTTT-
r    AGCAATGAAAGTGCTTTTATTTGTGGCCCAAGTATGAGAAATTCTAACAAACTTTTTT--
     **    ** *** ************ *************  ****   **********

h    TAATATTAAAAAAAAC----CCCCC---AAACCCTCCACCTTTTCCCTTCTTTCCTTTTC
c    -AATATTAAAAAAAAC----CCCCA---AAACCCTCCACCTTTTCCCTTCTTTCCTTTTC
m    -AACATTAAAAAAAATAAAGCCCCATATGACCCCTCCACCTTTTTGTTTCTTTCCTTTTC
r    -AATATTAAAAAAAAAAAAGCCCCATGTGACCCCTCCACCTTTTTGTTTCTTTCCTTTTC
      ** ***********     ****     * *************   *************
                               1                 1
h    CCTTTCAAGACTATTTAAATGTACATAGTAAAACATGTTAGGACAGCCAAGCCCCAGCAT
c    CCTTTCAAGACTATTTAAATGTACATAGTAAAACATGTTAGGACAGCCAAGCCCCAGCAT
m    CCTTTTAAGACTATTTAAATTTACATAGTAAAACACGTCAGGACAGCCAAGCCG--GCGT
r    CCTTTCAAGACTATTTAAATTTACATAGTAAAACATGTCAGGACAGCCAAGCCC--GCGT
     ***** ************** ************** ** **************   ** *
                             2                            3     1
h    GTTGTGTTCTGTCCTCTGGCTGGCAGATTCACGCCACAACAGTTTATTCATCCACATGCT
c    GTTGTGTTCTGTCCTCTGGCTGGCAGATTCACGCCACAACAGTTTATTCATCCACATGCT
m    GTTCTGTTCTGTCCTCGG-CTGGCACAGTGACACCACAACACTTTATTCCTCCACATGAT
r    GTTCTGTTCTGTCCTGGATCTGGCTCACTGACACCACAACACTTTATTCCTCCACATGAT
     *** ***********    *****  * * ** ******** ******* ******** *
                                   1 1
h    TTTTGGCGACTCTTCATCACGTATTTGGGCTTAGATCTTCTGGTGCCTCCCTCGGCGTGC
c    TTTTGGCGACTCTTCATCACGTATTTGGGCTTAGATCTTCTGGTGCCTCCCTCGGCGTGC
m    TTT-GGCGACTCTGCCTCATGTATTAGTGCT----------GGTGCCTCCCTCTGCCTGC
r    TTT-GGCGACTCTGCCTCACGAATTAGAGCT----------GGTGCCTCCCTCTGCCTGC
     *** ********* * *** * *** * ***          ************ ** ***
               I           II                        III
h    TACCCATAAGGTCGAAAGAAAAGCAAGTATTCG-ATTTCTCTATTCAGGGTGGGTTTTTT
c    TACCCATAAGGTCGAAAGAAAAGCAAGTATTCG-ATTTCTCTATTCAGGGTGGGTTTTTT
m    CGCCCAGAAGCCCAGAAGAAAAGCAAGTACCCC-GTTTCTCT--TTAGGGTGGGCTTTTG
r    CGCCCAGAAGCCCCGAAGAAAAGCAAGTACCCCCGTTTCTCC--TCAGGGTGGGCTTT-G
       **** ***  *  **************  *   ******   * ******** ***

h    TTTTTTTTTTTTTTTTTGGAT---GATAGTGGTTTTTTTTTTT------TT--GTGCTTT
c    TTTTTTTTTTTT----TGGAT---GATAGTGGTTTTTTTTTTT------TTTTGCGCTTT
m    TTTGGTCTTGTTG---TGCGTTTAGATACTTGTTTGCCCACCCACCCACCCCCATGCTCT
r    TTCGGTTTTGCTG---TGCGTTTAGATATTTGCTTTTCC----------CTGAATGCTTC
     **   * **  *    **  *   **** * * **                    ***

h    TT-CTTTTTAATCTATTCCCTTTGTGGTTTAG-TGTAAGAGTTTGAGAATGTGCCCATAA
c    TT-CTTTTTAATCTATTCCCTTTGTGGTTTAG-TGTAAGAGTTTGAGAATGTGCCCATAA
m    TTACTTTCTAACCTGTTCCCTTTGCGGTTTAGCTA-GAGGTTTGGAGAACCCGCCCTTCA
r    TTACTTTCTAACCTGCTCCCTTTGCGGTTTAGCTGCGAGGTTTGGAGACCCTGCCCTGCA
     ** **** *** **  ******** ******* *    **  ** ****    ****   *
```

TCF4 exons 7b (I,II), 7

```
        Aligned regions
h       chr18:51221530-51222336
c       chr18:51794937-51795741
m       chr18:69679014-69679822
r       chr18:66192823-66193608
exon 7b sequence identity between h and m: 86%

h               GTAGGCAATTTTTCAAAAGCCCTGTTTCCCAAATGTAAATTCCATCATCA-TCTTGATCA
c               GTAGGCAATTTTTCAAAAGCCCTGTTTCCCAAATGTAAATTCCATCATCA-TCTTGATCA
m               ATAGGTAATTTCCCCAA-ACCCCCTTTCCCAAGGGGG----GCCCCATCACTGCTGCTGG
r               CTAGGTGACTTCTCCAA-GCCCCCTTTCCCAAGTGTG----CCCT---------------
                 ****  * **  * **  ***  ********  *       *

h               TGATGGGGTTCACTCCTGGG-AAAGGGAGTTTTTAAAAAAGAG-AGAGAGAG--------
c               TGATGGGGTTCACTCCTGGG-AAAGGGAGTTTTTAAAAAAGAG-AGAGAGAG--------
m               TAATGGGGATCACTCCTGGGGAAAGGGAGTTTTTGAAAGGGAGGAGAGAGCAGTGGGGGG
r               ------GGATCAGTCCTGGGGAAAGGGAGTTTTTAAAAGGGAGGAGAGAGAGGTGGGGGG
                      ** *** ******* ************* ***  *** ******
                    2
h               -AGAGACTCAGAATTTTGAATAGTTTCTTTGCTAAATTCACCATCTGGAGAGCCTGTGAT
c               -AG--ACTCAGAATTTTGAATAGTTTCTTTGCTAAATTCACCATCTGGAGAGCCTGTGAT
m               AAGGGTCTTAGAATTTTGAACAGTTTCTTTGCGAGATTCACCATCTGGAGAGCCCATGAT
r               AGGGGGCTTAGAATTTTGAACAGTTTCTTTGCTAAATTCACCATCTGGAGAACCCATGAT
                  *   ** *********** *********** * **************** **  ****

h               TGATTAGTTTTGGCCTCACTATTAAGTGTCAATATAATCAATGGGAATTACTAGGCTAGG
c               TGATTAGTTTTGGCCTCACTATTAAGTGTCAATATAATCAATGGGAATTGCTAGGCTAGG
m               TGATTAGCTTTGGCCTCCCTGTTAAGTGTCAATATAATCAATGGGAAGTACCAGGCTAGG
r               TGATTAGCCTTGGCCTCCCTGTTAAGTGTCAATATAATCAATGGGAAGTACCAGGCTAGG
                *******  ******** ** ************************** * * ********

h               GTCATTACATAGATAATTTGCCTGCTGTGTAAATAAAAATGAAATTGTTTAAATTTTGTG
c               GTCATTACATAGATAATTTGCCTGCTGTGTAAATAAAAATGAAATTGTTTAAATTTTGTG
m               GTCATTACATAGATCATTTGCCTGCTGTGTAAATAAAGATGAAATGGTCCAAATTTTGCG
r               GTCATTACATAGATCATTTGCCTGCTGTGTAAATAAAAGTGAAATGGTTCAAATTTGGTG
                ************** **********************  ****** **  ****** * *

h               CAAGAGAAAG----TGAAAATAA--GGAGTTACGATTTGTTTGTTA-GAGAAAGTGGAGG
c               CAAGAGAAAG----TGAAAATAA--GGAGTTACGATTTGTTTGTTA-GAGAAAGTGGAGG
m               AGAGAGAGAGAACGTGAAAAACAA-GGAGTGACAATTTGTTTGTTAAGAGAAAGTGGAGG
r               AGAGAGGAAGAACGTGAAAAAAAAAGGAATGACAATTTGTTTGTTA-GAGAAAGTGGAGG
                  ****  **    ******  *  *** * ** ************ *************
                  1                   11      2  1                 0.514
h               CCATTGGAATGACAGTTTTTGGAAGTGTGGAGCAGTTTGGCTAAGAATAGGAATGAAGGA
c               CCATTGGAATGACAGTTTTTGGAAGTGTGGAGCAGTTTGGCTAAGAATAGGAATGAAGGA
m               CCTCTGGAATGACAGTTTTTGGAAGTGTGGAGCAGTTTGGCTAAGAATAGGAATGAAGGA
r               CCACTGGAATGACAGTTTTTGGAAGTGTGGAGCAGTTTGGCTAAGAATAGGAATGAAAGA
                **  ***************************************************** **
                1 11  1    (11)  1                 1       1 2 3
h               TATTTTTTT-CCAGTTTATCATAGCCAGAGTGAGGAAGTGTTATTCCCTCTCTTGCTTGC
c               TATTTTTTT-CCAGTTTATCATAGCCAGAGTGAGGAAGTGTTATTCCCTCTCTTGCTTGC
m               TATTTTTTTTCCAGTTTCTTGTCGCCAGAGTGAGGAAGCGCCATTCCCTGTCTTGCTTGC
r               TATTTTTTT-CCAGTTCCTTGTCGCCAGAGTGAGGAAGCGCCATTCCCTGTCTTGCTTGC
                ********* ******  *  * *************** *  ******* **********
                 11       2                I 1
h               ATACATTGCCAGTAGTGCCTACTTTACGTATGTAAACATCGGGGAAGAGCAGTAGATGTC
c               ATACATTGCCAGTAGTGCCTACTTTACGTATGTAAACATCGGGGAAGAGCAGTAGATGTC
m               ATACATTGCCAGTTGTGGCTCCCCTACGTATGTAAACATCGGAGCTGGGGAGTGGATCTC
r               ATACATTGCCAGTTGTGTCTCCTCTACGTACGTAAACATCCGAGATGGGGAGTGGATCTC
                ************* *** ** *  ****** ********* * *  * * *** *** **

h               TGTTACCTG--GAGGAGATTTTTTTTTAAGGAAAAACACAATTTACACTTTTTTGTCGTT
c               TGTTACCTG--GAGGAGATTTTTTTTTAAGGAAAAACACAATTTACACTTTTTTGTCGTT
m               TGTTACCTGCAGGGGAAGTTTGCTTTAGAGGAAAAGCACGATTCATCCTGTTCTGTTGTT
r               AGTTACCTGCAGGGGAGGTTTGCTTTAAAGGAAAAGCACGATTTATCCTGTTTTATTGTT
                 ********  * ***  ***  ***  ******* *** *** *  ** ** * * ***
```

```
                           II
h               TCTCTTGGCAGGTAAAACAGAAAGGGGCTCATACTCATCTTATGGGAGAGAATCAAACTT
c               TCTCTTGGCAGGTAAAACAGAAAGGGGCTCATACTCATCTTATGGGAGAGAATCAAACTT
m               TCTCTTGGCAGGTAAAACAGAAAGGGGCTCATACTCATCTTATGGGAGAGAA---AACGT
r               TCTCTCGGCAGGTAAAACAGAAAGGGGTTCATACTCATCTTATGGGAGAGAA---AACGT
                ***** ********************* ************************   *** *

h               ACAGGGTTGCCACCAGGTAAGTGAGAATCTCTGT-TGCGGAAGGGTTGAGAGGATTAACC
c               ACAGGGTTGCCACCAGGTAAGTGAGAATCTCTGT-TGCGGAAGGGTTGAGAGGATTAACC
m               TCAGGGTTGCCACCAGGTAAGTCCCACATT-GGGCTGGGGACGAGGTGAGAGGGCTAACC
r               ACAGGGTTGCCACCAGGTAAGTGCCACTCTCGGTCTGGGGAAGAGGTGCCAGAACTAAGG
                 *********************   *   *  *  ** *** * * **  **   ***

h               TGGTAAATCA--GTTTGTTTTCTAAATTAGATGATGCATTTTAAACTGTGACACCAAAGA
c               TGGTAAATCA--GTTTGTTTTCTAAATTAGATGATGCATTTTAAACTGTGACACCAAAGA
m               ATGGAAATGATTGCCTGCCTT----------------GCTTTCAGTGGTGACACTGAAGG
r               AGGGAAATGGT-GCCTGTCTT----------------GCTCTCAGTTGTGATACTAAAGG
                  * ****    *  **  **                  * * *   **** **  ***

h               TGCACCGGCAAG--TCAGA------TGATGGCTCTCTCTTTAAGTAATGAAATAACACAG
c               TGCACCGGCAAG--TCAGA------TGATGGCTCTCTCTTTAAGTAATGAAATAACACAG
m               ---CCAGGGAGGGTTGAGAGTCTCCTGATGGCTGTCT--TTAAGGAATGAGGCAAACCGG
r               ---ACAGGGAGGGCTGAGAGCCTC-TGACGGCTGTCT--TTAAGGAACGAAGCAAATCGG
                    * ** * *  * ***      *** **** ***  ***** ** **   **  * *
```

TCF4 exons 8a, 8b (I,II) and 8c (I,II)

```
        Aligned regions
h       chr18:51219075-51220606
c       chr18:51792487-51794015
m       chr18:69680798-69682354
r       chr18:66194620-66196287
exon 8a sequence identity between h and m: 83%
exon 8b sequence identity between h and m: 79%
exon 8c sequence identity between h and m: 69%
```

```
h               AA--ATGTAAGCAAAGTTTTGTTTTTGTAATTAAATCGAGCCTATGAGGAAAGTTTCCTT
c               AA--ATGTAAGCAAAGTTTTGTTTTTGTAATTAAATCGAGCCTATGAGGAAAGTTTCCTT
m               ACCCATGTAAGCCCACATGTGCTTCAGTAA-----CTGGGCAC-TGAGGGAAGTCTCTGT
r               AAC-ACGTAAGCCCACATGTGCCTCAGTAATGTAACTGGGCAC-TGAAGGAAGTTTTCCT
                *   * ******  *  * **  *  ****       * **   *** * **** *   *

h               TAC-GTACCAGACAT-----AGGAAGGTACGACTTCTGGTGGGTTGGT-------GTGTT
c               TAC-GTACCAGACAT-----AGGAAGGTACGACTTCTGGTGGGTTGGT-------GTGTT
m               ACCAGGAGCGCTCATTTCTCATGAAGGTA-GAATTCTGGAGGATTGGTTCTGGACGGGTT
r               TCCAGGAGCACCCGTTGATCATGAAGGTA-GAATTCTGGAGGATTGGTTCTGGAAGGGTT
                  * * * *   * *     * ******* ** ****** ** *****       * ***
                                                         1
h               TGTTTTT---TCCCTG------TATATATTTGCATTTTTAAAGTTTACTCCCTTGGCCCA
c               TGTTTTT---TCCCTG------TATATATTTGCATTTTTAAAGTTTACTCCCTTGGCCCA
m               TTCTTTCCCCTCCCCGCTGCTGTATATATTTGCATTTTAAAAGTTAACTCCTCTGGCCCA
r               TTCTTTCCCCTCCCCGCCG---TATATATTTGCATTTTAAAAGTTGACTCCTCTGGTCCA
                *  ***    **** *      **************** ****** *****  *** ***
                   1           3         2
h               GCCACCAATTCAGTCATATTC-GGCATCTGAAAGATTTGTTGCATTGAGGTGAACAGTGC
c               GCCACCAATTCAGTCATATTC-GGCATCTGAAAGATTTGTTGCATTGAGGTGAACAGTGC
m               GTTGCCAATTCAGTCATACTCCAGCATCTAAAAGATTTGTTGCGATGAGCAGCGCAGCTC
r               GTTGCCAATTCAGTCATCTCCCAGCATCTAAAAGATTCGTGGCGATGAGCTGCACAGCTC
                *   *************   *  ****** ******* ** **  ****  *  ***  *
                       1
h               TTGGTTAAGAGCTCCTGCCGGAGTG-CAGTTCATGGCAGTTTGTTTGGAAAGAATTCTGC
c               TTGGTTAAGAGCTCCTGCCGGAGTG-CAGTTCATGGCAGTTTGTTTGGAAAGAATTCTGC
m               TGGGTTGAGAACTCTCGCAGGAGGGGCAGCCCATGGCAGTCTGTGTGGAAAGAATTCTGC
r               TTGGTTGAGAAGTCTTCCAGGAGGG-CAGCCCATGGCAGTTTGTGTGGAAAGAATTCTGC
                * **** ***  **   * **** * ***  ********* *** ***************
```

```
                    1
h  TCATGGCAGAGGTCTCTG------TTTTCGCTGGGCCCCTTTTTCTGGTCATTGAGAGAA
```

```
h  AGGAAAGAAATTTACAGCCATTCCAGCGCAACGGAAACATCTTTGGAATGTGAGTGCAAA
c  AGGAAAGAAATTTACAGCCATTCCAGCGCAACGGAAACATCTTTGGAATGTGAGTGCAAA
m  AGGAAAGAAACTTACAGCCATTCCAGTGCAACGGAAACATCTTTGGAATGTGAGTGCACA
r  AGGGAAGAAATTTACAGCCATTCTGGTGCAACGGGAACATCTTTGGAATGTGAGTGCACG
   *** ****** ************  * ******* ***********************

h  TGTTCGGGCTAGATAGCCTGGGCGACAAGCCTCTG-AGTGCTGTGTCTCTGGTGTAAA-G
c  TGTTCGGGCTAGATAGCCTGGGCGACAAGCCTCTG-AGTGCTGTGTCTCTGGTGTAAA-G
m  TGTTCGGGGCAGATAGCCTGGGCAACCAGCC-TGACAGAGCTGGGTCTCGGGTGTAAAAG
r  TGTTCGGGGCAGATAGCCTGGGCAACCAGCCCTTACAGCGCTGGGTCTCTGGTGTATA-G
   ********  ************* ** ****     ** **** ***** ****** * *

h  CTCTCAGTTCCTGCAGAAGACCGTGTTCCTGTGGGTCTGGTTTCTGAGGGTCAAGGCGGA
c  CTCTCAGTTCCTGCAGAAGAGCGTGTTCCTGTGGGTCTGGTTTCTGAGGGTCAAGGCGGA
m  CTCTCGATTCCTGCAGAAAAGCCGGTTCCTGTGGGTCTGGTTTCTGAGGGTCAAGGCAGA
r  CTCTCAGTTCCTGCAGAAAAGCCGGTTCCTGTGGGTCTGGTTTCTGAGGGTCAAGGCAGA
   *****  *********** * *  ********************************* **

h  GGTCATTAGAGTTTTGGGGAGAAGAAAGTTGACAACA---TCCTTCCTTTCTGCAGATGG
c  GGTCATTAGAGTTTTGGGGAGAAGAAAGTTGACAACA---TCCTTCCTTTCTGCAGATGG
m  GGTCGCCAGAGTTTTGGGGAGAAGAAAGTTAACAACAGCATCCTTCCTCTTTGCAGATGG
r  GGTCGCCAGAGTTTTGGGGAGAAGAAAGTTAACAACA---TCCTTCCTCTTTGCAGATGG
   ****   *********************** ******   ******** * *********
                    1
h  TCATGGCAGAGGTCTCTG------TTTTCGCTGGGCCCCTTTTTCTGGTCATTGAGAGAA
c  TCATGGCAGAGGTCTCTG------TTTTCGCTGGGCCCCTTTTTCTGGTCATTGAGAGAA
m  CCGAGGCACAGGTCTCTAGCTTTTTTTCCGCTGGGGCCCTTTTGCTGGTCATTGAGAGAA
r  TCATGGCACAGGTCTCTA-----TTTTTCGCTGGGCCCCTTTTGCTGGTCATCGAGAGAG
    *  **** ********       *** ******* ******* ******** ******
                             1  11 1
h  TAGGCTTCCTCACCCCTGTATCCTTTCTTACTAATAGGAATTGGCGTGATTCCTCACAGA
c  TAGGCTTCCTCGCCCCTGTATCCTTTCTTACTAATAGGAATTGGCGTGATTCCTCACAGA
m  TAGGCTTCCTAGACCCTGTATCCTTTCTTACTAATAGGCATTGGCATGATTCCTCACAGA
r  TAGGCTTCCTAGACCCTGCATCCTTTCTTGCTAACAGAAATTGGCATAATTCCTCACAGA
   **********   ***** ********** **** **  ****** * ************
                                                          1
h  CACAAAGATTTCCTCTGCTGAGTAAGCGTGAGGCCCCTTAACTTGTGAAAGCATCATCCA
c  CACAAAGATTTCCTCTGCTGAGTAAGCGTGAGGCCCCTTAACTTGTGAAAGCATCATCCA
m  CACAGAGATTTCCTCTGCTGAGTAAGCCAGAGGTCCCTGAACTTGGGAAAGCATCATCCA
r  CACAGAGATTTCCTCTGCTGAGTAAGCCTGAGGCCCCTGAACTTGGGAAAGCATCATCCA
   **** **********************  **** **** ****** **************
         I
       1 3     1           1
h  GACCGTGTGAGTCTGTCTGTGTATGTGCAGAACACAGACCCTCCTTTCTCCGT-------
c  GACCGTGTGAGTCTGTCTGTGTATGTGCAGAACACAGACCCTCCTTTCTCCGT-------
m  GACCTTGGGAGTAAATGTGTGTA----CAGAGCGCAGTCCCTTCTCCCTCCGT-------
r  GACCGTGGGAGTCAGTGTGTGTA----CAGAGCGCAGTCCCTTCTCCCTCCGTGTTTTTG
   **** ** ****   * ******    **** * *** **** **  ******

h  --------------------------------------------------TTGT---GGG
c  --------------------------------------------------TTGT---GGG
m  ------TTTTCTTT-------------------------------TTTTCTTTT----GG
r  TTTTTGTTTTTTTTGGGGGGGGGTTGTTTGTTTGTTTGTTTGGGTTTTTTTTTTCCAGGG
                                                     ** *    **

h  GAATACTTCCCTCGGGTGAAACTGAAGTTAATTTTTTTTT----------TTTTCCAAAC
c  GAATACTTCCCTCGGGTGAAACTGAAGTTAATTTTTTTTT----------TTT--CAAAC
m  GAATACTTCCTTCGG-TGAAACTGAA-----CTTTTTTTTGTTTGTTTGTTTT--CAAAC
r  GAATACTTCCTTCGG-TGAAACTGAA-----CTTTTTTTT-----------TT--CAAGC
   ********** **** **********      ********           **  *** *
        II                         1
h  ACAAGGTGAGAAGTGGAAAGGGGCAGAGGGTGGAAGAGAGGTGGATGGATTG--GCATGT
c  ACAAGGTGAGAAGTGGAAAGGGGCAGAGGGTGGAAGAGAGGTGGATGGATTG--GCATGT
m  ACAAGGTGAG-AGTGGAATGGGATGTGGGG-GGATTTGAGGTGG-TTGCCTGGAGTGTGT
r  ACAAGGTGAGGAGTGGAGTCGGACATGGGG-GGAATTGAGGTGG-TCGACTGGAGTGTGT
   ********** ******   **     *** ***   ******* * *  **  *  ***
```

```
                               I
h   GATGGCTGAATTTCTGGGCCTTGGGAGGTTCGTTTTGAGGCATTAATGTCCAGGAAAATT
c   GATGGCTGAATTTCTGGGCCTTGGGAGGTTCGTTTTGAGGCATTAATGTCCAGGAAAATT
m   GAAGGCTAAAATGTCGGG-----AG--CTTGACT---AG---CTGCTATTCAGGAAAACT
r   GAGGGCTGAAAT-TCGGG-----GGGCCTTCACT---AG---TTGCTCTTCAGGAAAATG
    ** **** ** *   ***      *   **   *   **    *  * * ********

h   CTTTTGTATATAGCACTATGACTGCATATGCTTTTCTTAAAAAGCCTTCGTAATGTAGCC
c   ATTTTGTATATAGCACTATGACTGCATATGCTTTTCTTAAAAAGCCTTCGTAATGTAGCC
m   CTTTTTC-TATA---TCAGGGCTGT---TGCTTTTCTTGAGAGGCCGTCAGAATCTTGCT
r   CTTTT-C-TATC---CCAGGGCCGT---TGCTTTTCTTGAGAGATCTTCAGAATCTTGCT
     ****   ***      * * * *    ********** * *   * **  *** * **

h   GTGG-AGTGTGGAGATTCCTTCATTCTTAATTTGTGCTTCATGGTTGTGAGTCTTGCAAG
c   GTGG-AGTGTGGAGATTCCTTCATTCTTAATTTGTGCTTCATGGTTGTGAGTCTTGCAAG
m   GGGGGAGTGTGGCGCAGCCTTCCTTCTTAGTTC-TGCTTCAGGCATGCGAGTCTTTCGAG
r   GGGG-AGTGTGGCGCTGACT-CCTTCTTAGTTC-TGCCTCAGGCTTGCGAGCCTTGCGAG
    * ** ******* *    ** * ****** **  *** *** *  ** *** *** * **

h   AGTCTACTATCTCTGT------GCTTAGCGTGGTCGCAGCAAT-----CTCCATAAACAG
c   AGTCTACTATCTCTGT------GCTTAGCGTGGTCGCAGCAAT-----CTCCATAAACAG
m   CAATGGCTATCTGTGC------GCTCAGCTAGGTCTTAGCTGC-GTCCACCTAGAAACCG
r   AAATGGCTATCTGTGCCTGCGCGCTCAGCTAGGTCTTAGCTGTTGTCCACCCATATACAG
          ****** **       *** ***  ****  ***          * * * ** *

h   TGTGATTACTTAGGGATAAATAAAAGTGAAATTTTGGAAATTTGCTGACAGAGGGAAAAT
c   TGTGATTACTTAGGGATAAATAAAAGTGAAATTTTGGAAATTTGCTGACAGAGGGAAA-T
m   TGTGAT-GCTCAAGGGTATCTCGAGG-GAAATGCTGAGACTTCCCTG-CGTGGGGAAA-C
r   TGCGAT-GCTCAAGGGTGTCCCCTGG-GGAATGTTGAGAATTCCCTG-TGTGGGGAAA-T
    ** ***  ** * ** *        * * ***  **  * **  ***     ******
                                        1
h   AGGGACTGAAGATGAAAA-ATGTACAGAGTATGAATTTCTGCTTTCTGTGAAATTTGAGA
c   AGGGACTGAAGATGAAAA-ATGTACAGAGTATGAATTTCTGCTTTCTGTGAAATTTGAGA
m   AGGGCTTGAGGGTGAGGATGCGTAAGCAGTCTGGATTTCAGCGTTCTGTGGAATTCGAGA
r   AGGGCTTGAAGATAAGAGTGTGTACATAGTCTGGATTTCAGCTTTCTGTGGAATTCGAGC
    ****  *** * * *      ***   *** ** ***** ** ******* **** ***
                                   1
h   ATAGAACAGACCATAGTGTGT-CTTCCAGTGAATCAGTTTCTCTTGTGCCGTATTGTTAT
c   ATAGAACAGACCATAGTGTGT-CTTCCAGTGAATCAGTTTCTCTTGTGCCGTATTGTTAT
m   AAAGAGCAGAGCAGAGTTTGT-CTCTCAGGGAGTCGGTTTCTCTGGAGCTGCCTTGTTCT
r   AAAGAGCAGGGCGAAGTTTGTTCTCTCAGGGAGTCGGTTTCTCTGGCGCTGCATTGTTCT
    * *** ***  *  *** *** **  *** ** ** ******** * ** *  ***** *

h   TTGAATCCCTGTCTACAACATCAGCTATGCAAACCAAAGAGAAAGGCAAAATCTCAGTAT
c   TTGAATCCCTGTCTACAACATCAGCTATGCAAACCAAAGAGAAAGGCAAAATCTCAGTAT
m   TTGAACCCTTGTCTACTACTTGAGCTAAACAAACCCCAAAGAAAGCCAGAATCCCCGCGT
r   TTGAACCCTTGTCTACTACTTGTGCTAAACAAACCCCAAAGAAAGCCAGAATCCCGGTGT
    ***** ** ******* ** *  ****  ******  * ****** ** **** * *  *
                                             1
h   C------TTGGGCTTCAAGTCTAAATAGCAGATCACAGCTGAAATGATTCCCCACTGTGT
c   C------TTGGGCTTCAAGTCTAAATAGCAGATCACAGCTGAAATGATTTCCCACTGTGT
m   C------TTGTGCTCGGAGTCTAAATAGCAGATCACAGCTGAAATGATTCCCCACTGTGG
r   CGGTGTCTTGGGCTTGGAGTCTTAGTAGCGGATCACAGCTGAAATGATTCCCCGCTGTGT
    *      *** ***   ***** * **** ******************* *** *****
          II
h   GACAAGGTGAGT------------GACTCGT-----------------------------
c   GACAAGGTGAGT------------GACTCGT-----------------------------
m   AACAAGGTGAGT------------GACT-GTCT---------------------------
r   AACAAGGTGAGTCTCAGTCTGTCTGTCT-GTCTGTTTCTCTCTCTCTCTCTCTCTCTCTC
     ***********            * ** **

h   -------------------------------------GGAGGGGAGGCTTTTGTTTTCTT
c   -------------------------------------GGAGGGGAGGCTTTTGTTTTCTT
m   -------------------------------CTCTTGGGAAGGGGAGCTTTTGTTTGCTC
r   TCTCTCTCTCTCTCTCTCTCTCTCTCTCTCTCTCTCAGGAAGGGGAGCTTTTGTTTCCTT
                                         *** ***  ********** **
```

```
h               CTGTTTACTACATGATTGAGATACGGAATAGGAAACCTTCAGATGGTGTGCCCTAAGGAG
c               CTGTTTACTACATGATTGAGATACGGAATAGGAAACCTTCAGATGGTGTGCCCTAAGGAG
m               C-GTTTGCCA-ACTAGTGAGATGCTGGACAGAAAACCTTTAGATGGAGTAACCCCAGGAG
r               C-ATTCGCCA-GCTAGTGAGTCCCTGGACAGAAAACCTTTACATGGGGTAGTCCCAGGAG
                *  **  * *    * ****   * * * ** ******* * **** **   *  *****

h               CTCCAAAAGCTGCTTTATCGTATTATAACAGTGTA-TCCTATGGACTTAAAGGGGTGAGC
c               CTCCAAAAGCTGCTTTATCGTATTATAACAGTGTA-TCCTATGGACTTAAAGGGGTGAGC
m               CTGCAAAAGCTGCCTTTTCATATGATAACAATGTA-GCCTGTGAACCTAAAGGGATGAGC
r               CTGCAAA-GCTGCCTTTTCATATGATAACAATGTAAGCCTGTGAACCCAAAGGGATGGGC
                ** **** ***** ** ** *** ****** ****  *** ** **  ****** ** **
```

TCF4 exon 8d

```
         Aligned regions
h        chr18:51169899-51170366
c        chr18:51742009-51742476
m        chr18:69722547-69723022
r        chr18:66238485-66238964
exon 8d sequence identity between h and m: 95%
```

```
h               GGGGCAGAGTCATAATTGGCCTATTTAGAGTATTTTGCATGTGAATAGTGTGTTAGTGAA
c               GGGGCAGAGTCATAATTGGCCTATTTAGAGTATTTTGCATGTGAATAGTGTGTTAGTGAA
m               GGGGCAGAGTCATAATTGGCCTATTTAGAGTATTTTGCATGTGAATAGTGTGTTAGTGAA
r               GGGGCAGAGTCATAATTGGGCTATTTAGAGTATTTTGCATGTGAATAGTGTGTTAGTGAA
                ******************* ****************************************

h               GCATCCCTAAGTCTGTTGTGAGTGACATGGTGATTAGAATTTAGATTAGGGAAAACACAT
c               GCATCCCTAAGTCTGTTGTGAGTGACATGGTGATTAGAATTTAGATTAGGGAAAACACAT
m               GCATCCCTAAGTCTGTTGTGAGTGACATGGTGATTAGAATTTAGATTAGGGAAAACACGT
r               GCATCCCTAAGTCTGTTGTGAGTGACACGGTGATTAGAATTTAGATTAGGGAAAACACAT
                *************************** ****************************** *

                                               1   1
h               TCTGTACTTTATCAAGGACAGTAATTAGTTCAGTCAGTAAAAGTTGATTACAAGTAACGA
c               TCTGTACTTTATCAAGGACAGTAATTAGTTCAGTCAGTAAAAGTTGATTACAAGTAACGA
m               TCTGTACTTTATCAAGGACAGTAATTAGTTCAGTCAGTAAAAGTTGATTACAAGTAACGA
r               TCTGTACTTTATCAAGGACAGTAATTAGTTCAGTCAGTAAAAGTTGATTACAAGTAACGA
                ************************************************************

h               TAAAGTTAGATTTTT-AGTACTTAATTTTAAAATTTCTTTATTAAATAGTAAATTTGTTC
c               TAAAGTTAGATTTTT-AGTACTTAATTTTAAAATTTCTTTATTAAATAGTAAATTTGTTC
m               TAAAGTTAGATTTTT-AGTACTTAATTTTAAAATTTCTTTATTAAATAGCAAATTTGTCC
r               TAAAGTTAGATTTTTTAGTACTTAATTTTAAAATTTCTTTATTAAATAGCAAATTTGTTC
                *************** ********************************* ******** *

h               TTAATATAAATGGTACTATCTACTCTTGTTTTATTGCATATCAGAATAATTAAAAGAACA
c               TTAATATAAATGGTACTATCTACTCTTGTTTTATTGCATATCAGAATAATTAAAAGAACA
m               TTAATATAAATGGTACTATCTACACTTGTTTTATTGCCTATCAGAATAATTTAAAGAACA
r               TTAATATAAACGGTGCTCTCTACACTCGTTTTATTGCCTATCAGAATAATTTAAAGGACA
                ********** *** ** ***** ** ********** ************* **** ***

h               ATGTGTTCAATGCAGAGTATAATCAAGCGATTTATATATTATGGGTAAGTTTCTATGTAG
c               ATGTGTTCAATGCAGAGTATAATCAGGCGATTTATATATTATGGGTAAGTTTCTATGTAG
m               ATGTGTCCAATGCAGAGTATAATCAGGCGGTTTGTGTATTGTGGGTAAGTTCCTATGTAG
r               ATGTGTCCAAAGCACAGCGTAACCAGGCGGTTTATGTATTGTGGGTAAGTTCCTAGGTAG
                ****** *** *** **  *** ** *** *** * **** ********** *** ****

h               TTTTTAGAAAATGTAATATTATATAATCTTTCTGATGTCATCATTTTT-AGAAGTTTAAT
c               TTTTTAGAAAATGTAATATTATATAATCTTTCTGATGTCATCATTTTT-AGAAGTTTAAT
m               CTTTTAGGAAATGCAATATTATGTAATCTGTCTTATGTCATCATTTTTCAAAAGCTTAGC
r               CTTTTAGGAAACATAATATTATGTAATCCGTCTTATGTCATCATTTTTCGAAAGTTTAGC
                 ****** ***   ******** *****  *** **************   *** ***

h               AATT-GTCACACTGT---GATAGGT------TATTGTGGAAGTTTTCTAGTGTTCAGGTA
c               AATT-GTCACACTGT---GATAGGT------TATTGTGGAAGTTTTCTAGTGTTCAGGTA
m               AATTTTTCACACCGT---GACAACTTGATGATGTTGTGGGAATGTTCTAGTGTTCAGGTA
r               AATTTGTCACACCGTCGTGACAGCTCAATGATGTTGTGGGAACGTTCTAGTGTTCAGGTA
                ****  ****** **   ** *  *      * ****** *   ****************
```

TCF4 exon 10a

```
        Aligned regions
h       chr18:51139749-51140227
c       chr18:51711821-51712299
m       chr18:69752920-69753388
r       chr18:66269376-66269845
exon 10a sequence identity between h and m: 99%

h               TCTCGCGTATGCATTAGGATTTCGAGCTCCATCAGGGCCGGGCTGCCTTCCAGCTCCGCT
c               TCTCGCGTATGCATTAGGATTTCGAGCTCCATCAGGGCCGGGCTGCCTTCCAGCTCCGCT
m               TCTCGAGTGCTC----GGCTGCGGCGCTGCAC--AGGCCGGGCCGCCATCCAGC----CT
r               TCTCGAGTGCTC----GGCTGCGGCGCTGCAC--AGGCCGGGCCGCCATCCAGC----CT
                ***** **   *    ** *   * *** **    ******** *** ******    **

h               GCGCAAAGTTGAGCGTCTGACAGCAGCGCCGCGGCCTCCCCC-GCCCGCCAGGAATGGTC
c               GCGCAAAGTTGAGCGTCTGACAGCAGCGCCGCGGCCTCCCCC-GCCCGCCAGGAATGGTC
m               GCGCGAACTTGAGCGTCTGACAGCAGCGCCGGGGCCTCCCCC-GCCCGCCAGGAATGGTC
r               GCGCGAACTTGAGCGTCTGACAGCAGCGCCGCGGCCTCCCCCCGCCCGCCAGGAATGGTC
                **** ** *********************** ********** *****************
                      1             4                1412   13      4     1
h               TCTTCCTGCTTTGCATATTCACCACGCTTGGCCCGGCCATATGGGAAAATGCAACTGAAG
c               TCTTCCTGCTTTGCATATTCACCACGCTTGGCCCGGCCATATGGGAAAATGCAACTGAAG
m               TCTTCCTGCTTTGCATATTCACCACGCTTGGCCCGGCCATATGGGAAAATGCAACTGAAG
r               TCTTCCTGCTTTGCATATTCACCACGCTTGGCCCGGCCATATGGGAAAATGCAACTGAAG
                ************************************************************
                     3 12 21  2                               1  1
h               GAATTGTTGTGAAAAAAGGGACAGCGAGTTTGAAATAAAAGTTGTTAGAGTGGTACTGAG
c               GAATTGTTGTGAAAAAAGGGACAGCGAGTTTGAAATAAAAGTTGTTAGAGTGGTACTGAG
m               GAATTGTTGTGAAAAAAGGGACAGCGAGTTTGAAATAAAAGTTGTTAGAGTGGTACTGAG
r               GAATTGTTGTGAAAAAAGGGACAGCGAGTTTGAAATAAAAGTTGTTAGAGTGGTACTGAG
                ************************************************************
                   2 1              0.726     1                0.491     1
h               GAGAAAAAAGAATCCGAGACCATGTACTGCGCATACACAATCCCGGGCATGGCGGCAAC
c               GAGAAAAAAGAATCCGAGACCATGTACTGCGCATACACAATCCCGGGCATGGCGGCAAC
m               GAGAAAAAAGAATCCGAGGCCATGTACTGCGCATACACCATCCCGGGCATGGCGGCAAC
r               GAGAAAAAAGAATCCGAGGCCATGTACTGCGCATACACCATCCCGGGCATGGCGGCAAC
                ****************** ******************* ********************
                     4 0.440
h               TCTTTGATGTACTACTATAATGGGAAAGCGGTAAGCGAATGCGGGCTGCGAGGGGGATTT
c               TCTTTGATGTACTACTATAATGGGAAAGCGGTAAGCGAATGCGGGCTGCGAGGGGGATTT
m               TCTTTGATGTACTACTATAATGGGAAAGCGGTAAGCGAACTCGGGCTGCGAGGGGCATTG
r               TCTTTGATGTACTACTATAATGGGAAAGCGGTAAGCGAACTCGGGCTGCGAGGGGCATTG
                ***************************************  ************** ***

h               TTGACAAGTTTTATTGTCGCAGGGATGAGCATCCTAGGTGGTGATCATTGTTTGCAAAGT
c               TTGACAAGTTTTATTGTCGCAGGGATGAGCATCCTAGGTGGTGATCATTGTTTGCAAAGT
m               GGGACAAGTTTTATGGTCACAGGGATGAGCCTTCTAGGCGAGGAGCATTGTTTGCTAAGT
r               GGGACAAGTTTTATGGTCACAGGGATGAGCCTTCTAGGCGGGGAGCATTGTTTGCGAAGT
                  ************ *** *********** * ***** *  ** ********** ****

h               TGTGCAGGACTGCCGCACCTTCCTGGGTGCCATGAAATATTTATCATTTGCCAATCCTAA
c               TGTGCAGGACTGCCGCACCTTCCTGGGTGCCATGAAATATTTATCATTTGCCAATCCTAA
m               TGTGCAGGACTGCCGCACCTTTCTCGGTACCATGAAATATTTATCATCTGCGAGTCCTAG
r               TGTGCAGGACTGCCGCACCTTTCTCGGTGCCATGAAATATTTATCATCTGCGAGTCCTAG
                ********************* ** *** ****************** *** * *****
```

TCF4 exon 10b

Aligned regions

h chr18:51139327-51139667
c chr18:51711398-51711739
m chr18:69753455-69753790
r chr18:66269909-66270246

exon 10b sequence identity between h and m: 88%

```
h               GAGCTTTAGAGA-GTGGATCCTAGAGCGAAA-GTTTGCAGTCAGGTAAAGTTGAGAGGTG
c               GAGCTTTAGAGA-GTGGATCCTAGAGCGAAA-GTTTGCAGTCAGGTAAAGTTGAGAGGTG
m               AAGCCCCTGAAA-GTAGATCCCAGAGAGAAA-GTTTGCAGTCAGGTGAACCTAGGGGGTT
r               AAGCTCCTGAAAAGTAGATCCCAGAGAGAAAAGTTTGCAGTCAGGTGAACTTAGGAGGTT
                 ***    ** * ** ***** **** **** ************** **  *  * ***

h               GTATGATTT-------TTTTTTTTT------AAAGAGTTTTGG--AAAATTCTTATTCCT
c               GTATGATTT-------TTTTTTTTTT-----AAAGAGTTTTGG--AAAATTCTTATTCCT
m               GTACGATTTAAAATAATACTAATTTTTAAAAAAGGACTTTTGGCAAAA-TTATTTTTGCT
r               GTACGATTTAAAATAATACTAATTTT---AAAAGGATTTTTGGCAAAAATCCTTTTTGCT
                *** *****       *  *  ***      ** ** ******  *** *  ** ** **
                                                         1
h               TC-CAAATGACTGTTAGAAAGGAAAAGCAGATATGAGAAGCAGAATTGAGAAAGCCCAAG
c               TC-CAAATGACTGTTAGAAAGGAAAAGCAGATATGAGAAGCAGAATTGAGAAAGCCCAAG
m               CT-CAAATGATTG----GAAGGAGGAGCAGAT-TATGAAG-AGAACTGAGAAAACCCAAG
r               TTTCAAATGATTG----GAAGGAGGAGCAGAT-TATGAAG-AGAACCGAGAAAGCCCAAG
                   ******* **     *****  ******* *  **** ****  ****** ******

h               TTAGGCTGAGAGGAAATTTGGGGTAAGTTTCTCCCTGGATTGAGGTTTTGTGATATCTAA
c               TTAGGCTGAGAGGAAATTTGGGGTAAGTTTCTCCCTGGATTGAGGTTTTGTGATATCTGA
m               TTAGCCTGAGAGAAAATTGGGGGTAAGTTTCACTCCGGATTGAGGTTTTGTGCTGTCTGA
r               TTAGCCTGAGAGAAAATTGGGGGTAAGTTTCTCTCCGGATTGAGGGTCTGTACTATCTAA
                **** ******* ***** ************ * * ********* * ***  * *** *

h               AGTCTTACTAAGTTGGAAGCTTGAGTGCAGTGGATAGAGTGGAGAGAACCTGAAAGGTTA
c               AGTCTTACTAAGTTGGAAGCTTGAGTGCAGTGGATAGAGTGGAGAGAACCTGAAAGGTTA
m               AGTCTTACTAAGTTGGAAACC--------ATGGG--GAGTGGACAATGGATAAGAGATTA
r               AGTCTTACTAAGTTGGAAACC--------ATGGG--GAGTGGACAAGAGATAAGAGATTA
                ****************** *          ***   ******* *     * * ** ***

h               AAATAGAGCAAACACATAAAA-CTGAGAGATCAAATTAAGGAGTAGATTATATAAGCTTA
c               AAATAGAGCAAACACATAAAA-CTGAGAGATCAAATTAAGGAGTAGATTATATAAGCTTA
m               AAATAGACCAAAAACTTAAAA-CGGACATATCTGAT---GGAGTAGATTATATAAGCTTG
r               AAATAGACCAAAAACTTAAAAACGGACATATCTGAT---GGAGTAGATTATATAAGCTTG
                ******* **** ** ***** * ** * ***  **   ********************
```

TCF4 exon 10c

Aligned regions

h chr18:51120517-51121010
m chr18:69771959-69772448
r chr18:66,287976-66288465

exon 10c sequence identity between h and m: 85%
Note: the respective chimp sequence is not available in the latest March 2006 assembly.

```
h               CTAAACGGGCTCCAGATGCAGCTTTATCCAGGG-GACGCCTCATGCCTGGTCATTTGGGT
m               CTAAGGATGGAGTGGATGTGGCTCACACCAGGATGATGCCTGGAGCCTGGTCATTTGGGT
r               CTAGCCATGTTCTAGACGGGGCTCACACCAGGATGATGCCCAGAGCCTGGTCATTTGGGT
                ***     *     ** *  ***    *****  ** ***    ****************

h               GGCTGGGCTTGTCCCACCTCCCCTTCTTCATGCCCTCCTCCCGGTCTCATCCTAGGCCCT
m               GTCTGGGCCTGTCCCACCTCCCCTCCC-CATGTACTCCTCCCACCCTCCTCCTGGGCCCT
r               GGCTGGGCCTGTCCCACCTCCCCTCCC-CATGTACTCCTCCCAGCCTTCTCCTAGGCCCT
                * ****** *************** *  ****  ********   **  **** ******
```

```
                                                     1    1
h         CCCACCCCTAGGCCAGCCACAGGCCTCACAGCGGACATCTGCGGGTCTGAGTTTCATATA
m         CCCACCCCCAAGCCAGTCAGGGGCCTCACTGCAGACATCTGCGGGCATGAGGCACATATA
r         CCCACCCCCAAGTCAGTCCCAGGCCTTCCTGCGGACATCTGCAGGTCTGAGGCACATGTA
          ******** * * *** *   *****  * ** ********* **  ****   *** **

h         TCCAGCATGAAGTTCCCTCTCTCCCTCCCATTATGAATTCTGGGCTGTGGTCCTGCTTGC
m         CCCAGCCTGAGGTTGCGTCTCTGCCTCCCGCTATGAATTCTGGGCTGTGGTCCTGCTCCC
r         CCCAGCCCGAGGTTGTCTCCCTGCCTCCCGCTATGAATTCTGGGCTGTGGTCCTGCTCTG
           *****  ** ***   ** ** ******  **************************
                                     1
h         --TCCTTGTGTGTCAGATTGAAGTTCTGATGCTGTCAGCCATGTCTGACCAATCAGCAAA
m         TGCTCTTGCCTGTCAGATTGCAATTCTGATGCTGTCAGCCATGCCTGACCAATCAGCAAA
r         -GTCCTTGTCCGTCAGATTGCAATTCTGATGCTGTCAGCCACGCCTGACCAATCAGAGAG
              ****   ********* * ****************** * ************  *
                     0.514                               1  1
h         CAACACCACAGGATGAAATTTAAACAATGCAGATGCTCAGATACAGGGCTATGTTGCCTG
m         CACCACCACGGGATGAGACTTAAACAATGCAGATGCTCAAAGGCAGGGTTATGTCTCGTG
r         CGCCGCTGCGGGATGAAACTTAAACAATGCAGATGCTCCAATGCAGGGTTATGTTTCGTG
          *  * *  * ****** * *******************  *  ***** *****  * **

h         GACCACGAAGGGAAAGCAGAGGTAAGGTCAGAGTCTCCCCAAAGTGGTCTATCCTGAGCC
m         GACCACGAAGGGAGAGCAGAGGTAAGGTCTCAGTCTCCCCAAAGTGGTCTGTCCCCAGCC
r         GAGCACGGAGGGAGAGCAGAGGTAAGGTCTCAGTTTCCCCAAAGTGGCCTGTCCCCAGCC
          ** **** ***** ***************  *** ************ ** ***  ****
h         TGCCTGACTTGACACCTCTTGGGCTGCACTG-TGC--TGCTTTGACTTGTATGTTACCCA
m         TGCTTGGTCTGACACTCCCTAAGCGACACTGCCGC--TGCTCTGCCTCGG-TGCCACC--
r         TGCTCGGCCTGACACTTCCTAAGTCATACTGCTGCCCTGCTCTGCCTTGT-TGTCACC--
          ***  *   ******  * *  *    ****  **  **** ** ** *  **  ***

h         TTTGCTTTGCACTACTTCTGTCTTCCCTGGCATTCACCAGAACTTTCATTTTAATTCGCT
m         --TGCTTTCCCCTGCCTTT--CTTCCCTGCCACTCACCAGAACTTTTGTCTGCGTCC--C
r         --TGCTTTCCC-TACCTTT--CTTCCCTGCCACTCACCAGAACTTTTGTTCCCATCC--C
            ****** *  * * * *  ******** ** *************  *     * *
```
