# Supplementary material for: Functional Diversity of Human Basic Helix-Loop-Helix Transcription Factor TCF4 Isoforms Generated by Alternative 5′ Exon Usage and Splicing
Source: PLoS One. 2011 Jul 15;6(7):e22138. doi: 10.1371/journal.pone.0022138 (PMC3137626; doi:10.1371/journal.pone.0022138)
Supplement: Table S1 — Accession numbers of representative mRNA or EST sequences for alternative TCF4 transcripts and complete coding sequences of TCF4 isoforms cloned in full-length in this study. (PDF) [file pone.0022138.s003.pdf]

Supporting Table S1. Accession numbers of representative mRNA or EST sequences for alternative *TCF4* transcripts and complete coding sequences of TCF4 isoforms cloned in full-length in this study.

Transcripts with alternative 5´ exons

Transcript	Human	Mouse
1a	CD557562	
1aΔ3	FR748202	
1b(1)	BC031056	
1b(1)Δ3	DB055535	
1b(1,2)	FR748203	
1b(1,2)Δ3	DB443719	
1b(2)	DB040143	
1b(2)Δ3	FR748204	
3a	FR748205	
3aΔ3	DC398422	
3b	AK315074	AK133885
3bΔ3	AK299169	AK051958
3c	DB106801	AK081012
3cΔ3	FR748206	
3d	M74719	X91753
3dΔ3	BP206418	
4a-I	BP293388	
4a-II	DC337265	
4a-III	DC390184	
4b	BI544467	
4c	DC358747	CJ115804
5a-I	AU279553	
5a-II	FR748207	
5b	AK096862	
5c	DC398249	
7a-I	DC358599	
7a-II	AK300612	BY247629
7a-III	DC375084	
7b-I	AK095041	
7b-II	DC350124	CD350230
8a	AK316165	BY286412
8b-I	FR748208	
8b-II	AK300636	BY252182
8c-I	DC326149	
8c-II	FR748209<br>CA393351	BB663894
8d	BP230382	
10a	AK300038	U16321
10b	BP214032	BU058820
10c	DA664480	BY333068

Transcripts with alternative internal splicing

Transcript	Human	Mouse
8-9 present	DB106801	AK133885
Δ8-9	AK315074	
8 (acceptor I) 130 bp	AK096862	AK133885
8 (acceptor II) 127 bp	AK095041	BY259217
15 (acceptor I) 77 bp	M74718	AK133885
15 (acceptor II) 74 bp	CN404346	BE652641
18^- (donor I) 151 bp	AK316165	U16321
18^+ (donor II) 163 bp	AK315074	AK133885

Full-length coding sequences

Isoform	Human
TCF4-B^+	FR748210
TCF4-B^-	FR748211
TCF4-B^+Δ	FR748212
TCF4-B^-Δ	FR748213
TCF4-C^-	FR748214
TCF4-C^-Δ	FR748215
TCF4-E^-	FR748216
TCF4-F^-	FR748217
TCF4-G^-	FR748219
TCF4-D^-	FR748218
TCF4-A^+	FR748220
TCF4-A^-	FR748221
TCF4-H^-	FR748222
TCF4-I^-	FR748223

The nucleotide sequences deposited by this study are under Accession Numbers FR748202-FR748223.
