# Supplementary material for: Functional Diversity of Human Basic Helix-Loop-Helix Transcription Factor TCF4 Isoforms Generated by Alternative 5′ Exon Usage and Splicing
Source: PLoS One. 2011 Jul 15;6(7):e22138. doi: 10.1371/journal.pone.0022138 (PMC3137626; doi:10.1371/journal.pone.0022138)
Supplement: Table S2 — List of oligonucleotides used in this study. (PDF) [file pone.0022138.s004.pdf]

Supporting Table S2. List of oligonucleotides used in this study.

Specificity	Forward primer (5´- 3´)	Reverse primer (5´- 3´)	Products (bp)	Tm (°C)	Notes
1a-8	GAGGCATGAGAGCGAGCAAG	GTTTGGTGGGCGAAAGGGTTCC	536, 444	60	37
1-5	AGAACATTATCTCTTGGACGGGCA	GGACCCTGAGCTACTTCTGTCTTC	260, 168 (381, 289)	59	35
1b,2-5	AGGAGGCCCTTGGAGATGGAG	GGACCCTGAGCTACTTCTGTCTTC	330, 238	61	35
3a-8	AGACAGGAATCTTCACCATGTGTG	GTTTGGTGGGCGAAAGGGTTCC	530, 438	59	37
3b-5	CCCGGATGTGAATGGATTACAATG	GGACCCTGAGCTACTTCTGTCTTC	689, 597	59	40
3c-5	CTGTTCAGGCTTCAGATTGTAACTG	GGACCCTGAGCTACTTCTGTCTTC	239, 147	57	37
3d-5	GGTTAGACATGAACGCCGCCTC	GGACCCTGAGCTACTTCTGTCTTC	258, 166	59	37
4aI-5	CCCCTTTCTGCTTGTTGCACTTTC	GGACCCTGAGCTACTTCTGTCTTC	169	59	40
4aIII-11	GCTCTCCTGTTAGAGACGAAATGAG	TGGATGCAGGCTACAGTAGCTG	739	59	40
4b-5	GAGATGGAAATGGACATTGCCACG	GGACCCTGAGCTACTTCTGTCTTC	138	57	40
4c-11	AGCTCTGAAGGCACAAGTGTC	TGGATGCAGGCTACAGTAGCTG	762	57	37
5a-11	GGGAGCTTTCCCTGACATTCTTTC	TGGATGCAGGCTACAGTAGCTG	665	59	40
5b-11	CATGGAGCACAGGAGAGTAACAAG	TGGATGCAGGCTACAGTAGCTG	800	59	40
5c-11	CTGGAGGGTAAATACGGTCATTCC	TGGATGCAGGCTACAGTAGCTG	743	55	45
7a-8	CCTCGGCGTGCTACCCATAAG	GTTTGGTGGGCGAAAGGGTTCC	185, 159, 147	57	37
7b-8	TCTTGCTTGCATACATTGCCAG	GTTTGGTGGGCGAAAGGGTTCC	160, 262	59	40
8a-11	TTACAGCCATTCCAGCGCAAC	TGGATGCAGGCTACAGTAGCTG	444	59	40
8b-11	CTTGTGAAAGCATCATCCAGACCG	TGGATGCAGGCTACAGTAGCTG	542	57	40
8cI-11	AGAGGTGGATGGATTGGCATGTG	TGGATGCAGGCTACAGTAGCTG	457, 956	59	40
8cII-11	CAGCTGAAATGATTCCCCACTGTG	TGGATGCAGGCTACAGTAGCTG	439	57	40
8d-11	GTCAGTAAAAGTTGATTACAAGTAACG	TGGATGCAGGCTACAGTAGCTG	599	57	40
10a-11	ACCATGTACTGCGCATACACAATC	TGGATGCAGGCTACAGTAGCTG	300	57	37
10b-11	GAGAAAGCCCAAGTTAGGCTGAG	TGGATGCAGGCTACAGTAGCTG	263	59	40
10c-11	GCTATGTTGCCTGGACCACGA	TGGATGCAGGCTACAGTAGCTG	262	57	40
5-11	AGAAGACAGAAGTAGCTCAGGGTC	TGGATGCAGGCTACAGTAGCTG	628, 448	57	35
10-20	TATGCTCCATCAGCAAGCACTG	CATCTGTCCCATGTGATTCGATGCG	1461(BglII:1090+371), 1449	57	35
10-11	TATGCTCCATCAGCAAGCACTG	TGGATGCAGGCTACAGTAGCTG	225	61	q
17-18	GCCATTCTCTTCTGCCAAACCA	TCATCACCCTCGTCATCGGATT	172	60	q
19-20	TCCTCAGTCTGGAGCAGCAA	TCGATGCGTCTCCCATTCCA	144	56	q

Specificity	Forward primer (5´- 3´)	Reverse primer (5´- 3´)	Products (bp)	Tm (°C)	Notes
HPRT1	CAGTCCCAGCGTCGTGATTA	AGCAAGTCTTTCAGTCCTGTC	141	58	q
GAPDH*	TGCACCACCAACTGCTTAGC	GGCATGGACTGTGGTCATGAG	87	60	q
HMBS*	GGCAATGCGGCTGCAA	GGGTACCCACGCGAATCAC	64	58	q
SDHA*	TGGGAACAAGAGGGCATCTG	CCACCACTGCATCAAATTCATG	86	58	q/32
UBC	GATTTGGGTCGCAGTTCTTG	CTCGATGGTGTCACTGGGCTCA	122	58	q
3-11(RPA)	caccATGCATCACCAACAGCGAATGG	TGGATGCAGGCTACAGTAGCTG	777+4 (597+4)		c
10-16(ISH)	CTCCATCAGCAAGCACTG	GTTCCATACCCTGAGCCCAGAC	760		c
8-9(NLS)	ctaagatctaCCCCGAAGGAGGCCTCTTCA	cacggtaccGGAGGAACTTTTCGAACTTTC	68+19		c
TCF4 NLS m1	TCTAGCAATAATCCCgcAgcGgcGCCTCTTCA CAGTAGT	ACTACTGTGAAGAGGCgcCgcTgcGGGATT ATTGCTAGA			m
TCF4 NLS m2	CATGGAGGTACAGACAgcGgcAGTTgcAgcAG TTCCTCCAGGTTTG	CAAACCTGGAGGAACTgcTgcAACTgcCgc TGTCTGTACCTCCATG			m
3×μE5	CTAGAGATCTGAACACCTGCAAGAACACCTGC AAGAACACCTGCAAG	GATCCTTGCAGGTGTTCTTGCAGGTGTTCT TGCAGGTGTTCAGATCTCTAGAGCT			a

Specificity	Forward primer (5´- 3´)	Reverse primer (5´- 3´)	Isoform	Notes
3	caccATGCATCACCAACAGCGAATGG		TCF4-B	c
4	tcgaattcaccATGTTTTCACCTCCTGTGAGC		TCF4-C	c
3c	caccATGCAACGGGCAAAAACTGAAC		TCF4-E	c
5b,5	caccATGGAAGAGGACAGCAGAGATGTA		TCF4-F	c
8	tcgaattcaccATGGATATGGGCAACCCAG		TCF4-D	c
7b	caccATGAAGGATATTTTTTTCCAGTTTATC		TCF4-G	c
10a	cACCATGTACTGCGCATACACAATC		TCF4-A	c
10c	caccATGAAATTTAAACAATGCAGATGCTC		TCF4-H	c
10,11	cacCATGCAAGATGGCCATCACAG		TCF4-I	c
20		CATCTGTCCCATGTGATTCGATGCG	all	c
20stop		ctgagctcTcACATCTGTCCCATGTGATTC	all	c
21		ccactcgaGCGTCTGCGATTCATAACTAC	all	c

Specificity	Forward siRNA (5´- 3´)	Reverse siRNA (5´- 3´)	Species specificity	Notes
12 (I)	ACCAUUACAGCACCUCUUCtt	GAAGAGGUGCUGUAAUGGUTT	human and mouse	si
12 (II)	CGGAACAGACAGUAUAAUGtt	CAUUAUACUGUCUGUUCCGTT	mouse	si
20	GAAGGGAGGAAGAGAAGGUtt	ACCUUCUCUUCCUCCCUUCTT	human and mouse	si

The oligonucleotides were used for detection of TCF4 transcripts and housekeeping gene transcripts by RT-PCR, amplification of TCF4 coding sequences and fragments for cRNA probe synthesis for cloning, site-directed mutagenesis of TCF4 coding sequence, and annealing of μE5 E-boxes. TCF4 primer pairs are designated with the exon range spanned by the amplified product. For clarity TCF4 PCR product lengths are given only for transcripts containing longer variants of exons 8 and 15. In the ‘Notes’ column, the number of cycles is given for the primer pairs used for end-point PCR analysis; primers used for quantitative PCR, cloning or annealing are indicated with ‘q’ , ‘c’ or ‘a’, respectively; siRNAs are indicated with ‘si’. Primers marked with asterisk (*) have been described before [55]. Nucleotides in lowercase indicate adapter sequences (CACC Kozak consensus and restiction sites) or mutated positions.
